# ﻿Morphological and molecular re-assessment of European and Levantine species of the genus *Hortiboletus* (*Boletaceae*)

**DOI:** 10.3897/imafungus.16.144731

**Published:** 2025-06-12

**Authors:** Alona Yu. Biketova, Tatyana Yu. Svetasheva, Andy F. S. Taylor, Giampaolo Simonini, Matteo Gelardi, Olga V. Morozova, Elias Polemis, José A. Muñoz, László Albert, Salvatore Saitta, Solomon P. Wasser, Eviatar Nevo, Georgios I. Zervakis, Alfredo Vizzini, Bálint Dima

**Affiliations:** 1 Jodrell Laboratory, Royal Botanic Gardens, Kew, Richmond TW9 3DS, UK Jodrell Laboratory, Royal Botanic Gardens, Kew Richmond United Kingdom; 2 Mycological Society of Israel, P.O. Box 164, Pardesiya 42815, Israel University of Haifa Haifa Israel; 3 Institute of Biochemistry, Biological Research Center of the Eötvös Loránd Research Network, Temesvári Blvd. 62, Szeged H-6726, Hungary Mycological Society of Israel Pardesiya Israel; 4 Institute of Evolution and Department of Evolutionary & Environmental Biology, University of Haifa, Aba Khoushi Ave. 199, Mt. Carmel, Haifa 3498838, Israel Institute of Biochemistry, Biological Research Center of the Eötvös Loránd Research Network Szeged Hungary; 5 Tula State Lev Tolstoy Pedagogical University, 125 Lenin Ave., Tula 300026, Russia Tula State Lev Tolstoy Pedagogical University Tula Russia; 6 The James Hutton Institute, Aberdeen AB15 8QH, UK The James Hutton Institute Aberdeen United Kingdom; 7 Via bell’aria 8, Reggio nell’Emilia I-42121, Italy Unaffiliated Reggio nell’Emilia Italy; 8 Via dei Barattoli 3A, Anguillara Sabazia I-00061, RM, Italy Unaffiliated Anguillara Sabazia Italy; 9 Komarov Botanical Institute of the Russian Academy of Sciences, 2 Prof. Popov Str., Saint Petersburg 197376, Russia Komarov Botanical Institute of the Russian Academy of Sciences Saint Petersburg Russia; 10 Agricultural University of Athens, Laboratory of General and Agricultural Microbiology, Iera Odos 75, Athens 11855, Greece Agricultural University of Athens Athens Greece; 11 Sociedad Micológica Barakaldo, P.O. Box 100, 48902 Barakaldo, Vizcaya, Spain Sociedad Micológica Barakaldo Barakaldo Spain; 12 Hungarian Mycological Society, Pázmány Péter s. 1/c, Budapest H-1117, Hungary Hungarian Mycological Society Budapest Hungary; 13 Via Di Anfuso pal. 28, n. 221, Messina I-98147, Italy Unaffiliated Messina Italy; 14 M.G. Kholodny Institute of Botany, National Academy of Sciences of Ukraine, Tereshchenkivska St. 2, Kiev 01601, Ukraine M.G. Kholodny Institute of Botany, National Academy of Sciences of Ukraine Kiev Ukraine; 15 Department of Life Sciences and Systems Biology, University of Turin, Viale P.A. Mattioli 25, Turin I-10125, Italy University of Turin Turin Italy; 16 Department of Plant Anatomy, Institute of Biology, Eötvös Loránd University, Pázmány Péter sétány 1/c, H-1117, Budapest, Hungary Eötvös Loránd University Budapest Hungary

**Keywords:** *Agaricomycetes*, biogeography, *Boletales* taxonomy, diversity, phylogeny, xerocomoid fungi

## Abstract

*Hortiboletus* (the former *Xerocomusrubellus* species complex) is one of the most taxonomically critical and difficult genera for species identification in the family *Boletaceae*. Here, we provide a detailed morphological and molecular re-assessment of European and Levantine species of *Hortiboletus*. A new species, *H.hershenzoniae*, is described from Israel. It is sister to *H.engelii* and associated with the evergreen oak *Quercuscalliprinos* and potentially also with *Q.ithaburensis*. Based on the sequence retrieved from INSDC, this species is also found in Lebanon. Accurate morphological descriptions, comprehensive sampling, type studies, biogeography, macro- and microphotographs and a historical overview on the nomenclatural issues surrounding *H.rubellus*, *H.bubalinus*, *H.engelii*, and *H.hershenzoniae* are given. An epitype collection is designated for *H.rubellus*. A key is provided for identification of the European and Levantine taxa. In addition, we propose a novel taxonomic combination *Hortiboletusflavorubellus*, which is conspecific with Boletusrubellusvar.flammeus, based on the DNA barcoding and phylogenetic analysis of type material. *Boletusharrisonii* is also shown to be conspecific with *H.campestris*. A multilocus phylogenetic analysis of four markers (ITS, LSU, *tef1-α*, and *rpb2*) reveals that *Hortiboletus* is a sister genus to *Xerocomellus*. Using the Genealogical Concordance Phylogenetic Species Recognition method, at least 19 phylogenetic species and eight putative phylogenetic species of the genus *Hortiboletus* can be delimited. Based on multilocus analysis, it contains from 24 to 25 species-level clades worldwide, 17 out of which represent known species, one newly described and potentially six to seven undescribed species. Tandem repeat insertions within the ITS region (both in ITS1 and ITS2) are reported for the first time, not only in the genus *Hortiboletus*, but in the entire subfamily *Boletoideae*. Their identification and characterisation were based on Tandem Repeat Finder analysis and visual assessment of the ITS alignment.

## ﻿Introduction

The genus *Hortiboletus* Simonini, Vizzini & Gelardi, corresponding to the former *Xerocomusrubellus* species complex (= “rubellus” clade in Nuhn et al. (2013)), is one of the taxonomically most challenging and insufficiently explored xerocomoid genera. *Hortiboletus* species are probably among the most difficult to identify in the family *Boletaceae*. They exhibit a high intraspecific variability in terms of colours of the basidiomes and microscopic structures. At the same time, various *Hortiboletus* species look very similar, while apparently lacking pronounced morpho-anatomical differences. In addition, much confusion over the identification of individual *Hortiboletus* species exists and a shared concept of their taxonomic limits has not yet been achieved.

*Hortiboletus* was described as a separate genus by Simonini, Vizzini & Gelardi in 2015, with *Boletusrubellus* Krombh. as the type species (Vizzini 2015). It was segregated from the earlier described genus *Xerocomellus* Šutara (type species *Boletuschrysenteron* Bull.), which also included *B.rubellus* and *B.engelii* Hlaváček (Šutara 2008). Later, three other species, *B.engelii*, *B.campestris* A.H. Sm. & Thiers, and *B.bubalinus* Oolbekk. & Duin were also transferred to *Hortiboletus* (Biketova 2015; Dima 2015). A monophyly of this genus was supported by multiple phylogenetic analyses (Taylor et al. 2001; Nuhn et al. 2013; Wu et al. 2014, 2016; Das et al. 2016; Frank et al. 2020; Mao et al. 2023; Pham et al. 2024; Wang et al. 2024). According to Nuhn et al. (2013), in their general inclusive multigene phylogenetic analysis of the family *Boletaceae*, the “rubellus” clade belongs to the “anaxoboletus” group, which is congruent to the major clade of the subfamily *Boletoideae* in the study by Wu et al. (2014).

*Hortiboletus* is characterised by small to medium-sized basidiomes with a xerocomoid habitus, dry pileus and stipe surfaces not staining blue-green with ammonia, subtomentose to finely squamulose pileus, sometimes tending to become cracked-areolate at maturity and with colours ranging from ochraceous, pinkish-red, scarlet red, vinaceous red, reddish-brown to brown, yellow-olive tubular hymenophore with tubes no longer than 15 mm at maturity, smooth or pruinose to fibrillose-striate stipe tinged yellow to pinkish-red or brownish, frequent presence of very fine carrot-orange to orange-red (also referred as flame-red or vermillion-red) punctuations in the stipe base context which can sometimes be scattered or absent, tissues unchanging to turning light blue when injured, especially in the pileus and the connection zone with the stipe. Main microscopical features are ellipsoid-fusiform, smooth basidiospores, palisadoderm or physalo-palisadoderm pileipellis consisting of erect and (sub)parallel, generally short and frequently encrusted hyphae, hymenophoral trama intermediate between the “*Boletus*-type” and the “*Phylloporus*-type”, lateral stipe stratum absent or only rarely present and, in this case, of the “boletoid type”, fertile caulohymenium, inamyloid tissues or, at most, with a fleeting amyloid reaction, and absence of clamp connections. Members of *Hortiboletus* form ectomycorrhizal (EcM) association with some species of *Betulaceae*, *Corylaceae*, *Fagaceae*, *Malvaceae*, *Pinaceae*, and *Salicaceae* (Ladurner 2001; Lannoy and Estadès 2001; Taylor et al. 2001, 2002; Ladurner and Simonini 2003; Peintner et al. 2003; Estadès and Lannoy 2004; Hills 2008; Muñoz et al. 2008; Šutara 2008; Vizzini 2015; Bessette et al. 2016; Wu et al. 2016; Naseer et al. 2019; Frank et al. 2020; Klofac and Krisai-Greilhuber 2020; Xie et al. 2020; Lebeuf et al. 2021; Mao et al. 2023; Pham et al. 2024).

However, several of the aforementioned features are shared by other xerocomoid genera (*Xerocomus* Quél. s. str., *Xerocomellus*, *Rheubarbariboletus* Vizzini, Simonini & Gelardi, etc.) and cannot be used to unequivocally separate them from each other. Using data from molecularly confirmed specimens of *Hortiboletus*, it is now possible to highlight the main diagnostic traits which are related to the basidiospores morphology and which are useful to distinguish the genus *Hortiboletus* from the other xerocomoid genera: smooth basidiospores which are neither striate nor truncate and with an average spore quotient (Qm) usually lower than or up to 2.6. In addition, *Hortiboletus* species appear to have a preference for anthropogenic environments such as rural or urban areas (parks, gardens, lawns, etc.), which can be explained by a somewhat higher ecological plasticity than in other bolete genera.

Currently, *Hortiboletus* includes sixteen species described worldwide, with only three species known from Europe, i.e. *Hortiboletusrubellus* (Krombh.) Simonini, Vizzini & Gelardi, *H.bubalinus* (Oolbekk. & Duin) L. Albert & Dima, and *H.engelii* (Hlaváček) Biketova & Wasser (Redeuilh and Simonini 1995, 1999a; Biketova 2015; Dima 2015; Vizzini 2015; GBIF 2024). However, it is very difficult to ascertain the correct identity of the European *Hortiboletus* species as described in past morphologically-based literature, as in most cases, they may represent a mixture of different taxa. Thirteen species are known from other regions of the world: a) East, South-East, and South Asia: *H.amygdalinus* Xue T. Zhu & Zhu L. Yang (China and Korea), *H.arduinus* N.K. Zeng, H.J. Xie & W.F. Lin (China), *H.indorubellus* K. Das, D. Chakr., Baghela, S.K. Singh & Dentinger (India and Pakistan), *H.kohistanensis* A. Naseer, S. Sarwar & A.N. Khalid (Pakistan and China), *H.napaeus* N.K. Zeng, H.J. Xie, S. Jiang & Zhi Q. Liang (China), *H.rubroreticulatus* T.H.G. Pham, O.V. Morozova, E.S. Popov (Vietnam), *H.rufosquamosus* L. Fan, N. Mao & T.Y. Zhao (China), *H.rupicapreus* Svetash., A.V. Alexandrova, O.V. Morozova & T.H.G. Pham (Vietnam), *H.sinorubellus* Yang Wang, X. Chen, B. Zhang & Y. Li (China), *H.subpaludosus* (W.F. Chiu) Xue T. Zhu & Zhu L. Yang (China), and *H.tomentosus* L. Fan, N. Mao & T.Y. Zhao (China); b) North America: *H.campestris* (A.H. Sm. & Thiers) Biketova & Wasser (USA, Canada, and Mexico), and *H.coccyginus* (Thiers) C.F. Schwarz, N. Siegel & J.L. Frank (USA) (Smith and Thiers 1971; Biketova 2015; Das et al. 2016; Wu et al. 2016; Naseer et al. 2019; Frank et al. 2020; Xie et al. 2020; Kim et al. 2021; Lebeuf et al. 2021; Saldivar 2021; Mao et al. 2023; GBIF 2024; Pham et al. 2024; Wang et al. 2024).

The aims of this study were to: 1) perform a taxonomic revision of the genus *Hortiboletus* by examining species occurring in Europe and Levant (in the narrower sense), including relevant type studies; 2) clarify the morphological variability, biogeography, and ecology of the European species; 3) introduce a new species to science, namely *Hortiboletushershenzoniae*; 4) assess the phylogenetic relationships among *Hortiboletus* species with a worldwide distribution through single-locus (ITS, LSU, *tef1-α*, and *rpb2*) and multilocus analyses; 5) delimit species of the genus by the Genealogical Concordance Phylogenetic Species Recognition method, and 6) conduct an analysis of tandem repeats in the ITS region.

## ﻿Materials and methods

### ﻿Collection sites and sampling

Studied specimens are deposited in ACAM, GB, H, IB, K, KW, L, LD, LE, MCVE, MICH, O, SAF, and TUR fungal collections (acronyms follow Index Herbariorum (Thiers 2025)) and in the personal fungaria (collectors’ numbers) “AB”, “AH”, “AL”, “AT”, “BD”, “EP”, “FP”, ‘‘GK’’, “GS”, “JAM”, ‘‘MG’’, and “VGy” maintained by Alona Yu. Biketova, Alan E. Hills, László Albert, Andy F.S. Taylor, Bálint Dima, Elias Polemis, Péter Finy, George Konstantinidis, Giampaolo Simonini, Jose A. Muñoz, Matteo Gelardi, and György Vrba, respectively. In the field, latitude, longitude, and elevation were determined with a Global Positioning System (GPS) receiver. Author citations follow the Authors of Fungal Names (Index Fungorum 2025). Taxonomic novelties and the epitype are registered in MycoBank (MycoBank 2025). The distribution range and data on occurrences have been checked in specialised literature, GBIF (GBIF 2024), iNaturalist (iNaturalist 2024), Mushroom Observer (Mushroom Observer 2024), and Botanical Collections (Botanical Collections 2024). An asterisk (*) indicates disputed territories with partially recognised independence (not UN members). Abbreviation “GP” (genetically proven) indicates collections (as well as associated ectomycorrhizal partners and distribution of *Hortiboletus* species by countries), whose identification was verified using molecular methods and bioinformatics (BLAST and phylogenetic analyses). The metadata of the majority of studied collections is given as follows: COUNTRY, First-Order Administrative Division [or the Nature Region of Israel according to Zohary (1966)]: locality, coordinates (DMS), elevation in m (above sea level, if available), number of basidiomes and/or primordia collected, habitat with putative host plants, date (dd.mm.yyyy), leg. = legitur, det. = determinator, collection number (additional collection number; GP if available). “Ibid.” means that a specimen was collected in the same administrative location as the previous collection with fully listed location data, but not necessarily in the same spot.

### ﻿Morphological studies

A total of 242 collections representing the four European and Levantine species of *Hortiboletus* were studied. Macroscopic characteristics, macro-chemical spot-test reactions (Melzer’s reagent, 20% KOH, 25% NH_4_OH, 10% FeSO_4_, and 10% H_2_SO_4_) were examined on fresh basidiomes. For some collections, macro-morphological characteristics of the specimens were also assessed using Carl Zeiss Stemi DV4, Carl Zeiss Axio Zoom V.16, and Wild M3C stereomicroscopes. For characterisation of colour, three colour charts were used: Ridgway (1912) for the majority of collections of *H.rubellus*, *H.engelii*, and *H.bubalinus*, BFF (Anonymous 1969) and OAC (Anonymous 2004) for *H.hershenzoniae*. Microscopic features were observed from revived dried material using the following light microscopes: Carl Zeiss Axiostar 1122-100, Jenaoptik Jenamed, Nikon Eclipse E200, Carl Zeiss Axioscope A1 with AxioCam 1Cc 3 camera, Carl Zeiss AxioImager A2 under bright field and DIC with AxioCam 305 colour camera, and Olympus BX43 with ADF PRO08 camera. Sections were rehydrated either in distilled water, L4 solution (according to Moser 1983), 5% potassium hydroxide (KOH), 3% ammonia (NH_3_), or 1% Congo red (in ammonia solution) for measurements of all structures. Colours and pigments were described after examination in water and 5% KOH. Measurements were made on dried material after soaking in 5% KOH for 5 min, at 1000× using a calibrated ocular micrometre as well as camera software with the appropriate calibration installed. Basidiospores (when possible at least 31 per collection) were measured from the hymenophore of mature basidiomes; dimensions of the average values are given as (minimum of the averages) average ± standard deviation (maximum of the averages), Q = (minimum of the averages) average quotient (length/width ratio) ± standard deviation of the averages (maximum of the averages), and average spore volume approximated as a rotation ellipsoid [Vm = (π.L.W^2^)/6 ± standard deviation of the averages]. Average sizes of terminal elements of pileipellis (calculated when possible at least on 31 elements) were also calculated for each collection; dimensions are given as (minimum of the averages) average ± standard deviation of the averages (maximum of the averages) and Qm = average quotient (length/width ratio) ± standard deviation of the averages. The notation [n/m/p] indicates that measurements were made on “n” randomly selected structures from “m” basidiomes of “p” collections. Two couples of morphometric variables (length and width of the spores, length and width of the pileipellis terminal elements) were measured and statistically analysed as samples of a bivariate Gaussian distribution, through the isoprobability (confidence) ellipse method. In the field of the considered variables, the ellipse represents the locus where the pairs of the variables’ values have an identical probability (68%) to occur. All the points inside of the ellipse represent pairs of the variables having a probability of occurrence greater than 68%. The centre of the ellipse (the average value of length and width), represents the most probable value among the pairs of the variables.

As for the other microscopical elements, except for basidiospores and terminal elements of pileipellis, dimensions are given as (minimum) average ± standard deviation (maximum) of the whole of the samples. The width of each cystidium and basidium was measured at the widest part and the length was measured from the apex (sterigmata excluded) to the basal septum. Sections of the pileipellis were taken mid-way between the centre and margin of the pileus and sections of the stipitipellis were taken between the upper third and the middle part along the longitudinal axis of the stipe. Iodine, metachromatic and cyanophilic reactions were tested by staining the basidiospores in Melzer’s reagent (Imler 1950), Brilliant Cresyl blue, and Cotton blue in lactophenol (Clémençon 2009), respectively. To test the amyloidity according to Imler’s process (“amyloid reaction”, Imler (1950)), a sample of approx. 1 mm^3^, taken from the stipe base context, was soaked for 3 min in Melzer’s reagent and then washed carefully in a chloral hydrate solution. This procedure was repeated until the yellowish colour of the Melzer’s solution disappears. Then the sampled tissue was carefully squeezed between slide and cover glass and observed under a light microscope. The result is called “amyloid” if a clear blue to blue-grey reaction was observed.

The basidiospores of selected collections (*H.rubellus* MCVE31743, *H.engelii* K-M000170238, *H.hershenzoniae* K-M001435594 and K-M001435706) were also analysed using scanning electron microscopes Zeiss Ultra-Plus VP FEG-SEM (operated at 2 kV) equipped with an Oxford X-Max 80 mm^2^SDD detector and Zeiss Ultra-Plus FEG-SEM HR (operated at 4–5 kV) equipped with an Oxford EDS SDD detector. Small parts of the hymenophore material with basidiospores were mounted on a metal stub using carbon double adhesive tape, then coated with a thin layer of gold/palladium (60/40% alloy) using Polaron SEM Coating Unit E5150 (Polaron Equipment Ltd.), and analysed using the SEM.

### ﻿DNA extraction, PCR amplification and DNA sequencing

Total genomic DNA was extracted from dried basidiomes using the NucleoSpin Plant II kit with minor modifications. The following primers were used: ITS1F, ITS1, ITS4B, and ITS4 for nuclear ribosomal internal transcribed spacer (ITS) (White et al. 1990; Gardes and Bruns 1993), LR0R, LR5, and LR7 for nuclear large subunit ribosomal DNA (LSU) (Vilgalys and Hester 1990; Cubeta et al. 1991; Hopple and Vilgalys 1999), EF1-983F and EF1-2218R for translation elongation factor 1-α gene (*tef1-α*) (Rehner and Buckley 2005), and bRPB2-6F and bRPB2-7R for DNA-directed RNA polymerase II subunit 2 gene (*rpb2*) (Matheny 2005).

For ITS and LSU, PCR was carried out under the following parameters: initial denaturation at 95 °C for 5 min; followed by 35 cycles of denaturing at 95 °C for 30 s, annealing at 53 °C (or 55 °C for ITS1F and ITS4B) for 30 s, extension at 72 °C for 1 min; and final extension at 72 °C for 7 min. For *tef1-a*: initial denaturation at 95 °C for 3 min; then 8 cycles: 98 °C for 20 s, 60 °C for 40 s, 72 °C for 2 min; then 36 cycles: 98 °C for 20 s, 53 °C for 1.30 min, 72 °C for 2 min; and a final extension step of 72 °C for 10 min. For *rpb2*: initial denaturation at 95 °C for 5 min; followed by 40 cycles: 95 °C for 30 s, 58 °C for 40 s, 72 °C for 1 min; and final extension step at 72 °C for 8 min. For generating ITS sequences of some *H.rubellus* and *H.bubalinus* collections, cloning with Invitrogen™ TOPO™ TA Cloning™ kit was used.

Sequences were manually edited and assembled using Sequencher 4.1.4 and 5.4.6 (Gene Codes Corporation, Ann Arbor, Michigan, USA) and CodonCode Aligner 9.0.2 (CodonCode Corp., Centerville, Massachusetts, USA) software.

ITS, LSU, *tef1-α*, and *rpb2* sequences of holotypes of *Boletuscampestris*, *B.harrisonii*, *B.flavorubellus*, and B.rubellusvar.flammeus were bioinformatically extracted from the genome assemblies published by Tremble et al. (2024) using PathRacer (Shlemov and Korobeynikov 2019) and BLASTn (Altschul et al. 1990; Camacho et al. 2009).

A total of 133 collections were sequenced; however, not all sequences were of sufficient quality to be used in phylogenetic analysis, but sufficient for DNA-based identification (indicated as “GP” in the list of examined material). Good quality sequences generated (both newly sequenced and bioinformatically extracted) for this study and used in phylogeny were submitted to GenBank or UNITE and their accession numbers are cited in Suppl. material 1: table S1.

### ﻿Sequence alignment, phylogenetic analysis and species delimitation

A total of 479 sequences from 287 specimens (collections), including 170 newly generated (117 ITS, 9 ITS-LSU, 12 LSU, 16 *tef1-α*, 15 *rpb2*) and 298 retrieved from INSDC and UNITE databases (126 ITS, 2 ITS-LSU, 72 LSU, 57 *tef1-α*, 41 *rpb2*), were used in the phylogenetic analyses (Table 1). *Imleriabadia* was chosen as an outgroup taxon for multilocus (ITS, LSU, *tef1-α*, and *rpb2*) and single-locus phylogenies.

**Table 1.** Characterization of the tandem repeat insertions within the ITS region of *Hortiboletus* spp. Only consensus patterns with alignment score ≥ 50 are shown. ND = not detected. The most frequently detected consensus patterns of tandem repeats are highlighted in bold. **Tandem repeat in ITS1 region was detected when the length of insertion was minimum 26 bp using Tandem Repeats Finder v.4.09.

Taxon (insertion region)	N of sequences examined	Total length of ITS (bp)	Insertion size (bp)	Nucleotide similarity within each species	Alignment parameters (match - mismatch - indels)	Tandem repeats information			
						Consensus pattern of tandem repeats (size in bp)	Copy number	Percent of matches	Percent of indels
*H.amygdalinus* (ITS2)	2	840	167	100%	2-7-7	**GGTTGGCTTAGCTATTAGTCGGTCGTGAGGCCGACGAACGCGGTCGACTTGGAAAG (56)**	2.9	88	2
			181	100%	2-3-5	**GGACTTGGCAAGGGTTGGCTTAGCTATTAGTCGGTCGTGAGGCCGACGAACGCGGTC (57)**	3.2	84	5
*H.bubalinus* (ITS1)**	31	900–913	14–26	100%	2-7-7	AC (2)	7–13	100	0
*H.bubalinus* (ITS2)	43		225–228	>98%		GGGACTCGAGCGAAGGGTCGGCTTAGCTATTAGTTGGTCGTGAGGCCAGCGAACGCGGTCGGGCTGGGTCTCGAGCTTCA (80)	2.8	81–86	5–7
						GGGACTCGAGCGAAGGGTCGGCTTAGCTATTAGTTGGTCGTGAGGCCAGCGAACGCGGTCGGCTGTGGGTCTCGAGCTTCA (81)			
						**GGGACTCGAGCGAAGGGTCGGCTTAGCTATTAGTTGGTCGTGAGGCCAGCGAACGCGGTCGGGCTGTGGGTCTCGAGCTTCA (82)**			
						GGGACTCGAGCGAAGGGTCGGCTTAGCTATTAGTTGGTCGTGAGGCCAGCGAACGCGGTCGGGCTGTGGGTCTCGAGCTTTCA (83)			
			231–253	>98%	2-3-5	**TTCGGGACTCGAGCGAAGGGTCGGCTTAGCTATTAGTTGGTCGTGAGGCCAGCGAACGCGGTCGGGCTGGGTCGTCGAG (79)**	2.9–3.1	80–82	6–8
						TTTGGGACTCGAGCGAAGGGTCGGCTTAGCTATTAGTTGGTCGTGAGGCCAGCGAACGCGGTCGGGCTGGGTCGTCGAGC (80)			
						TTTGGGACTCGAGCGAAGGGTCGGCTTAGCTATTAGTTGGTCGTGAGGCCAGCGAACGCGGTCGGGCTGTGGGTCTCGAGC (81)			
*H.kohistanensis* (ITS2)	2	N/A	156–157	97%	2-7-7	**GGTTGGCTTAGCTATTAGTCGGTCGCGAGGCCGACGAACGCGGTCGACTTGGWAAAG (57)**	2.8	91–95	1
			168–169	97%	2-3-5	**GACTTGGCAAGGGTTGGCTTAGCTATTAGTCGGTCGCGAGGCCGACGAACGCGGTC (56)**	3	88–91	1–2
*H.rubellus* (ITS1)	30	853–864	14–26	>96%	2-7-7	AC (2)	7–13	92–100	0–8
*H.rubellus* (ITS2)	33		166–167	>99%		**TTGGCTTAGCTATTAGTCGGTCGTGAGGCCAACGAACGCGGTCGACCTTGGAAAACGTCGAA (62)**	2.7	83–84	3
			165	>99%	2-3-5	ND or **GGCTTAGCTATTAGTCGGTCGTGAGGCCAACGAACGCGGTCGACCTGTCAGAAAACGTCGAATC (64)**	2.6	83–85	1
			170	>99%		**GGATTGGCTTAGCTATTAGTCGGTCGTGAGGCCAACGAACGCGGTCGACCTTAGAAAACGTC (62)**	2.7	81–83	1
*H.rubroreticulatus* (ITS2)	1	817	133	N/A	2-7-7	TTCCCCTAGTAACTGCGAGTGAAGCGGGAAGAGCTCAAATTTCGAATCTGGCGGTCTCTTTGGCCG (66)	2	100	0
			156	N/A	2-3-5		2.4	90	2
*H.rufosquamosus* (ITS2)	6	850	ND	ND	2-7-7	ND	ND	ND	ND
			134	100%	2-3-5	**GGGTCGGCTTAGCTATTAGTTGGTCGTGAGGCCGACGAACGCGGGTCGACTCGGCAAAACGTT (63)**	2.1	90	4
*H.rupicapreus* (ITS2)	2	838–842	ND	ND	2-7-7	ND	ND	ND	ND
			ND-141	N/A	2-3-5	ND or TCGGGATCCGAGCGAAGGTCAGCCTTCAGCTATTAAGTCGGTCGCAAGACCGAACGAACGCGGCCGGCTGGATCC (75)	1.9	75	4
*Hortiboletus* sp. 4 (ITS2)	2	N/A	239–240	95%	2-7-7	GGGACTCGAGCGAAGGGTCGGCTTAGCTATTAGTTGGTCGTGAGGCCAGCGAACGCGGTCGGCTGGGTTTCGAGTTCTG (79)	2.9–3	87	4–5
						ACTCGAGCGAAAGGTCGGCTTAGCTATTAGTTGGTCGTGAGGCCAGCGAACGCGGTCAGGCTGGGTTTCGAGTTCAGGGA (80)			
			240–251	95%	2-3-5	GGGACTCGAGCGAAGGGTCGGCTTAGCTATTAGTTGGTCGTGAGGCCAGCGAACGCGGTCGGCTGGGTTTCGAGTTCTG (79)	3–3.1	85–87	4–6
						GGGACTCGAGCGAAAGGTCGGCTTAGCTATTAGTTGGTCGTGAGGCCAGCGAACGCGGTCAGGCTGGGTTTCGAGTCTCA (80)			
*Hortiboletus* sp. 5 (ITS2)	2	912–913	245	100%	2-7-7	**GGGACTCGAGCGAAGGTCGGCTTAGCTATTAGTTGGTCGTGAGGCCAGCGAACGCGGTCGGGCTGGGTTTCGAGTTCAG (79)**	3	85	5
			245	100%	2-3-5		3	85	5
*Hortiboletus* sp. 6 (ITS2)	5	900–901	235–236	>97%	2-7-7	**GACTCGAGCGAAAGGTTGGCTTAGCTATTAGTTGGTCGTGAGGCCAGCGAACGCGGTCGGGCTGGGTTTCGAGTTCAGG (79)**	3	86–89	3–5
			244–245	>97%	2-3-5	**GACTCGAGCGAAAGGTTGGCTTAGCTATTAGTTGGTCGTGAGGCCAGCGAACGCGGTCGGGCTGGGTTTCGAGTTCAGG (79)**	3.1	85–88	2–5
						**GACTCGAGCGAAAGGTTGGCTTAGCTATTAGTTGGTCGTGAGGCCAGCGAACGCGGTCGGGCTGGGTTTCGAGTCTCAGG (80)**			
			ND-103	N/A		**ND** or GGCTGGCGAACGCGGTCG (18)	5.3	50	19

Four alignments were generated for ITS (algorithm E-INS-i), LSU (algorithm G-INS-i), *tef1-α* (algorithm FFT-NS-i), and *rpb2* (algorithm FFT-NS-i) datasets with MAFFT 7 (Katoh et al. 2019). Data for each locus were manually adjusted and concatenated in MEGA 6.06 (Tamura et al. 2013) and SeaView 5 (Gouy et al. 2021). Single-character positions (predominantly results of sequencing errors) were manually removed: 35 in ITS, 8 in LSU, 5 in *tef1-α*, and 3 in *rpb2* alignment (51 in total). Phylogenetic reconstructions were performed using the Maximum Likelihood (ML) and Bayesian Inference (BI) methods of analysis. In all ML and BI analyses, except the ITS ones, the general time reversible with rates that vary over sites according to the gamma model (GTR+G) was employed. In both ML and BI analyses of ITS, the GTR+G+I model was employed. For ITS and multilocus analyses of both ML and BI, partitioned models (ITS1, 5.8S, ITS2, LSU, *tef1-α*, and *rpb2*) have been applied.

The ML phylogenetic analyses were run in the raxmlGUI 2.0 (Edler et al. 2020), which implements the search protocol of Stamatakis et al. (2014), estimating unique model parameters for alignment and using 1000 rapid bootstrap replicates and branch lengths saved in the bootstrap trees (BS brL enabled).

BI analysis was performed with MrBayes 3.2.7a software (Ronquist et al. 2012), under the described model. Analysis was performed with two parallel searches and four chains, with 5.75 mln generations for ITS, 4 mln for LSU, 2 mln for *tef1-α* and *rpb2*, and 10 mln for multilocus phylogeny, as well as a sampling frequency of every 1000^th^ generation. Tracer 1.6.0 (Rambaut et al. 2014) was used to evaluate the quality of a sample from the posterior and the continuous parameters, using effective sample size (ESS). A burn-in of 25% for all analyses was applied.

Only bootstrap support (BS) over 50% and posterior probability (PP) values exceeding 0.8 are reported in the resulting trees. A clade was considered strongly supported if it received bootstrap support (BS) equal to or greater than 90% and posterior probability (PP) equal to or greater than 0.99, a medium supported clade if BS = 70–89 and PP = 0.95–0.98, and a weakly supported clade if BS = 50–69 and PP = 0.80–0.94, respectively. Clades with strong and medium statistical support were considered as well supported. Branch lengths were estimated as mean values over the sampled trees. The final trees were edited in FigTree v.1.4.4 (Rambaut 2018), MEGA 12.00 (Kumar et al. 2024), and Inkscape v.1.3.2. Multilocus alignment and non-collapsed single-locus phylogenetic trees are available in Suppl. materials 2, 3.

The Genealogical Concordance Phylogenetic Species Recognition (GCPSR) method (Taylor et al. 2000) was employed to delimit phylogenetic species using three independently inherited loci (ITS-LSU, *tef1-α*, and *rpb2*). We followed the criteria of Dettman et al. (2003): a clade was recognized as an independent evolutionary lineage if it was well supported (when BS ≥ 70% and PP ≥ 0.95) by at least one single-locus genealogy and was not contradicted by any other single-locus genealogy. When a well-supported independent evolutionary lineage had only a single locus or two inherited together loci (ITS and LSU) available for phylogenetic reconstruction, we considered it as a putative phylogenetic species. When designating independent evolutionary lineages as phylogenetic species, the concatenated four-locus analysis was also considered.

### ﻿Detection and characterisation of insertion sequences within ITS

Initially, long insertions within the ITS2 region of several species were visually detected after alignment. Then all ITS sequences, which were used in phylogenetic analysis and did not have more than 5% of non-ATGC bases in target regions, were searched for the presence of more-or-less regular and continuous ITS tandem repeats using Tandem Repeats Finder (TRF) software v. 4.09 with default settings (alignment parameters 2-7-7: match = 2, mismatch = 7, indel = 7) (Benson 1999). After this primary TRF search, we analysed only fragments of the ITS2 region containing the long insertion (position 770 until the end of ITS – 1169 in the alignment) using the same software with alignment parameters 2-3-5 for more sensitive detection of tandem repeats.

The boundaries of the insertions were determined, based on the results of alignment and TRF analyses. Core consensus sequences (core motifs) detected with TRF were compared and analysed using the BLASTn algorithm (https://blast.ncbi.nlm.nih.gov/).

### ﻿Abbreviations

**BFF**: “Flora of British fungi: Colour identification chart” (Anonymous 1969); **BI**: Bayesian Inference; **BS**: bootstrap support; **DNA**: deoxyribonucleic acid; **FEG**: field emission gun; **GCPSR**: Genealogical Concordance Phylogenetic Species Recognition; **INSDC**: International Nucleotide Sequence Database Collaboration; **ITS**: nuclear ribosomal internal transcribed spacer; **LSU**: nuclear large subunit ribosomal DNA; **ML**: Maximum Likelihood; mln: million(s); min: minute(s); **OAC**: “The Online Auction Color Chart” (Anonymous 2004); **Q**: quotient (length/width) spore ratio; **Qm**: average quotient (length/width ratio); **PP**: posterior probability; ***rpb2***: DNA-directed RNA polymerase II subunit 2 gene; **s**: second(s); **SDD**: silicon drift detector; **SEM**: scanning electron microscope(y); ***tef1-α***: translation elongation factor 1-α gene; **TRF**: Tandem Repeats Finder; **UNITE**: User-friendly Nordic ITS Ectomycorrhizal Database; **Vm**: average spore volume.

## ﻿Results

### ﻿Phylogenetic analysis and species delimitation

**Figure 1.**
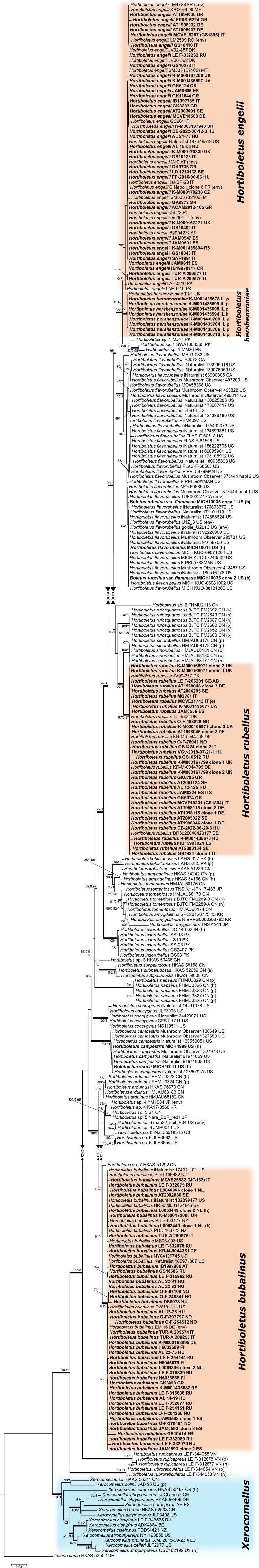
ML phylogenetic tree of *Hortiboletus* generated from a multilocus (ITS + LSU + *tef1*-*a* + *rpb2*) dataset. BS values ≥ 50% and PP values ≥ 0.8 indicated at nodes. Thickened branches indicate high statistical support (BS ≥ 70% and PP ≥ 0.95). Species names of collections follow current identification, except original names of types of *Boletusharrisonii* and B.rubellusvar.flammeus. *Hortiboletus* species present in Europe and Levant are indicated by light red-coloured fields. Collections with newly-generated sequences are indicated in bold. Two-letter country codes (ISO 3166-1 alpha-2) denote the origin of specimens. At the end of the annotations to some collections, abbreviations are indicated in brackets: e – epitype, h – holotype, p – paratype, env – environmental sample.

**Figure 2.**
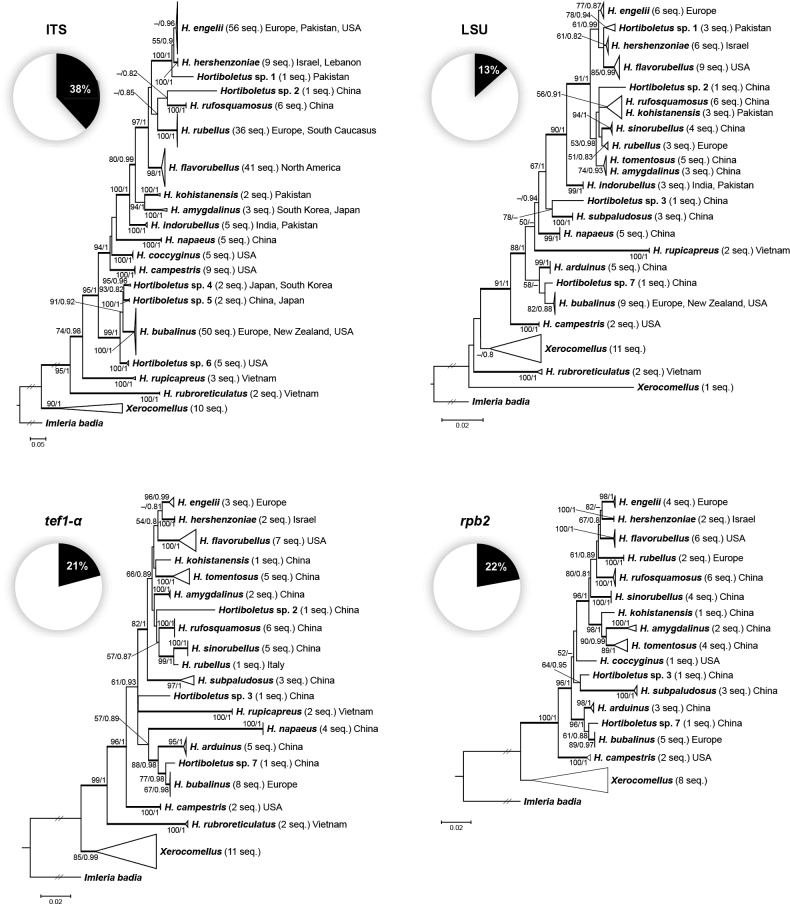
Phylogenetic trees of *Hortiboletus* from separate ML analyses of each locus: ITS, LSU, *tef1-a*, and *rpb2*. BS values ≥ 50% and PP values ≥ 0.8 are indicated at nodes. Thickened branches indicate high statistical support (BS ≥ 70% and PP ≥ 0.95). Pie charts underneath each label are the percent of total characters in the alignment that are parsimony-informative.

The aligned ITS matrix contained a total of 254 sequences from 241 specimens and a total of 1169 aligned bases with gaps, including 446 (38.2%) parsimony-informative sites. The LSU matrix contained 95 sequences from 94 specimens and 929 aligned bases with gaps, including 125 (13.5%) parsimony-informative sites. The *tef1-α* matrix contained 73 sequences from 73 specimens and 1100 aligned bases with gaps, including 229 (20.8%) parsimony-informative sites. The *rpb2* matrix contained 56 sequences from 56 specimens and 777 aligned bases with gaps, including 172 (22.1%) parsimony-informative sites. Additionally, a multilocus matrix was composed of 479 sequences (255 ITS, 95 LSU, 73 *tef1-α*, 56 *rpb2*) from 287 specimens and 3975 aligned bases with gaps, including 972 (24.5%) parsimony informative sites (Suppl. material 2). The ML and BI analyses generated almost identical tree topologies for each of all five phylogenies with minimal variation in statistical support values; thus, ML trees with both BS and PP values were selected for the purposes of display (Figs 1, 2).

In vast majority of phylogenetic reconstructions (multilocus as well as single-locus ones: ITS, *tef1-α*, and *rpb2*), except the LSU analysis, the genus *Hortiboletus* is supported as monophyletic and its separation from *Xerocomellus* received high support (Figs 1, 2). Based on multilocus analysis, we recognised from 24 to 25 well-supported species-level terminal clades in *Hortiboletus*, which represent 17 previously described (including a newly-transferred species), one newly-described, and six to seven unnamed/undescribed species (Fig. 1). Using the Genealogical Phylogenetic Species Recognition method, at least 19 phylogenetic species and eight putative phylogenetic species of the genus *Hortiboletus* can be delimited (Fig. 2). Terminal clades of *Hortiboletus* sp. 4 (Japan, South Korea), *Hortiboletus* sp. 5 (China, Japan), and *Hortiboletus* sp. 6 (USA), which are sister to *H.bubalinus*, are represented only by ITS sequences. Clade *Hortiboletus* sp. 1 (Pakistan) is represented by ITS and LSU sequences. Clade *Hortiboletus* sp. 7 (China) is represented by LSU, *tef1-α*, and *rpb2* sequences of a single collection (HKAS 51292) and also cluster as a sister one to *H.bubalinus*. Therefore, *Hortiboletus* sp. 7 may be conspecific with *Hortiboletus* sp. 4 or *Hortiboletus* sp. 5. That is why we count from six to seven potentially new species-level clades.

In the present study, we further focus on the three European species and the description of one new species (*H.hershenzoniae*) from Israel and Lebanon, which is a sister species of *H.engelii*. The two other European species, *H.bubalinus* and *H.rubellus*, are rather distant from each other and form sister relationships with other extralimital species (Figs 1, 2).

### ﻿Characterisation of tandem repeat insertions within ITS

The ITS region is longer than expected in a number of *Hortiboletus* species (up to 913 bp in *H.bubalinus* and *Hortiboletus* sp. 5) due to an unusually long insertion in the ITS2 region. In the *Hortiboletus* species where minisatellite-like insertions were not detected, the length of the entire ITS region was 679–778 bp and, in those with insertions, 817–913 bp. The alignment also contained a short AC-reach insert (microsatellite) in the ITS1. These AC repeats have especially high intraspecific variability in *H.rubellus* and *H.bubalinus*, which vary in length within the species from 14 to 26 bp and even within the same genome (up to 12 bp). TRF analysis was able to detect these repeats only when the minimal length was 26 bp (Table 1).

TRF analysis detected long tandem repeat insertions in the ITS2 region of ten species, namely *H.amygdalinus*, *H.bubalinus*, *H.kohistanensis*, *H.rubellus*, *H.rubroreticulatus*, *H.rufosquamosus*, *H.rupicapreus*, *Hortiboletus* sp. 4, *Hortiboletus* sp. 5, and *Hortiboletus* sp. 6, ranging from 133 to 253 bp with 2.0–3.2 copy number of continuous motifs in almost all cases (Table 1). These minisatellite-like insertions in ITS2 region were highly conserved within each species, but divergent between species. In most cases, the core consensus sequences ranged from 56 to 82 bp in length and they are homologous between species with 89–97% similarity. However, in one case of analysis with the ITS2 insertion of *Hortiboletus* sp. 6 (iNaturalist 55519315) when alignment parameters were 2-3-5, one of two identified tandem repeats was 103 bp with a core sequence of 18 bp and 5.3 copy number.

In the ITS2 of *H.rubroreticulatus*, TRF analysis detected the same 66 bp-long core sequence by using both full-length ITS sequences with alignment parameters 2-7-7 (match - mismatch - indel) and a partial ITS2 sequence contains minisatellite-like insertion with alignment parameters 2-3-5, but the differences were in a copy number of core sequence – 2 and 2.4, respectively and, therefore, in length of the entire insertion – 133 and 156 bp. The core sequence of *H.rubroreticulatus* was not homological to those of other *Hortiboletus* species. In the case of *Hortiboletus* sp. 5, results of both types of analysis were the same. However, in most cases, both setting types of TRF analysis detected somewhat different core sequences and ITS2 insertions with difference in length ranging from 1 to 25 bp.

In the ITS2 region of *H.rufosquamosus* and *H.rupicapreus*, TRF analysis was able to detect tandem repeats only when alignment parameters were set to 2-3-5. Therefore, we see a necessity in running TRF analysis with a range of alignment parameters to obtain a more comprehensive evaluation of tandem repeats in each set of sequences.

## ﻿Taxonomy and distribution

### ﻿ Hortiboletus

Taxon classificationFungiBoletalesBoletaceae

Simonini, Vizzini & Gelardi, in Vizzini, Index Fungorum 244: 1 (2015), emend. Biketova, Simonini, Gelardi & Vizzini

61E9D3D7-35D5-5356-A081-D753158DF915

#### Emended description.

Basidiomes stipitate-pileate with tubular-poroid hymenophore, epigeal, xerocomoid, small or medium-sized. Pileus surface dry, matt, subtomentose to finely squamulose pileus, in some species, gradually cracking with age and becoming areolate-rimose along the margin or overall, exposing the pale or reddish context in the cracks, colours ranging from ochraceous, pinkish-red, scarlet red, vinaceous red, reddish-brown to brown, not showing a blue-green reaction with NH_3_. Stipe fibrous, surface dry, pruinose or fibrillose, yellow to pinkish-red or brownish. Context whitish to light yellow, sometimes flesh pink or reddish-pink in the upper part of the pileus, pale to dull yellowish-brown in the stipe, usually bruising pale blue or, less frequently, deep blue in the pileus and the connection zone with the stipe; the stipe base context sometimes showing minute orange to orange-red dots. Taste mild. Spore print olive-brown. Pileipellis a palisadoderm or physalo-palisadoderm of more or less encrusted hyphae, or rarely a trichoderm. Basidiospores smooth, never striate nor truncate at apex, ellipsoid-fusiform, with an average spore quotient (Qm) lower than or up to 2.6. Hymenophoral trama bilateral divergent intermediate between the “*Boletus*-type” and the “*Phylloporus*-type”. Lateral stipe stratum absent or rarely present of the boletoid type. Fertile caulohymenium. Hymenophore tissue and basidiospores inamyloid or with a fleeting amyloid reaction; and negative amyloid reaction at stipe base according to Imler’s procedure. Basidiomes grow preferably in light, grassy forests, forest edges, meadows, and disturbed places, such as urban or rural areas.

The etymology of the generic name *Hortiboletus* derives from the Latin word *hortus* (garden) and refers to the growth of these boletes in gardens.

#### Generic type.

*Boletusrubellus* Krombh.

#### Notes.

**Figure 3.**
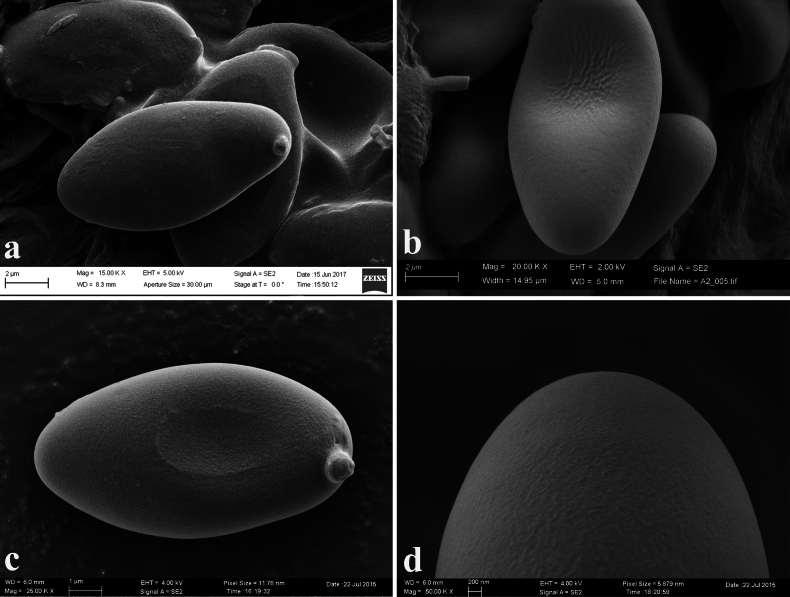
Scanning electron micrographs of *Hortiboletus* basidiospores: **a***H.rubellus* (MCVE31743, epitype); **b***H.engelii* (K-M000170238); **c, d***H.hershenzoniae* (K-M001435594, holotype). Photos: (**a**) A. Yu. Biketova and Y. Pollak; (**b**) A. Yu. Biketova and B. Dobrić; (**c, d**) A. Yu. Biketova and M. Kalina.

According to the original description, the genus *Hortiboletus* is close to *Xerocomellus*, differing by the smooth basidiospores without any ornamentation (both under light microscopy and SEM, see Fig. 3), the average spore quotient (Qm) that is lower than or equal to 2.5 and the presence of tiny carrot-orange to orange-red dots in the context at the stipe base (Vizzini 2015). Nevertheless, recent studies show that not all representatives of the genus *Hortiboletus* share the aforementioned morphological features, based on which they were separated from *Xerocomellus*. For example, the average spore quotient of some collections of *H.bubalinus* and *H.subpaludosus* may be higher than 2.5 (Ladurner and Simonini 2003; Wu et al. 2016). In addition, *Hortiboletus* species do not have peculiar microchemical reactions (iodine, metachromatic, cyanophilic). Amyloid reaction of the stipe base trama is negative according to Imler’s procedure (Imler 1950).

Possible and fickle appearance of orange-red dots (“*ponctuation de Redeuilh*”; Simonini (1998)) in the context of the stipe base is a feature which discriminates European and Levantine members of the genus *Hortiboletus* from those of *Xerocomellus* and *Rheubarbariboletus*. However, it is probably a character shared by only a few species in this genus. For the majority of Asian and American *Hortiboletus* species, such as *H.amygdalinus*, *H.arduinus*, *H.campestris*, *H.coccyginus*, *H.flavorubellus*, *H.napaeus*, *H.rubroreticulatus*, *H.rufosquamosus*, *H.rupicapreus*, *H.sinorubellus*, *H.subpaludosus*, and *H.tomentosus*, no red dots are reported to occur in the stipe base (Thiers and Smith 1966; Smith and Thiers 1971; Wu et al. 2016; Frank et al. 2020; Xie et al. 2020; Lebeuf et al. 2021; Mao et al. 2023; Pham et al. 2024; Wang et al. 2024). Moreover, in many cases (especially in wet conditions), they are not observable even in *H.rubellus*, *H.engelii*, and *H.hershenzoniae*. In most specimens of *H.hershenzoniae*, they are visible only using a stereomicroscope. As already pointed out by Taylor and Eberhardt (2006) and Gelardi (2010), our present investigation shows that dispersed red dots may be rarely present in some collections of *H.bubalinus*.

*Hortiboletus* exsiccata, like those of *Xerocomellus*, gradually lose their bright colour becoming brownish upon drying; but red dots in the context of the stipe base, if present in fresh basidiomes, retain their bright colour for decades. In addition, the stipe base context in *Rheubarbariboletus* remains yellow-orange to orange. Probably there are similar pigments present in the stipe base of both genera, but further investigation will be necessary to confirm it.

*Hortiboletus* species have a palisadoderm or physalo-palisadoderm pileipellis, as is the case in the genus *Xerocomellus*, although *H.arduinus*, *H.campestris*, *H.coccyginus*, *H.flavorubellus*, and *H.napaeus* appear to display a trichoderm pileipellis (Thiers and Smith 1966; Smith and Thiers 1971; Frank et al. 2020; Xie et al. 2020). According to literature and our current observations, the hymenophoral trama of *Hortiboletus* species is intermediate between the “*Boletus*-type” and the “*Phylloporus*-type” and an intermediate structure is also typical of stipitate-pileate *Xerocomellus* species (Šutara 2008; Vizzini 2015). When compressed under a cover glass, the lateral strata separate easily, as in the “*Boletus*-type” trama (Singer 1965). In the descriptions of *H.arduinus*, *H.napaeus*, *H.rufosquamosus*, *H.rupicapreus* and *H.tomentosus*, the trama is reported to be of the boletoid type (Xie et al. 2020; Lebeuf et al. 2021; Mao et al. 2023). However, we assume that their trama is also intermediate, although the authors of these species likely did not recognise this transitional type.

Species of this genus develop ectomycorrhizal associations with plants of the families *Betulaceae*, *Cistaceae*, *Corylaceae*, *Fagaceae*, *Malvaceae*, *Pinaceae*, and *Salicaceae*, growing preferably in grassy forests, forest edges, meadows, riverbanks, and disturbed places in general, such as urban or rural areas (urban parks, public and private gardens, lawns, flowerbeds, ruderal places, pastureland, open heaths, abandoned agricultural fields, sport fields, parking lots, roadsides, clearings, drainage ditches, etc.) (Ladurner and Simonini 2003; Gelardi 2009; Das et al. 2016; Wu et al. 2016; Naseer et al. 2019; Frank et al. 2020; Lebeuf et al. 2021). *Hortiboletus* species are edible, but similarly to other xerocomoid boletes, not particularly palatable. Accordingly, they do not have economic importance and are rarely collected for consumption by mushroom hunters.

**Figure 4.**
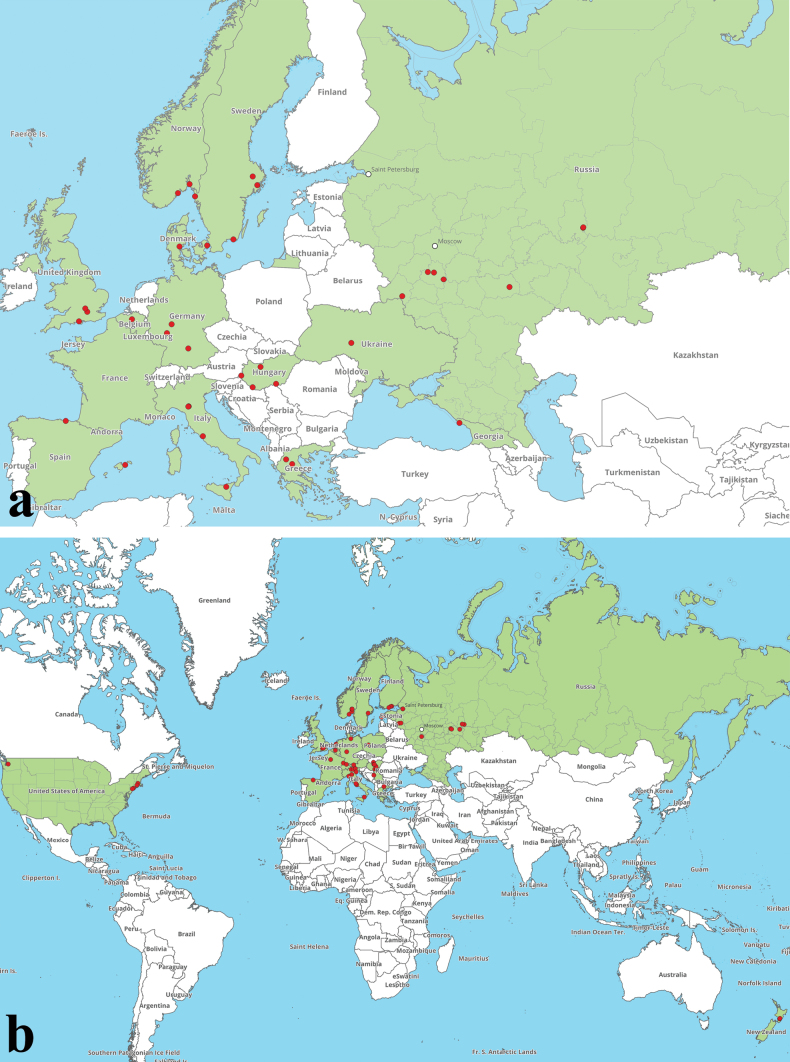
Distribution map of **a***H.rubellus* and **b***H.bubalinus*, based on the current study and sequence data from public repositories and associated publications.

**Figure 5.**
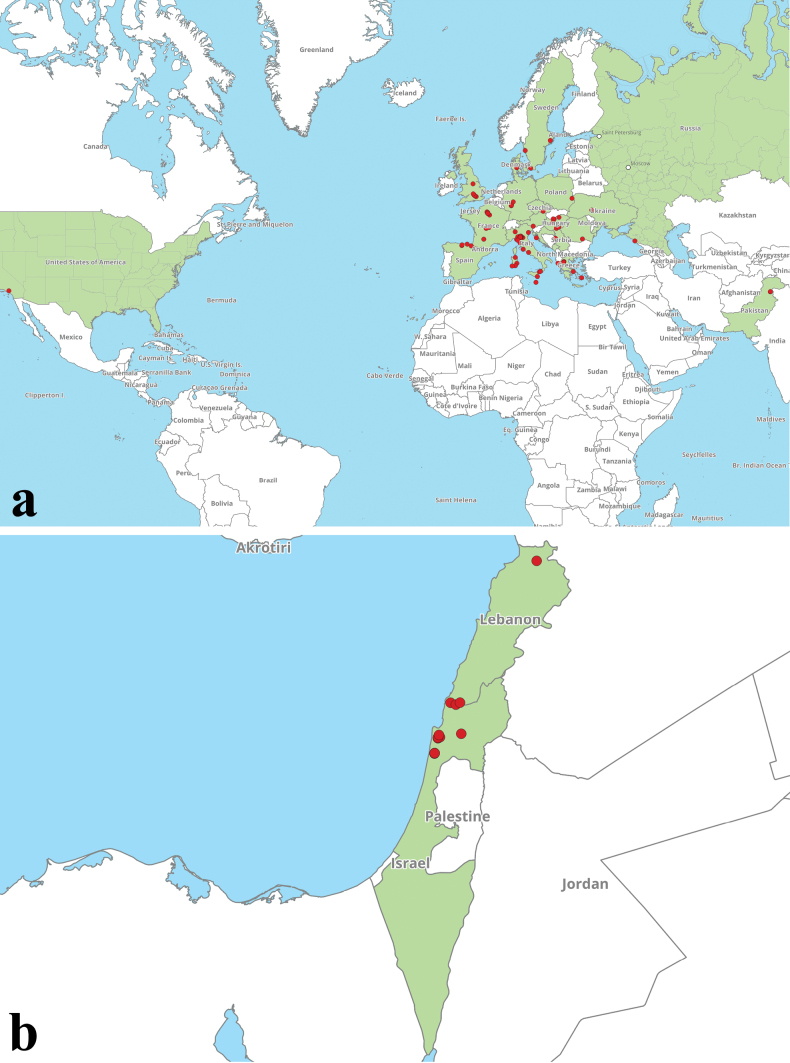
Distribution map of **a***H.engelii* and **b***H.hershenzoniae*, based on the current study and sequence data from public repositories and associated publications.

Maps of distribution of four target species (*H.rubellus*, *H.bubalinus*, *H.engelii*, and *H.hershenzoniae*), based on the current study and sequence data from public repositories and associated publications, are given below (Figs 4, 5).

### ﻿ Hortiboletus rubellus

Taxon classificationFungiBoletalesBoletaceae

(Krombh.) Simonini, Vizzini & Gelardi, in Vizzini, Index Fungorum 244: 1 (2015)

FFE19591-C8E3-5D93-92FE-F8A460BD443B

MB551214

Figs 3a , 6 , 7 , 8

≡ Boletusrubellus Krombh., Naturgetr. Abbild. Beschr. Schwämme 5: 12 (1836)
≡ Suillusrubellus (Krombh.) Kuntze, Revisio generum plantarum 3(3): 536 (1898)
≡ Leucobolitesrubellus (Krombh.) Beck, Z. Pilzk. 2(7): 142 (1923)
≡ Tubiporusrubellus (Krombh.) S. Imai, Trans. Mycol. Soc. Japan 8(3): 113 (1968)
≡ Xerocomellusrubellus (Krombh.) Šutara, Czech Mycol. 60(1): 50 (2008)
= Xerocomusrubellus Quél., Compt. Rend. Assoc. Franç. Avancem. Sci. 24(2): 620 (1896) [1895]
= Boletusversicolor Rostk., in Sturm, Deutschl. Fl., 3 Abt. (Pilze Deutschl.) 5: 55 (1844), *nom. illegit.*, Art. 53.1 (Shenzhen), non Boletusversicolor L., Sp. Pl. 2: 1176 (1753) = Trametesversicolor (L.) Lloyd (1921) [1920]
= Versipellisversicolor (Rostk.) Quél., Enchir. Fung.: 158 (1886), *nom. illegit.*, Art. 53.1 (Shenzhen)
= Versipellispruinatavar.versicolor Quél., Enchir. Fung.: 158 (1886), replaced synonym of Boletusversicolor Rostk. (1844)
? = BoletussanguineusWith., Syst. Arr. Brit. Pl., Edn 4 4: 313 (1801), *nom. illegit.*, Art. 53.1 (Shenzhen), non Boletussanguineus L., Sp. Pl., Edn 2 2(2): 1646 (1763) = Pycnoporussanguineus (L.) Murrill (1904)
? = Viscipellissanguinea (With.) Quél., Enchir. Fung. (Paris): 156 (1886), *nom. illegit.*, Art. 53.1 (Shenzhen)
? = BoletusrutilusFr., in Fries & Hök, Boleti, Fungorum generis, illustratio: 5 (1835)

#### Holotype.

CZECHIA • tab. XXXVI, figs 21–24 (Krombholz 1836).

**Figure 6.**
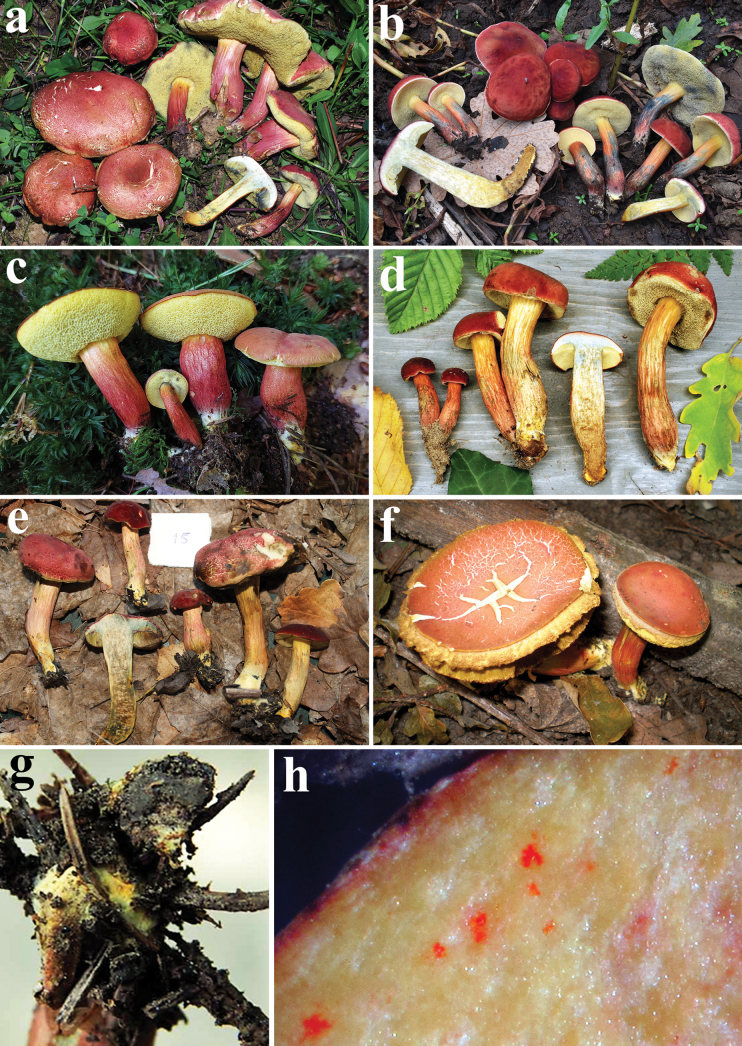
*Hortiboletusrubellus* basidiomes: **a** epitype collection MCVE31743; **b** MG781; **c** DB-2022-06-29-3; **d** AL 13-125, **e** LE F-316026; **f** K-M001435677; **g** basal mycelium of Vgy-2018-07-21-1; **h** orange-red dots in the stipe base context of GK6705. Photos: (**a**) G. Simonini; (**b**) M. Gelardi; (**c**) B. Dima; (**d**) L. Albert; (**e**) A. I. Ivanov; (**f**) A. Yu. Biketova; (**g**) G. Vrba; (**h**) G. Konstantinidis.

#### Epitype designated here

**(MBT 10022257).** ITALY • Emilia-Romagna: Reggio Emilia, Viano, Pulpiano, 44°30'23"N, 10°33'24"E, 507 m, several young to mature basidiomes growing in a cultivated field beside a track, close to a *Quercuscerris* and *Q.pubescens* wood, 19.06.2011, leg. & det. G. Simonini, MCVE31743 (GS10212 – collector’s number, K-M001435705 – isoepitype), GenBank: ITS – PV094851.

**Figure 7.**
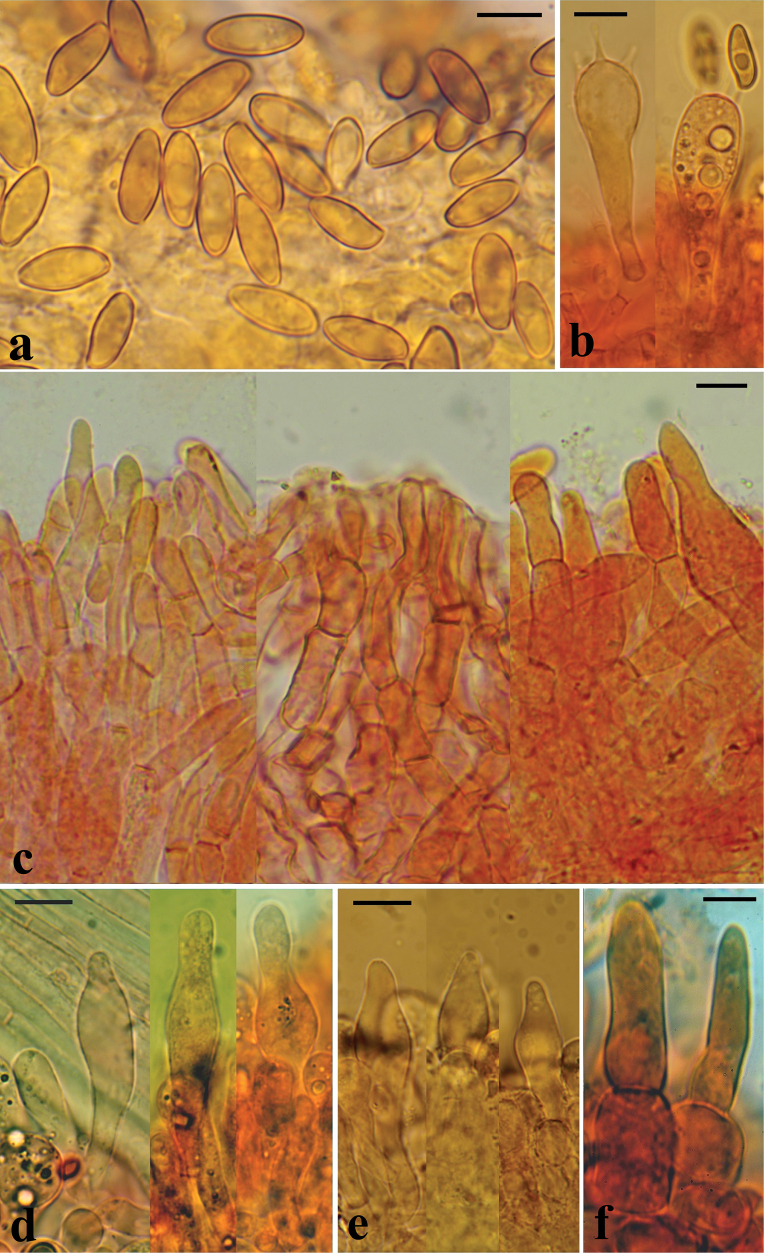
Microscopical features of *H.rubellus*: **a** basidiospores of the epitype collection MCVE31743 in L4; **b** basidia in Congo red, left collection K-M001437348, right – MCVE31743; **c** pileipellis in Congo red, left collection MCVE31743, centre – GS10226, right – GS11020; **d** pleurocystidia in Congo red (K-M001437348); **e** cheilocystidia in Congo red (GS11020); **f** end cells of pileipellis in Congo red (GS10226). Scale bar: 10 μm. Photos: G. Simonini.

#### Description.

***Basidiomes*** pileate-stipitate, xerocomoid, epigeal, small to medium-small sized. ***Ontogenetic development*** gymnocarpic.

**Figure 8.**
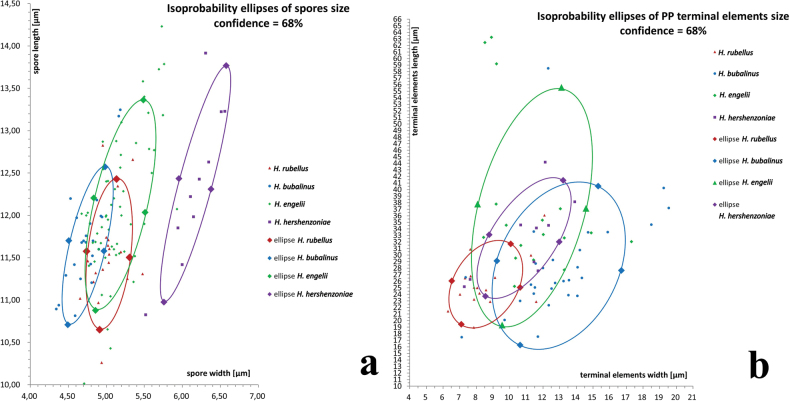
Size distributions of **a** spores and **b** terminal cells of pileipellis in *Hortiboletus* using the “isoprobability ellipse” method. The distributions of average spore sizes in collections are shown with one standard deviation.

***Pileus*** (2–)3.5–10(–15) cm broad, nearly hemispherical at first, then convex, pulvinate to applanate, also slightly depressed at centre, sometimes irregularly shaped; surface matt, dry, ± finely tomentose to sometimes rugulose, but later smooth, sometimes areolate towards the peripheral zone or with minute to coarse cracks especially in dry weather and showing a yellowish or somewhat pinkish subcuticular layer resembling some areolate *Xerocomellus* species; margin initially involute then extended, regular to wavy-lobed, acute and slightly exceeding the pileal context; rather variable in colours, but generally with bright red tints, initially blood red, wine red, cherry red, rose red, orange-red, pink, fading in mature basidiomes from ochraceous red, ochraceous pink to reddish-brown, with a margin at the very beginning whitish (due to a narrow band 1–2 mm broad), then gradually concolourous with age, unchanging or becoming slightly darker on handling or when injured.

***Tubes*** up to 12 mm long, at first adnate, later emarginate or slightly decurrent with a tooth, initially lemon or chrome yellow, later greenish-yellow to olive brown, slightly bluing when injured.

***Pores*** large, wider than 1 mm (up to 1.5–2 mm) in old basidiomes, roundish to angular, concolourous with the tubes, in mature basidiomes with orange-rusty tinges, weakly bluing when bruised and later becoming dirty brownish.

***Stipe*** (2–)2.5–7(–12) × 0.4–2.5(–3.5) cm, usually as long as or slightly longer than pileus diameter, central or slightly off-centre, solid, firstly versiform, clavate or somewhat bulbous, later more cylindrical or sometime inflated in the lower part, always tapering at the very base and slightly rooting; surface with fine reddish punctuations or fibrils on an ochraceous yellow background, later often with rather coarse longitudinal striations; surface usually concolourous with the pileus or paler, but yellow or pale yellow at apex; sometimes the stipe can be entirely yellow or entirely red, or a mix of both colours; basal mycelium whitish-yellow.

***Context*** rather firm when young, later soft, becoming fibrous in the stipe (up to 1.5 cm in the centre of the pileus); pale yellow, dirty ochraceous yellow in the lower half of the stipe and eventually pale pinkish underneath the cuticle; in the stipe base with a typical orange-red pigment in the form of small dots, which are sometimes scarce and hardly noticeable or conversely abundant and then visible as a large flame-orange area at the stipe base; light blue on exposure, especially in the pileus-stipe connection zone, above the tubes and in the peripheral layers of the stipe, later fading to dirty yellow; discolouration less pronounced in dry and old basidiomes.

***Spore print*** olive brown.

***Odour*** pleasantly mushroom-like to fruity or inconspicuous. ***Taste*** mild.

***Macrochemical spot-test reactions***: Melzer’s reagent: a weak fleeting-amyloid reaction in the stipe context and exceptionally also in the hymenophoral trama of dried basidiomes sometimes occurs, but most collections show a strong dextrinoid reaction in all tissues; 20% KOH: reddish-brown on pileus and hymenophore, pinkish on pileal context, bright orange on stipe surface and context and progressively darker downwards; 25% NH_4_OH: negative to pale yellow everywhere; 10% FeSO_4_: sordid green everywhere, particularly on hymenophore.

***Basidiospores*** [561/19/19] (10.3–)11.6 ± 0.6(–12.8) × (4.7–)5.0 ± 0.2(–5.5) μm, Q = (2.07–) 2.31 ± 0.12(–2.59), Vm = 155 ± 17 μm^3^, inequilateral, broadly ellipsoid or fusiform to ellipsoid-fusiform in side view, ellipsoid to amygdaliform or slightly fusiform in face view, smooth under both light microscope and SEM, apex rounded, with a short apiculus, usually with a weak to pronounced suprahilar depression and with a slightly pronounced adaxial swelling, moderately thick-walled (up to 1 μm), straw yellow to honey yellow in water and 5% KOH, having one or two large oil droplets when mature, rarely pluri-guttulate, inamyloid and orthochromatic.

***Basidia*** [17/4/4] (24.8–)33.9 ± 6.1(–46.3) × (7.8–)10.8 ± 1.3(–12.0) μm, subclavate to clavate, predominantly 4-spored, sterigmata 3.1–5.0 μm, hyaline to yellowish in 5% KOH, and containing concolourous oil guttules; basidioles subcylindrical to faintly clavate, similar in size to basidia.

***Cheilocystidia*** [17/4/4] (26.0–)33.8 ± 7.0(–43.8) × (8.7–)9.3 ± 0.4(–10.0) μm, relatively common, scattered, slender, ventricose-fusiform, often with elongated straight or flexuous neck, with rounded tip, smooth, hyaline to pale yellowish in water and 5% KOH, without epiparietal encrustations. ***Pleurocystidia*** [10/2/2] (21.9–)37.8 ± 10.2(–51.3) × (6.0–)8.4 ± 0.7(–11.3) μm, infrequent, slender, ventricose-fusiform, often with elongate neck, mostly hyaline, rarely with yellowish content in 5% KOH. ***Pseudocystidia*** not observed.

***Hymenophoral trama*** bilaterally divergent, intermediate between the “*Phylloporus*-type” and the “*Boletus*-type”: lateral strata consisting of poorly divergent, tightly arranged, non-gelatinous hyphae almost touching each other, hyaline to very pale yellow in water and 5% KOH; mediostratum consisting of tightly adpressed, non gelatinous, parallel running hyphae; in Congo red the mediostratum is slightly darker than the lateral strata.

***Pileipellis*** a physalo-palisadoderm consisting of moderately long, more or less cylindrical, septate hyphae; ***terminal elements*** [539/15/15] (21.4–)25.6 ± 4.2(–36.1) × (6.3–)8.6 ± 1.7(–12.1) μm, Q = (2.03–)3.12 ± 0.55(–4.19), versiform, mainly broadly cylindrical with rounded apex to cystidioid, sometimes also with elongate neck, but also slender, cylindrical, moderately clavate, slightly tapering towards the tip, lageniform, utriform, acorn-shaped, sometimes bifurcate, apex mainly rounded-obtuse, but sometimes pointed, not encrusted (especially in young basidiomes) or poorly and finely encrusted (fine granular or rarely zebra-like epiparietal encrustations) at base, hyaline to pale yellow in water and 5% KOH; ***subterminal elements*** subcylindrical or somewhat inflated and then broader than terminal ones (up to 18 μm), sometimes branched, more frequently encrusted, pale yellowish-brown in 5% KOH. ***Subpileipellis elements*** usually distinctly encrusted by a fine, pale yellow, granular pigment (5% KOH).

***Stipitipellis*** consisting of slender, subparallel to loosely interwoven, smooth-walled, hyaline to pale yellow hyphae 3–5 μm broad, forming a partially developed ***caulohymenium***; in the upper third of the stipe, bundles of caulohymenium consist of clavate, hyaline to yellow caulobasidia (30–50 × 8–15 μm), caulobasidioles, and broadly ventricose-fusiform or clavate caulocystidia (24–50 × 7–11 μm), with an often extremely elongated neck (to 30 μm long). ***Stipe texture*** formed of parallel running, ± hyaline, 2–4 mm broad hyphae; scattered to scarce orange-red crystals (hyphal excretions) dissolving in 5% KOH found in the stipe base context. ***Lateral stipe stratum*** under the caulohymenium poorly differentiated from the stipe trama, consisting of divergent, inclined and running towards the external surface, densely arranged hyphae.

***Stipe trama*** inconspicuous, formed of parallel running, filamentous, 4–13 μm broad hyphae, ± hyaline in water and 5% KOH, inamyloid.

***Clamp connections*** absent in all tissues.

#### Ecology and phenology.

Solitary, gregarious in small groups (2–3 basidiomes) or even subcaespitose, growing on bare soil, on litter or in the grass, often in anthropogenic environments, such as gardens, urban parks, lawns, meadows, disturbed roadsides, light grassy forest and planting edges, shrublands, sometimes in undisturbed forests, mostly under broadleaved trees including preferably *Quercus* spp. (*Q.cerris*, *Q.coccifera*, *Q.faginea*, *Q.frainetto*, *Q.petraea*, *Q.pubescens*, *Q.pyrenaica*, *Q.robur*), *Castaneasativa*, *Fagus* spp. (*F.sylvatica*, *F.orientalis*), and *Tilia* spp. (*T.cordata*, T.×europaea). It was also collected in mixed forest with the presence of *Carpinusbetulus*, *Corylusavellana*, *Betulapendula*, and sometimes *Pinussylvestris* and *Salix* spp., but there are no reports in monodominant forests consisting of these species. There are some molecularly unconfirmed data (Engel et al. 1996; Ladurner and Simonini 2003; Assyov in litt.; Klofac in litt.; pers. obs.) about a possible ectomycorrhizal association of *H.rubellus* with *Abies*, *Alnus*, *Cedrus*, *Picea*, *Populus*; however, they are not confirmed in the present study. The main fruiting period is spring (late April) to autumn (November to early December).

#### Known distribution (see Fig. 4a).

Proven area of this species distribution is Europe and Western Asia (Fig. 4a). Asia: Abkhazia* (this study, GP), Armenia (Nanagulyan 2008), Azerbaijan (Mustafabayli and Aghayeva 2019), and Turkey (Sesli and Denchev 2009); Europe: Austria (Breitenbach and Kränzlin 1991; Ladurner and Simonini 2003), Belgium (Vadthanarat et al. 2019, GP), Bulgaria (Assyov and Denchev 2004, 2010), Croatia (Tkalčec and Mešić 2003), Czechia (Pilát and Dermek 1974; Šutara et al. 2009; Mikšík 2017), Denmark (Knudsen and Taylor 2008, 2012; Kjøller unpubl., GP), Finland (Ladurner and Simonini 2003; Knudsen and Taylor 2008, 2012), France (Lannoy and Estadès 2001; Ladurner and Simonini 2003; Eyssartier and Roux 2011; this study), Germany (Engel et al. 1996; Ladurner and Simonini 2003; this study, GP), Greece (Zervakis et al. 1998; Konstantinidis 2014; this study, GP), Hungary (Dima et al. 2013, Kaposvári 2013; this study, GP), Italy (Alessio 1985; Simonini 1998a, 1998b; Lunghini and Perrone 2001; Papetti et al. 2001; Taylor et al. 2001; Cazzoli 2002; Ladurner and Simonini 2003; Boccardo et al. 2008; Galli 2013; Vasquez 2014; this study, GP), Malta (Mifsud 2017; Sammut 2021), Montenegro (Perič and Perič 2006; Kasom and Karadelev 2012), Netherlands (Ladurner and Simonini 2003; Noordeloos 2007), North Macedonia (Karadelev et al. 2007), Norway (Knudsen and Taylor 2008, 2012, this study, GP), Poland (Łuszczyński 2007), Portugal (Calzada Domínguez 2007), Romania (Pál-Fám and Benedek 2010), Russia (Bolshakov et al. 2021; this study, GP), Serbia (Uzelac 2009; Atlagić et al. 2013; Petrović et al. 2019), Slovakia (Vasas and Locsmándi 2013), Spain (Ladurner and Simonini 2003; Pardo et al. 2004; Calzada Domínguez 2007; Muñoz et al. 2008; this study, GP), Sweden (Taylor and Eberhardt 2006; Knudsen and Taylor 2008, 2012; this study, GP), Switzerland (Breitenbach and Kränzlin 1991), Turkey (East Thrace) (Asan and Gücin 1990), Ukraine (Zerova and Rozhenko 1988; Akulov and Prydiuk 2007; this study, GP), and United Kingdom (Watling and Hills 2005; Hills 2008; Kibby 2017; this study, GP). This species is also recorded in North Africa (Algeria, Morocco, Tunisia) (Singer 1967; Malençon and Bertault 1970; Bertault 1979; Haimed et al. 2013; Nounsi et al. 2014; Outcoumit et al. 2014; El Mokni and El Aouni 2019; Ouali et al. 2021; Youcef Khodja et al. 2021), Macaronesia (Canary islands) (Bañares Baudet 1988), East Asia (Russian Siberia, China, Japan, Thailand) (Chiu 1957; Singer 1967; Vasiljeva 1978; Bi et al. 1994; Engel et al. 1996; Nagasawa 1997; Zhuang 2001; Zang 2006; Mao 2009; Bolshakov et al. 2021; Thongkantha et al. 2021); and North America (Guatemala, Mexico, USA) (Singer 1945, 1947, 1967; Smith and Thiers 1971; Grund and Harrison 1976; Both 1993; Bessette et al. 2000, 2016, 2019; García-Jiménez 2013; Flores Arzù 2020; Saldivar 2021). However, extra-European and extra-Western Asian records, perhaps except for north African samples, may represent different *Hortiboletus* species and their identity should be verified by DNA sequencing. Based on the description and illustration (p. 101; Pl. 12, Fig. 2) of Binyamini and Avizohar-Hershenzon (1973), a record of this species (as *Xerocomusrubellus*) from Israel represents *Xerocomellusredeuilhii* A.F.S. Taylor, U. Eberh., Simonini, Gelardi & Vizzini.

#### Additional examined material.

ABKHAZIA* • Gagra District: near Pitsunda, vicinity of Ptitsefabrica, on a bank of Bzyb River near the mouth, 43°11'53.5"N, 40°18'49.0"E, on soil near a path in mixed *Quercus-Fagus* forest, 02.10.2007, leg. A. Kiyashko, det. A. Kiyashko & T. Svetasheva, LE F-265201 (GP); FRANCE • Île-de-France: Paris, Thoiry, 48°51'48"N, 10°31'09"E, 165 m, five basidiomes in the grass, under *Quercus* sp., 30.07.1993, leg. & det. G. Redeuilh, GS1116; GERMANY • Hesse: Giessen, old graveyard, under *Quercus* sp., 10.06.1998, leg. & det. A. Taylor, AT1998115 (GP); Rhineland-Palatinate: Mainz, Ober Olmer Wald, mixed woodland, but mainly *Quercus* sp., 05.08.1998, leg. & det. A. Taylor, AT1998046 (GP); GREECE • Central Greece: Trikala, Logga, 39°48'60"N, 21°55'54"E, under *F.sylvatica*, 02.07.2012, leg. G. Konstantinidis, det. G. Konstantinidis, B. Dima & A. Biketova, GK6074 (GP); • West Macedonia: Kozani, Vouchorina, 40°12'34"N, 21°15'59"E, under *Quercus* sp., 03.07.2012, leg. G. Konstantinidis, det. G. Konstantinidis, E. Polemis & A. Biketova, GK6705 (GP); HUNGARY • Csongrád-Csanád: Szeged, Elisabeth Park, 46°14'43.9"N, 20°09'49.6"E, on the ground in the grass, with various deciduous trees in vicinity, five basidiomes, 19.07.2020, leg. A. Biketova & B. Dobrić, det. A. Biketova, K-M001435676 (AB B20-358; GP); • Somogy: Darány, under *Q.robur*, *C.betulus*, *Tilia* sp., 18.09.2013, leg. & det. L. Albert, AL 13-125 (GP); • Vas: Őrség NP, Szalafő, on a forest path in mixed forest dominated by *Q.petraea*, *F.sylvatica*, *C.betulus*, *P.sylvestris*, 29.06.2022, leg. & det. D. Varga & B. Dima, DB-2022-06-29-3 (GP); • Komárom-Esztergom: Vértes Mts, Csákányospuszta, Mária-szakadék, in mixed forest with *Quercus* sp., *F.sylvatica*, *C.betulus*, 21.07.2018, leg. & det. Gy. Vrba, VGy-2018-07-21-1 (GP); ITALY • Emilia-Romagna: Reggio nell’Emilia, Carpineti, Pantano, 44°29'23"N, 10°31'09"E, 670 m, a single basidiome at the edge of the wood, with *Quercuspubescens* and *C.sativa*, 23.10.1993, leg. & det. G. Simonini, GS1037; • Reggio nell’Emilia, Viano, Pulpiano, 44°30'35"N, 10°33'22"E, 515 m, nine basidiomes in a field cultivated with *Medicagosativa*, close to *Q.cerris* and *Q.pubescens* wood, 03.07.1994, leg. & det. G. Simonini, MCVE17716 (GS1180); • ibid., 44°30'33"N, 10°33'20"E, 512 m, four basidiomes in a field cultivated with *M.sativa*, close to a *Q.cerris* and *Q.pubescens* wood, 10.07.1994, leg. & det. G. Simonini, MCVE17717 (GS1196); • ibid., 44°30'31"N, 10°33'25"E, 526 m, two basidiomes in the path in the furrow of a tractor wheel, in *Q.cerris* and *Q.pubescens* wood, 23.07.1994, leg. & det. G. Simonini, GS1206; • ibid., 44°30'30"N, 10°33'15"E, 507 m, five basidiomes in a field cultivated with *M.sativa*, close to a *Q.cerris* and *Q.pubescens* wood, 30.07.1994, leg. & det. G. Simonini, MCVE17773 (GS1241); • ibid., 44°30'37"N, 10°33'21"E, 517 m, several young to mature basidiomes growing on a lawn near a track, close to a *Q.cerris* and *Q.pubescens* wood, 31.08.1994, leg. & det. G. Simonini, MCVE17903 (GS1424; GP); • ibid., 44°30'32"N, 10°33'20"E, 511 m, several young to mature basidiomes growing in a ploughed meadow, close to a *Q.cerris* wood, 07.06.1998, leg. & det. G. Simonini, MCVE18231 (GS1894; GP); • ibid., 44°30'35"N, 10°33'24"E, 516 m, 9 basidiomes in a cultivated field close to *Q.cerris* and *Q.pubescens* wood, 23.08.2015, leg. M. Comuzzi, det. G. Simonini, GS10226; • Lazio: Manziana (RM), Macchiagrande di Manziana, 42°07'24"N, 12°07'19"E, 320 m, several young to mature basidiomes collected on acidic soil in a very moist grassy clearing bordering a mixed broadleaved woodland dominated by *Q.cerris* with the presence of *Q.frainetto*, *Acer* sp. and *Crataegus* sp., 12.10.2019, leg. M. Gelardi & F. Costanzo, det. M. Gelardi, F. Costanzo & A. Biketova, MG781 (GP); • Sicily: Messina, Cesarò, Portella dei Bufali, 37°52'17"N, 14°41'17"E, 1196 m, five basidiomes in the grass close to *Q.cerris* and *Quercus* sp. trees, 21.10.2013, leg. G. Vasquez, det. leg. G. Vasquez & G. Simonini, K-M001437348 (GS10116); NORWAY • Ostfold Co.: Rygge, Fuglevikasen 12, 59°23'17.8"N, 10°39'29.8"E, 10.09.2014, leg. E.W. Hanssen & R. Braathen, det. B. Dima, O-F-76041 (GP); • Telemark Co.: Kragerø, Berg Museum, 58°53'02"N, 9°22'59"E, 06.08.2000, leg. T.E. Brandrud, det. B. Dima, O-F-168828 (GP); RUSSIA • Bryansk Oblast: Bryansky Forest Reserve, on soil in broadleaf forest dominated mostly by *Q.robur*, *T.cordata*, and *Ulmusglabra*, 07.08.2015, leg. & det. T. Svetasheva, LE F-332230; • Penza Oblast: Gorodishchensky District, vicinity of Nikonovo, floodplain *Quercus* forest, 30.07.2018, leg. A. Ivanov, det. A. Ivanov & T. Svetasheva, LE F-316026; • Tula Oblast: Shchekinsky District, Museum-estate of Lev Tolstoy “Yasnaya Polyana”, on a grassy edge in mixed *Quercus-Tilia* forest, 14.08.2002, leg. & det. T. Svetasheva, LE F-234797; • vicinity of Lesnoy township, “Tulskiye zaseki” broadleaved forest, on soil with *Q.robur*, *T.cordata*, *Acerplatanoides*, *U.glabra*, and *C.avellana*, 27.06.2012, leg. & det. T. Svetasheva, LE F-332231; • Kurkinsky District, vicinity of Khvorostyanka, “Vodyanoe pole” protected area, on soil in broadleaved forest with *Q.robur*, *T.cordata*, and *B.pendula*, 09.07.2001, leg. & det. T. Svetasheva, LE F-315853; • ibid., margin of a broadleaved forest, on soil in the grass, 17.07.2001, leg. & det. T. Svetasheva, LE F-332082; • Udmurt Republic: Votkinskji District, 4.6 km south of Perevoznoe, 56°51'36"N, 53°54'07"E, 75 m, on the ground in a *Q.robur* forest, with *Salix*, 09.07.2015, leg. V. Kapitonov, det. V. Kapitonov & G. Simonini, GS10512 (GP); • Votkinskji district, Perevoznoe, 56°53'13"N, 53°55'31"E, 98 m, on the ground in a *Q.robur* forest, 13.07.2015, leg. V. Kapitonov, det. V. Kapitonov & G. Simonini, GS11020; SPAIN • Bizkaia: Urduliz, Mendiondo, 43°21'23"N, 2°57'00"W, 100 m, under *Q.robur*, 03.09.2005, leg. E. Fidalgo & J. Muñoz, det. J. Muñoz & A. Taylor, JAM0556 (GP); • Burgos: Valle de Mena, Barrasa, 43°06'16"N, 3°19'39"W, 430 m, locality of the holotype of *Xerocomuserubescens*, growing among the grass in a cleared area, under *Q.faginea*, 28.10.2001, leg. J. Muñoz, det. J. Muñoz, A. Taylor & A. Biketova, JAM0224 (BAR1997082010; GP); • Segovia: Boca del Asno, on humus soil, with *P.sylvestris* and *Q.pyrenaica*, 13.10.1999, leg. Soc. Micol. Madrid, det. H. Ladurner & A. Biketova, IB19991021 (GP); SWEDEN • Blekinge Co.; *F.sylvatica* and *Quercus* sp. forest, 04.09.2003, leg. A. Ryberg, det. A. Taylor, AT2003134 (GP); Stockholm Co.: Stockholm, 24.07.2001, leg. H.-G. Toresson, det. A. Taylor, AT2001124 (GP); • Uppsala Co.: Nåsten, Fäbodarna, under *Quercus* sp., 30.07.2005, leg. & det. A. Taylor, AT2005022 (GP); • Västra Götaland Co.: Tanum, Greby, mixed forest, 06.09.2004, leg. J. Nilsson, det. A. Taylor, AT2004285 (GP); UKRAINE • Vinnytsia Region: Vinnytsia Forest Park, 50 m from the northern shore of Guralnya Lake, along a footpath, two mature basidiomes on ground near a fallen tree branch, in *F.sylvatica* and *Q.robur* forest, 09.08.2012, leg. A. Biketova & A. Zamorotskiy, det. A. Biketova, K-M001435677 (KW 60663F; GP); UNITED KINGDOM • England: Berkshire, Windsor Great Park, Bishops gate area, SU975723, under T.×europaea, 23.08.2005, leg. A. Hills, det. A. Hills & A. Taylor, K-M000167799 (AH2005033; GP); • Hampshire, New Forest Wormstall Wood, SZ350980, amongst grass under *Quercus* sp., 28.07.2004, leg. P. Hills, det. A. Hills & A. Taylor, K-M000168971 (AH2004055; GP).

#### Notes.

This species was first described and illustrated as *Boletusrubellus* by Krombholz (1836). The original description (in German) is here translated in English: “*Reddish bolete. The pileus is plane-convex, slightly depressed in the centre, smooth, matt, red, with yellow, weakly adnexed hymenophore consisting of tubes having uniform length, medium-sized, with yellow, small pores, having uniform diameter and almost round; the stipe is slender, straight or curved, with round cross section, smooth, rarely longitudinally streaked, equal, slightly tapering towards the base, having the same red colour of the pileus on a yellow-brown background. The pileus context is fleshy, soft, pale yellow, reddish in the middle part of the stipe, dark yellow at the base; no odour, no peculiar taste*”.

A short Latin diagnosis follows: “*Boletus* pileo *plano-convexo, medio subdepresso, laevi, glabro, opaco, rubro*; hymenio *luteolo, subadfixo*, tubulis *subaequalibus, mediocribus*, ostiolis *flavidis, minutis, aequalibus, subrotondis*; stipite *longo, erecto flexuoso, tereti, glabro, rarius striato, aequalis, basi fuscescente, flavido*; substantia *carnosa, mollis, pilei pallide flava, stipitis medio et supra rubella, infra interne lutea*. Odore *nullo*, sapore *non speciali*”.

The holotype of Krombholz’ species is an illustration (table 36, figs 21–24) showing four basidiomes, one of them cut in half. The drawings are very schematic, they highlight the bright red pileus and stipe, the stipe tapering at the base and longitudinally streaked, the pale yellow context that is darker and reddish at the stipe base (Krombholz 1836).

Later in 1896, Lucien Quélet described *Xerocomusrubellus*. The diagnosis is here translated from French: “*Stipe thin, velvety and red, pale-yellow-coloured at apex and at the base. Pileus convex (0.02–0.03 m), finely tomentose, bright blood red; context daffodil-like coloured (light yellow), then pink, blue and green, having an abundant green juice; taste fruity, smell sweet then sour. Tubes small, lemon creme; pores sinuate, pubescent, daffodil sulphur yellow, turning green similarly to the stipe. Spores fusoid (0.01–0.011 mm), pale lemon. (Pl. VI, fig. 11.)*.

*Summer.* – *embankments and roads in grassy woods, Nivernais (Mme Daulnoy), Maritime Alps (Barla); it is close to* versicolor. *The species having the same name by Krombholz is the same as* sanguineus *With. and*rutilus*Fr*”.

Although the matter is debatable, we here traditionally assume that the original material of Quélet’s *X.rubellus* is the drawing (Quélet (1896), T. XXIV Pl. VI fig. 11), constituted by a single basidiome. Quélet’s drawing is difficult to interpret, the only emerging character being the tapering stipe base.

The combination “*Xerocomusrubellus* (Krombh.) Quél. 1896”, sometimes still used, does not comply with Art. 41 (ICN Shenzhen, Turland et al. (2018)) about the validity of new combinations, since no reference to a basionym is provided by Quélet. On the contrary, Quélet (1896: 620) wrote “*L’espèce de mème nom de Krombholz est le mème que* sanguineus, *With. et*rutilus, *Fr.*” (“*The species with the same name by Krombholz is the same as* sanguineus *With. and*rutilus*Fr.*”). Therefore, Quélet assumed that *Boletusrubellus* Krombh. is a synonym of both *B.sanguineus* With. and *B.rutilus* Fr., excluding “his” *X.rubellus*. Quélet’s standpoint was also expressed in his previous work Enchiridion Fungorum (1886), where he formally assessed the synonyms of *B.rubellus* Krombh., namely *B.sanguineus* With. and *B.rutilus* Fr., and accommodated them in the genus *Viscipellis*, while the dry-pileus boletes were assigned to the genus *Versipellis*. According to Quélet, *B.rubellus* Krombh. is definitely different from *X.rubellus* (dry velvety pileus) and the latter is a valid name for a new species, having a different type from that of *B.rubellus* Krombh. The citation “*Xerocomusrubellus* (Krombh.) Quél. 1896” is a mistake introduced by some later authors and then multiplied and has, therefore, to be rejected (Redeuilh 1990).

However, by reading the original diagnosis of *B.rubellus* and *X.rubellus* and by looking at their respective illustrations, we observe no major differences between them. They both have a red pileus and stipe (the latter with a yellowish tint at the apex and at the base) and a matte, tomentose to velvety pileus surface. Assuming that the identities of *B.rubellus* and *X.rubellus* are overlapping, Krombholz’ name would have priority, being the older of the two.

It is difficult to understand why Quélet interpreted *B.rubellus* Krombh. as a bolete with a viscid pileus. Some specimens collected in rainy conditions may effectively show a slight degree of gelatinisation or perhaps he confused them with *Aureoboletusgentilis*, placing it in “*Viscipellis*” and in synonymy with other boletes with a viscid pileus. Recently, *B.rubellus* Krombh. was assigned to *Hortiboletus* and designated as the type of the newly-established genus (Vizzini 2015). Furthermore, we herein designate an epitype of *Boletusrubellus* complying with Krombholz’s description and holotype.

We sequenced the ITS region of *Hortiboletus* specimen JAM0224 (BAR1997082010) growing in the type locality of *Xerocomuserubescens* Cadiñanos & Muñoz, which turned out to be conspecific with *H.rubellus*. *Xerocomuserubescens* has the pinkish tint in the pileus context (Cadiñanos and Muñoz 1992), which is occasionally present in *H.rubellus*. It is worth noting, however, that *X.erubescens* superficially resembles *H.engelii* due to the lack of reddish tints on both pileus and stipe surfaces (except for very young specimens), whereas the pinkish tinge of the upper pileus context and the pileipellis structure (Cadiñanos and Muñoz 1992) are much more reminiscent of the typical diagnostic features of *H.bubalinus* (Oolbekkink 1991). In addition, the typical orange-red pigment in the stipe base of *H.rubellus* and *H.engelii* is not described for *X.erubescens*, but this character is also often overlooked (Ladurner and Simonini 2003). Therefore, due to this and to the fact that at least two species of *Hortiboletus* can grow in the same locality, we cannot treat JAM0224 as a topotypical collection of *X.erubescens*. Unfortunately, multiple attempts to sequence (using Sanger sequencing) the holotype collection of *X.erubescens* have failed.

There are a number of intraspecific taxa of *H.rubellus* (namely twelve according to Index Fungorum (2025)) that have been described from areas far away from the currently known geographic range of *H.rubellus*. However, most of them are likely to represent other species. This is the case with Boletusrubellusvar.flammeus, which is shown to be a synonym of Boletus (Hortiboletus) flavorubellus (see Extralimital taxa).

Based on the present multilocus phylogenetic analysis (Fig. 1), *H.rubellus* belongs to the crown clade of *Hortiboletus* and forms a well-supported terminal clade (BS = 97; PP = 0.97), which is sister to *H.sinorubellus* from China (BS = 100; PP = 1.00). However, this sister relationship was not shown in the *rpb2* analysis (Fig. 2). Using the ITS barcoding region for comparison, the closest European species to *H.rubellus* is *H.engelii* with a remarkable 9% dissimilarity (> 50 nucleotide and indel differences). The ITS of *H.rubellus* has been difficult to sequence with the classical Sanger method due to tandem repeat insertions in ITS: 1) short AC repeats in the ITS1 region that vary in length within the species from 14 to 26 bp and even within the same genome with maximum observed difference of 2 bp (in GS1424 and K-M000167799); and 2) long tandem repeats in the ITS2 estimated to be from 166–167 bp (2-7-7) to 165–170 bp (2-3-5), based on different alignment parameters.

### ﻿ Hortiboletus bubalinus

Taxon classificationFungiBoletalesBoletaceae

(Oolbekk. & Duin) L. Albert & Dima, Index Fungorum 251: 1 (2015)

D6EA9E4B-9B39-5128-9B91-53846A27A41D

MB551312

Figs 8 , 9 , 10

≡ Boletusbubalinus Oolbekk. & Duin, in Oolbekkink, Persoonia **14**(3): 367 (1991)
≡ Xerocomellusbubalinus (Oolbekk. & Duin) Mikšík, Index Fungorum **182**: 1 (2014)
≡ Xerocomusbubalinus (Oolbekk. & Duin) Redeuilh, Docums Mycol. **23**(no. 89): 62 (1993)
? = Xerocomuserubescens Cadiñanos & J.A. Muñoz, Belarra **9**: 61 (1992)
? – ‘Boletuspopulinus’ Oolbekk. & Duin (1988), Coolia **31**(1): 11, *nom. illeg.*, *nom. prov.*, Art. 53.1 (Shenzhen), non B.populinus Schumach., Enum. Pl. **2**: 384 (1803), q. e. Oxyporuspopulinus (Schumach.) Donk

#### Holotype examined here.

NETHERLANDS • North Holland: Aerdenhout, A.W. dunes, near Ezelenvlak, on sandy soil, under *Pinus* and *Populus*, 14.10.1983, leg. G. Oolbekkink & W. van Duin, L0053449 (G. Oolbekkink & W. van Duin 145 – collector’s number), GenBank: ITS – PV094766 and PV094767.

**Figure 9.**
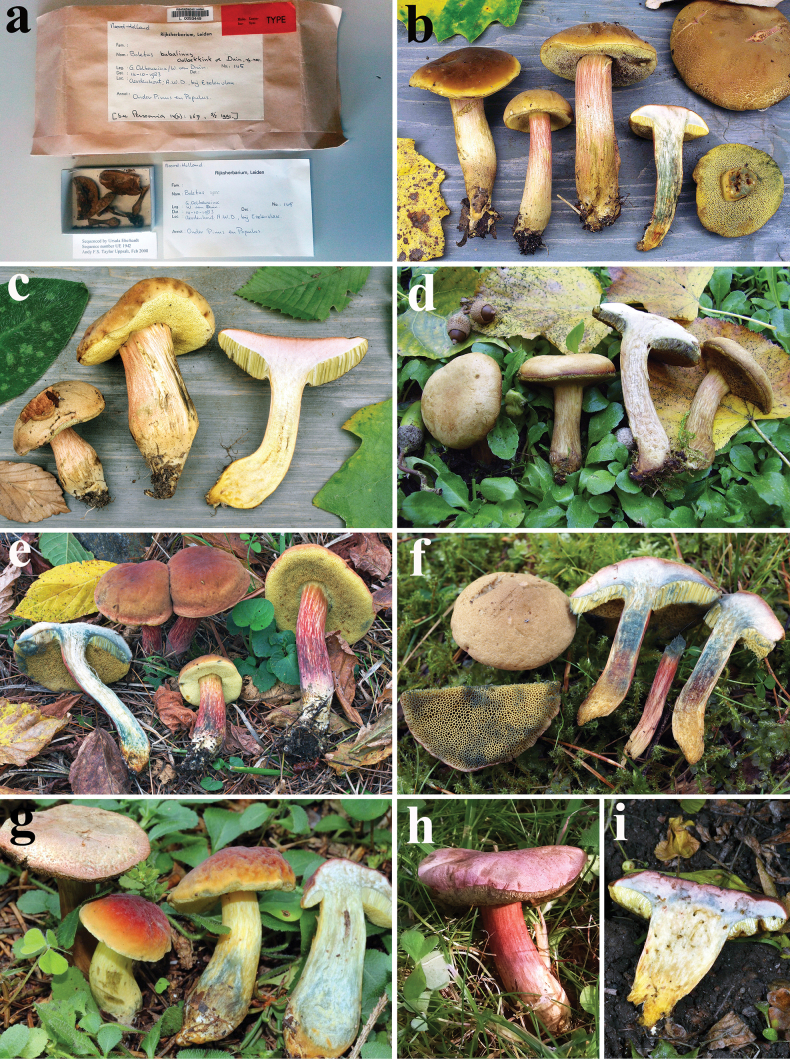
*Hortiboletusbubalinus* basidiomes: **a** holotype collection L 0053449; **b** AL12-28; **c** AL22-75; **d** MCVE25582; **e** TUR-A209256; **f** LE F-315962; **g** GS10842; **h** LE F-332081; **i** LE F-332077. Photos: (**a**) B. Dima; (**b-c**) L. Albert; (**d**) M. Gelardi; (**e**) S. Saitta; (**f**) O. V. Morozova; (**g**) E. Bizio; (**h**) T. Yu. Svetasheva; (**i**) E. S. Popov.

#### Description.

***Basidiomes*** pileate-stipitate, xerocomoid, epigeal, small to medium-small sized. ***Ontogenetic development*** gymnocarpic.

**Figure 10.**
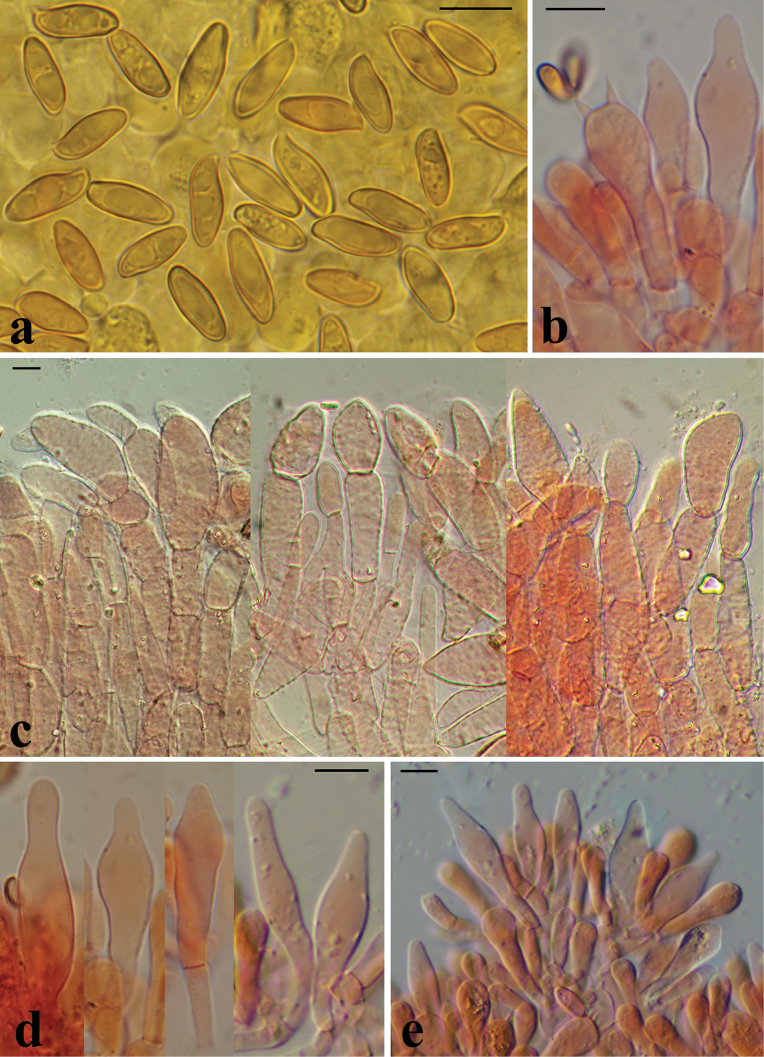
Microscopical features of *H.bubalinus*: **a** basidiospores of collection MCVE18373 in L4; **b** basidia in Congo red (GS10414); **c** pileipellis in Congo red; **d** pleurocystidia in Congo red, left collection GS10072, others – GS10414; **e** tuft of cheilocystidia in Congo red (GS10414). Scale bar: 10 μm. Photos: G. Simonini.

***Pileus*** (2.9–)3.8–10.3(–13.5) cm broad, firstly hemispherical then persistently convex and finally broadly pulvinate-flattened, sometimes slightly depressed at centre, regularly to hardly unevenly shaped by shallow depressions, margin even to faintly wavy-lobed, initially involute then curved downwards or rarely completely plane, not or only a little extending beyond the tubes (up to 1 mm); surface matt, dry, but slightly greasy in aged basidiomes with moist weather, markedly wrinkled and finely pruinose in the early stage of development then tomentose with age, usually not cracked or occasionally areolate towards the peripheral zone; somewhat variable in colour, at first, dark garnet red or dark reddish-brown, but typically paler towards margin (pale orange to yellowish-orange), often later gradually fading to drab tinges and becoming orange ochraceous, sometimes with scattered pinkish hues, then evenly pale ochraceous brown, chamois olive to dull ochraceous buff, often retaining small and dispersed pale-reddish to ochraceous-orange zones (especially in the depressions) and with a thin, reddish-pink marginal band; aged basidiomes can completely lack reddish tones, although some basidiomes retain bright reddish tints with age; unchanging on handling or touching or when injured; subpellis layer pale pink to whitish buff.

***Tubes*** up to 12 mm long, at first, thin in side view then increasingly wider with age and shorter than the thickness of the pileus context, easily separable from the context above, adnate, but soon depressed around the stipe apex and shortly decurrent with a tooth, bright yellow to olive yellow then olive brown, bluing on cutting.

***Pores*** initially forming a concave or ascending surface, later flattening, at first very small then gradually wider with age (up to 2.0 mm in diameter), compound, labyrinthine becoming angular at maturity, concolourous with the tubes; strongly bluing on bruising and later becoming dirty brownish.

***Stipe*** (3.1–)4.3–7.3(–8.3) × 1.1–2.7(–3.2) cm, as long as the pileus diameter at maturity, otherwise slightly to moderately shorter or longer, central to slightly off-centre, solid, firm, dry, straight or faintly curved, at first ventricose or clavate although tapering at the very base, later more or less cylindrical, sometimes remaining inflated downwards or progressively tapering from apex down to the base, not or slightly rooting; surface smooth or with fine punctuations from apex down to the middle part, longitudinally fibrillose with age, without reticulum; usually bright yellow or somewhat reddish at the very apex, flesh pink, reddish-pink to wine red elsewhere, rarely yellow to pale yellow throughout, usually progressively and sometimes completely losing reddish tones with age similarly to the pileus surface and eventually becoming dull buff; yellow ochraceous at the very base, sometimes with brownish-olive hues, occasionally with purplish patches; unchanging or faintly bluing on pressure and finally turning brownish; basal mycelium yellow.

***Context*** firm and tough when young, later soft textured and eventually flabby in the pileus (up to 2.3 cm thick in the central zone, up to 1.5 mm thick halfway to margin and gradually becoming thinner towards the edge), fibrous in the stipe, pinkish-red in the pileus in very young to young basidiomes, but with a thin straw yellow to whitish layer above the tubes, becoming pale flesh pink at maturity and eventually fading to dull white in aged basidiomes or on drying; pale yellowish in the stipe, but progressively duller downwards, dirty yellow ochraceous at the very base and, as a rule, devoid of carrot-orange to orange-red dots, which can rarely be present; quickly bruising from light green-blue to blue on exposure, particularly in the pileus-stipe connection zone, above the tubes and in the peripheral layers of the stipe; after a few minutes, the bluing extends quite intensely to the lower part of the stipe (particularly in young basidiomes) and later becomes drab yellowish-olive, unchanging in the pinkish-red zone underneath the pileus surface; the oxidation phenomenon is less intense in old fruiting bodies and can be absent in very old basidiomes; context pinkish in the areas eaten by slugs and brownish where eroded by maggots; subhymenophoral layer yellowish-white; exsiccate brownish and reddish tones completely disappear during drying process.

***Spore print*** olive brown.

***Odour*** strong, pleasant, fruit-like but with an “iron” component. ***Taste*** mild.

***Macrochemical spot-test reactions***: Melzer’s reagent: a weak fleeting-amyloid reaction on the hymenophoral trama and on the stipe and pileus context of fresh and dry basidiomes; 20% KOH: pale to bright orange on hymenophore and context, reddish-brown on pileus and stipe surface; 25% NH_4_OH: none; 10% FeSO_4_: none; 10% H_2_SO_4_: none on context, orange on pileus and stipe surface and hymenophore.

***Basidiospores*** [1020/35/35] (10.7–)11.7 ± 0.6(–13.3) × (4.3–)4.8 ± 0.2(–5.3) μm, Q = (2.26–) 2.46 ± 0.10(–2.70), Vm = 140 ± 20 μm^3^, sometimes abnormally large – up to 19.0 × 7.5 μm, inequilateral, fusiform to ellipsoid-fusiform in side view, narrowly or moderately ellipsoid to amygdaliform in face view, smooth, apex rounded, with a short apiculus, usually with a distinct suprahilar depression and with a slightly pronounced adaxial swelling, moderately to decidedly thick-walled (up to 1 μm), straw yellow to bright yellow-coloured in water and 5% KOH, having one, two or three large oil droplets when mature, rarely pluri-guttulate, inamyloid and with an orthochromatic reaction.

***Basidia*** [101/7/7] (27.4–)36.1 ± 4.7(–44.2) × (8.8–)11.4 ± 1.4(–13.0) μm, subclavate to clavate, moderately thick-walled (0.5–0.7 μm), predominantly 4-spored, sterigmata (1.5–)3.2 ± 0.8(–4.5) μm, hyaline to pale yellowish and containing straw-yellow oil guttules in water and 5% KOH; basidioles subcylindrical to faintly clavate, similar in size to basidia.

***Cheilocystidia*** [20/3/3] (45.4–)56.7 ± 10.9(–72.3) × (6.9–)8.6 ± 1.8(–11.5) μm, relatively common, moderately slender, projecting straight to sometimes flexuous, subcylindrical-fusiform, fusiform to ventricose-fusiform, with rounded tip, smooth, moderately thick-walled (0.5–1.0 μm), hyaline to pale yellowish in water and 5% KOH, without epiparietal encrustations. ***Pleurocystidia*** [38/3/3] (40.9–)56.6 ± 8.0(–77.9) × (7.9–)11.8 ± 2.3(–16.5) μm, infrequent, shape, size, colour and chemical reactions similar to cheilocystidia. ***Pseudocystidia*** not observed.

***Hymenophoral trama*** bilaterally divergent intermediate between the “*Phylloporus*-type” and the “*Boletus*-type”, but quite closer to the former, with moderately divergent and tightly arranged, non-gelatinous hyphae (lateral strata hyphae in transversal section touching or almost touching each other, 0–4 (–5) μm apart), hyaline to very pale yellowish in water and 5% KOH, inamyloid in Melzer’s; mediostratum axially arranged, consisting of a tightly adpressed, non-gelatinous bundle of hyphae; in Congo red, the mediostratum is concolourous with or slightly darker than the lateral strata.

***Pileipellis*** a physalo-palisadoderm consisting of erect subparallel to parallel chains of short, stout, septate hyphae; terminal elements [1023/29/28] (17.5–)28.7 ± 8.1(–58.5) × (7.1–)13.2 ± 3.1(–20.3) μm, Q = (1.63–) 2.31 ± 0.66(–4.84), versiform, usually more or less swollen, mainly bullet-shaped or acorn-shaped, less frequently broadly cylindrical, ventricose to ovoid or cystidioid, but also globose or subglobose, sometimes mucronate, more rarely peanut-shaped, conical or utriform, occasionally lanceolate or filamentous and sinuous and then particularly long (up to 86 μm long), often bifurcate, apex rounded-obtuse or pointed, moderately thick-walled (up to 1 μm), hyaline to pale yellow in water and 5% KOH; subterminal elements usually inflated, swollen to vesiculose and broader than terminal ones (up to 38 μm broad), often branched; pileipellis elements moderately to strongly ornamented by granular or zebra-like epiparietal encrustations, more evident and pronounced on subpellis hyphae, but also present on terminal cells surface, although less outlined, brownish in 5% KOH.

***Stipitipellis*** a layer of slender, parallel to subparallel and longitudinally running, smooth-walled, adpressed hyphae, 5–14 μm broad, hyaline to pale yellowish in water and 5% KOH; the stipe apex is covered by a well-developed caulohymenial layer consisting of sterile caulobasidioles, very sparse, predominantly 2- and 4-spored, fertile caulobasidia and scattered projecting caulocystidia, similar in shape and size to the hymenial cystidia; elsewhere, the stipe surface exhibits isolated clusters of cystidioid to clavate, sterile elements, 4–9 μm broad. ***Lateral stipe stratum*** under the caulohymenium only rarely present and obscurely differentiated from the stipe trama, of the “boletoid” type; at the stipe apex, a 20–30 μm thick layer consisting of divergent, inclined and running towards the external surface, densely arranged hyphae.

***Stipe trama*** composed of subparallel to confusedly and densely arranged, loosely interwoven, filamentous, smooth, inamyloid hyphae, 2–8 μm broad.

***Clamp connections*** absent in all tissues.

#### Ecology and phenology.

solitary to gregarious or even subcaespitose, growing on litter, among grass or on bare soil, mainly in moist anthropogenic environments, such as public and private gardens, urban parks, or rural areas under a large array of ectomycorrhizal trees of the families *Salicaceae*, *Malvaceae*, *Fagaceae*, *Betulaceae*, *Pinaceae*, including preferably *Populus* spp. (*P.alba*, *P.nigra*, *P.tremula*, *Populus* hybrids), *Tilia* spp. (*T.cordata*, T.×europaea, *T.platyphyllos*), *Quercus* spp. (*Q.cerris*, *Q.ilex*, *Q.petraea*, *Q.robur*), and *Fagussylvatica*, but sometimes also *Picea* spp. (*P.abies*, P.×fennica, *P.pungens*), *Betula* spp. (*B.pendula*), *Carpinusbetulus*, *Corylusavellana*, *Castaneasativa*, *Caryaovata*, and *Pinusradiata*; some collections of *H.bubalinus* have been found in mixed forest with the presence of *Pinus* spp. (*P.nigra*, *P.pinea*, *P.sylvestris*), *Cedrus* (*C.atlantica*, *C.deodara*), *Larixdecidua*, *Salixalba*, and *Alnusglutinosa*, but there are no finds in monodominant forest communities formed by these tree species alone (Oolbekkink (1991); Ladurner and Simonini (2003); Albert (2004); Muñoz et al. (2008); Musumeci (2008); Assyov and Stoykov (2011); Wilgan and Leski (2022), GP; Kasparavičius and Iršėnaitė (2023); Assyov in litt.; Cooper and Park in litt., GP; Klofac in litt.; Saar et al. unpubl., GP; pers. obs., GP). The main fruiting period is from spring (late April) to late autumn and early winter in Mediterranean area (November to early December).

#### Known distribution (see Fig. 4b).

This is a species with a natural circumboreal distribution, widespread in Europe and Western Asia, also found in the USA and introduced in New Zealand (based on sequences data from INSDC, see phylogenetic analysis), and much more frequent than previously assumed (Fig. 4b). Reported to date from Asia: Turkey (Allı et al. 2019); Australia and Oceania: New Zealand (GBIF 2024; Cooper and Park in litt., GP); Europe: Austria (Montag 2009; Šutara et al. 2009; Klofac in litt.; this study, GP), Belgium (Vadthanarat et al. (2019), as *Xerocomellusporosporus*, GP), Bulgaria (Assyov and Stoykov 2011), Czechia (Šuhaj and Mikšík 2016; Mikšík 2017), Estonia (Saar unpubl., GP – UDB024777), Finland (this study, GP), France (Musumeci 2008; Péan and Mornand 2009; this study, GP), Germany (Kaldorf et al. (2004), as Uncultured *Boletaceae*, GP; Klofac (2007); this study, GP), Greece (this study, GP); Hungary (Albert 2004; Kaposvári 2013; this study, GP), Italy (Gelardi 2009, 2010; this study, GP), Kosovo* (Ramshaj et al. 2021), Lithuania (Kasparavičius and Iršėnaitė 2023), Netherlands (Oolbekkink and van Duin 1988; Oolbekkink 1991; Ladurner and Simonini 2003; Noordeloos 2007; Noordeloos et al. 2018; this study, GP), Norway (Knudsen and Taylor 2008, 2012; this study, GP), Poland (Wilgan and Leski (2022), as *Xerocomelluschrysenteron*, GP – ON129125), Portugal (Azores Islands, probably introduced) (Iglesias et al. 2021; Souto et al. 2024), Russia (Kapitonov (2013); Svetasheva et al. (2018); Bolshakov et al. (2021); Pham et al. (2024), GP; this study, GP), Serbia (Uzelac 2009; this study, GP), Slovakia (Mikšík in litt.), Spain (Muñoz et al. 2008; this study, GP), Sweden (Taylor and Eberhardt 2006; Knudsen and Taylor 2008, 2012; Jeppson and Knuttson 2017; this study, GP), Switzerland (this study), United Kingdom (Hills 2008; Burnham and Kibby 2011; Ainsworth et al. 2013; Kibby 2017; this study, GP); North America: USA (Nuhn et al. (2013), as Xerocomelluscf.rubellus, GP; Frank et al. (2020), as *Hortiboletus* sp., GP).

#### Additional examined material.

AUSTRIA • Tyrol: Innsbruck, Unikreuzung, under *Betula* and *Corylus*, 31.07.1997, leg. H. Ladurner, det. A. Biketova, IB 1997886 (GP); FINLAND • Nylandia: Helsinki, Elaeintarha, 60°11'17"N, 24°56'13"E, on lawn near *P.tremula*, 26.08.2013, leg. P. Hoeijer, det. A. Biketova & P. Hoeijer, H6045679 (GP); • Loviisa, Pernaja churchyard, 60°26'20"N, 26°02'46"E, under *Q.robur*, 09.09.2011, leg. P. Hoeijer, det. A. Biketova & P. Hoeijer, H6032689 (GP); • Porvoo, Kirkkotori, near the church, 60°23'49"N, 25°39'36"E, in lawn under T.×europaea, 16.08.2009, leg. P. Hoeijer, det. A. Biketova & T. Niskanen, H6036888 (GP); FRANCE • Île-de-France: Essonne, Massy, 48°39'22"N, 2°29'34"E, 80 m, fourteen young to mature basidiomes in the grass close to *Quercus* sp. and other broadleaved tress, 03.06.1994, leg. G. Redeuilh, det. G. Simonini, GS1332; • Grand Est: Haut Rhin, Rixheim, 47°44'40"N, 7°23'49"E, 253 m, twelve young to mature basidiomes in a grassy ground close to *Betula* tree, 07.10.2005, leg. E. Musumeci, det. G. Simonini, GS10414 (GP); GERMANY • Hesse: Giessen, Giessen Botanical Garden, next to the pond, mixed woodland, 10.06.1998, leg. & det. A. Taylor, K-M000168095 (AT1998006; GP) and AT1998007; GREECE • Central Macedonia: Kilkis, Mt. Belles, 41°18'36"N, 23°3'1"E, under *F.sylvatica*, 10.09.2009, leg. G. Konstantinidis, det. G. Konstantinidis, E. Polemis, B. Dima & A. Biketova, GK3993 (GP); • ibid. (5 km away from GK3993 location), under *F.sylvatica*, 10.09.2009, leg. G. Konstantinidis, det. G. Simonini, GK3982 (GS10502); HUNGARY • Bács-Kiskun: Lakitelek-Tőserdő, in park under *T.cordata*, 02.10.2022, leg. & det. L. Albert, AL 22-82 (GP); • Tompa, in the garden next to St. Anne’s Church, 46°10'58"N, 19°32'05"E, under *Quercus* sp. and *Acer* sp., two young basidiomes, 22.06.2018, leg. & det. A. Biketova K-M001435679 (AB B18-287); • Budapest: Soroksár, Botancial Garden, under *P.alba*, 18.10.2022, leg. L. Albert & B. Nagy, det. L. Albert, B. Nagy L. & B. Dima, AL 22-91 (GP); • Csongrád-Csanád: Szeged, in front of the Ferenc Móra College, in road border, under *Tilia* sp., 08.06.2013, DB5078; • Szeged, near Fesu str. 11, 46°14'35.3"N, 20°09'34.2"E, three mature basidiomes, under *Q.robur* and *T.cordata*, 06.06.2018, leg. & det. A. Biketova, K-M001435684 (AB B18-286); • Szeged, next to the Institute of Biology of the University of Szeged, 46°14'45.7"N, 20°10'03.6"E, under *T.cordata*, a single mature basidiome, 06.06.2018, leg. & det. B. Dima, K-M001435688 (AB B18-285); • Nógrád: Börzsöny Mts, near Diósjenő, 47°57'05.5"N, 18°59'21.0"E, under *C.betulus*, one young and one mature basidiome, leg. & det. L. Albert & A. Biketova, K-M001435711 (AB B18-399, AL 18-73); • ibid., 47°57'37.7"N, 19°00'31.6"E, in *Q.petrea*, *Q.cerris*, and *C.betulus* forest, two mature basidiomes, 24.06.2018, leg. & det. A. Biketova & B. Dima, K-M001435709 (AB B18-398); • ibid., under *C.betulus* and *S.alba*, 11.09.2022, leg. L. Albert, det. L. Albert & B. Dima, AL 22-75 (GP); • Pest: Üllő, under *Populus* hybrids, 03.08.2012, leg. L. Albert, det. L. Albert & B. Dima, AL 12-28 (GP); • ibid., under *Populus* hybrids, 25.07.2012, leg. L. Albert, det. L. Albert & B. Dima, AL 12-288 (GP); • ibid., 29.05.2014, leg. L. Albert, det. L. Albert & B. Dima, AL 14-19; • ibid., 04.09.2015, leg. L. Albert, det. L. Albert & B. Dima, AL 15-54 (GP); ITALY • Emilia-Romagna: Reggio nell’Emilia, parco del Popolo, 44°42'14"N, 10°37'49"E, 53 m, two mature basidiomes in the grass, close to *T.platyphyllos* and *P.alba* trees, 17.05.1999, leg. & det. G. Simonini, MCVE18373 (GS2077); • ibid., 44°42'12"N, 10°37'49"E, 53 m, a single young basidiome in the grass, close to *P.alba* trees, 05.06.2016, leg. & det. G. Simonini, GS10694; • Friuli-Venezia Giulia: Agordo, Belluno, Col di Foglia, 46°16'28"N, 12°02'39"E, 739 m, edge of a mixed forest, 30.07.2016, leg. E. Bizio, det. G. Simonini, GS10842 (GP); • Lazio: Rome, Via Appia Antica, 41°50'14"N, 12°32'20"E, 56 m, four very young to young basidiomes collected on loamy, very moist grassy soil under *T.platyphyllos*, Populusnigravar.italica, and *Q.ilex*, with the presence of *C.deodara*, *Eriobotryajaponica*, *Cupressussempervirens*, and *Fraxinusornus* in the garden of a private property, 23.10.2008, leg. & det. M. Gelardi, MG147; • ibid., a single mature basidiome (this basidiome belonged to the previous collection, but was left on the ground and collected five days after), 28.10.2008, leg. & det. M. Gelardi, MG147-bis; • ibid., three mature and one aged basidiomes, 03.11.2008, leg. M. Gelardi, det. M. Gelardi & A. Biketova, MCVE25582 (MG163, GP); • ibid., five basidiomes in various developmental stages, 08.10.2009, leg. and det. M. Gelardi, MG257; • ibid., five middle-age basidiomes (three out of which growing connate at the very stipe base), 10.11.2009, leg. & det. M. Gelardi, MG284; • ibid., a single mature basidiome, 29.04.2010, leg. & det. M. Gelardi, MG300; • ibid., several very young to mature basidiomes, 21.09.2010, leg. & det. M. Gelardi, MG341; • ibid., several very young to mature basidiomes, 17.11.2011, leg. & det. M. Gelardi, MG451; • ibid., several very young to mature basidiomes, 15.10.2013, leg. & det. M. Gelardi, MG567; • Rome, Consorzio Olgiata, Isola 32 B3, 42°01'29"N, 12°21'40"E, 133 m, several basidiomes in all developmental stages collected in loamy soil, on needle litter and among the grass under *C.atlantica* with the presence of *T.platyphyllos* and *P.pinea* in the garden of a private property, 11.10.2022, leg. & det. M. Gelardi, MG950; • Liguria: La Spezia, Deiva Marina, 44°13'16"N, 09°31'22"E, 12 m, artificially irrigated urban flowerbed, five young to mature basidiomes in the grass, soil pH = 7.5, close to *T.cordata*, 17.06.1994, leg. & det. G. Simonini, GS1138; • ibid., artificially irrigated urban flowerbed, 10 young to mature basidiomes in the grass, soil pH = 6.9. close to *T.cordata*, 18.06.1994, leg. & det. G. Simonini, MCVE17711 (GS1142); • ibid., artificially irrigated urban flowerbed, 12 young to mature basidiomes in the grass, soil pH = 6.9, close to *T.cordata*, leg. & det. G. Simonini, 20.06.1994, MCVE17712 (GS1143); • Lombardia: Brescia, Valsaviore, Cevo, 46°04'49"N, 10°22'47"E, 1190 m, on very dry, acid soil on a slight slope facing south, eleven very young to mature basidiomes collected in the grass and on decomposed litter of needles and dry leaves, in a sub-alpine mixed broadleaved/conifer thicket dominated by *C.avellana* and *P.abies* with the presence of *Juniperus* sp., ferns and nettles near a grassy pasture, with trees of *S.alba*, *Fraxinus* sp., and *Populusnigra* standing in the middle of the pasture, all within 30 m from the place of collection, 16.08.2009, leg. M. Gelardi & S. Gelardi, det. M. Gelardi, MCVE25592 (MG226); • ibid., a single mature basidiome, 20.08.2009, leg. & det. M. Gelardi, MG330; • ibid., 1230 m, a few hundred metres away from the previous collections, five middle-aged to mature basidiomes growing under a single young *P.abies* tree in the centre of a grassy clearing surrounded by a *P.abies* forest with the presence of *L.decidua* (without presence of broadleaved trees), 18.08.2009, leg. & det. M. Gelardi, MG231; • Sicily: Messina, Monti Peloritani, Mt. Dinnammare, Sentiero Ziriò, 38°10'36.7"N, 15°28'12.1"E, 800 m, five young to mature basidiomes growing with *Cedrus* sp., *Q.ilex*, *Pinus* spp., *Pseudotsugamenziesii*, and *Juglansregia*, 25.09.2019, leg. & det. S. Saitta, TUR-A 209256 (GP); • ibid., several basidiomes, 24.09.2020, leg. & det. S. Saitta, TUR-A 209574 (GP) and TUR-A 209575 (GP); • Trentino-South Tyrol: Trentino, Lake Pudro, Pergine Valsugana, 46°04'42"N, 11°13'16"E, 510 m, several young to mature basidiomes growing along a trackside in a riparian environment under *A.glutinosa*, *Populusnigra* and *P.tremula*, 26.08.2010, leg. M. Floriani & M. Gelardi, det. M. Gelardi, MG338; • Veneto: Sacca Sessola lagoon island, Venezia, 45°24'17"N, 12°19'23"E, 1 m, 14 basidiomes in the grass, close to *Cedrus*, other conifers and *Populus*, 13.10.1990, leg. E. Bizio, det. G. Simonini, GS1125; NETHERLANDS • South Holland: Leiden, Johan Wagenaarlaan, several partial fairy rings on lawn on sandy clay soil under *Tilia*, no other trees nearby, 05.09.1988, leg. & det. C. Bas, L0069896 (Bas C. 8633; GP); NORWAY • Oslo Co.: Oslo, Bygdoy, Ingstadasen, 59°54'29.8"N, 10°41'15.1"E, under *Tilia* and *Corylus*, 31.08.2017, leg. E. Bendiksen, det. B. Dima, O-F-254512 (GP); • Oslo, Tøyen, Botanical Garden, 59°54'59.8"N, 10°46'17.4"E, under *Tilia*, 06.07.2004, leg. A. Molia & E.W. Hanssen, det. A. Taylor, O-F-270491 (GP); • ibid., 59°55'16"N, 10°46'55"E, 05.09.2001, leg. G. Gulden, det. B. Dima, O-F-73245 (GP); • ibid., under *Tilia*, 07.09.2004, leg. G. Gulden, det. A. Taylor, O-F-67109 (GP); • ibid., 59°54'58"N, 10°46'08"E, *Piceaabies* dominated forest, 01.08.2014, leg. A.K. Wollan, det. A.K. Wollan, A. Taylor & B. Dima O-F-248341 (GP); • Østfold Co.: Moss, Melløsparken, 59°25'26"N, 10°40'05"E, under *Fagus* and *Betula* in park, 01.09.2019, leg. E.W. Hanssen & R. Braathen, det. B. Dima, O-F-307797 (GP); • Telemark Co.: Kragerø, Jomfruland, 58°52'52"N, 9°36'40"E, under *Corylus*, *Quercus*, *Pinus*, and *Salix*, 01.10.2022, leg. B. Rian & M.S. Olsen, det. B. Dima, O-F-204266 (GP); RUSSIA • Pskov Oblast: Pushkinogorsky District, A.S. Pushkin Museum-Reserve “Mikhaylovskoe”, 57°03'08"N, 28°55'04"E, on a grassy edge of mixed coniferous-deciduous forest, 11.09.2018, leg. O. Morozova, det. O. Morozova & T. Svetasheva, LE F-315962 (GP); • Tsevlo Village, remains of an abandoned park, on soil under old-growth *T.cordata*, 07.09.2019, leg. & det. L.B. Kalinina, LE F-330285 (GP); • Saint-Petersburg: Summer Garden, central part, 59°56'42"N, 30°20'06"E, on a lawn under *T.cordata*, 21.07.2017, leg. T. Svetasheva, det. A. Biketova & T. Svetasheva, LE F-332077 (GP); • ibid., in an open lawn with a single *T.cordata*, 21.07.2017, leg. T. Svetasheva, det. A. Biketova & T. Svetasheva, LE F-332078 (GP); • ibid., on a lawn under *T.cordata* and *Acerplatanoides*, 14.07.2020, leg. O. Morozova, det. O. Morozova & T. Svetasheva, LE F-315839 (GP); • ibid., in a grassy site under *Q.robur*, *T.cordata* and *Sorbusaucuparia*, 20.07.2017, leg. and det. T. Svetasheva, LE F-332081; • Summer Garden, northern part “Cross Arcade” bosquet, 59°56'47.6"N, 30°20'01.2"E, on a lawn under *P.abies* (the nearest *T.cordata* tree being 10 m away), 21.07.2017, leg. T. Svetasheva & O. Morozova, det. A. Biketova & T. Svetasheva, LE F-332079 (GP); • Summer Garden, between the central part and the “Cross Arcade” bosquet, 59°56'43"N, 30°20'07"E, under *T.cordata* and *P.abies* planting, among mosses on soil, 14.07.2020, leg. and det. T. Svetasheva & O. Morozova, LE F-315852 (GP); • ibid., on a lawn ca. 10–15 m from *P.abies*, 21.07.2017, leg. T. Svetasheva & O. Morozova, det. A. Biketova & T. Svetasheva, LE F-332080 (GP); • ibid., under *P.abies* and *T.cordata*, 06.08.2020, leg. O. Morozova, det. O. Morozova & T. Svetasheva, LE F-315838 (GP); • Summer Garden, site 5, 59°56'48"N, 30°20'10"E, under *T.cordata*, 10.09.2015, leg. E. Shalakitskaya, det. T. Svetasheva, LE F-315840; • Peter the Great Botanical Garden, near the building “Kizhi”, 59°58'9.4"N, 30°19'20.7"E, along a path, under *T.cordata* and *C.avellana*, 22.07.2011, leg. & det. O. Morozova & T. Svetasheva, LE F-254151 (GP); • Peter the Great Botanical Garden, near the Main Herbarium building, 59°58'9.7"N, 30°19'14.5"E, on a lawn under *T.cordata*, 21.07.2009, leg. & det. O. Morozova & T. Svetasheva, LE F-254144 (GP); • ibid., under *T.cordata*, 21.09.2010, leg. O. Morozova, det. T. Svetasheva, LE F-332075 (GP); • Tatarstan Republic: Elabuzhsky District, “Bolshoy Bor”, site 26, 55°45'34"N, 52°16'57"E, mixed coniferous-deciduos forest, 29.08.2013, leg K. Potapov, det. T. Svetasheva, LE F-315842; • Kazan, Gorky Central Park, under *Tilia* and *Betula* planting, 11.08.2015, leg. K. Potapov, det. T. Svetasheva, LE F-315844; • Zelenodolsky District, Volzhsko-Kamsky Nature Reserve, near “Dolgoye” bog, 55°54'54.0"N, 48°46'37.2"E, a single basidiome on the soil in deciduous forest (*Quercus*, *Tilia*, *Betula*), 24.08.2016, leg. M. Djakov, det. T. Svetasheva & G. Simonini, LE F-311914 (GS10807; GP); • Tula Oblast, Tula, Platonovsky Park, 54°9'6"N, 37°34'36"E, broadleaved forest, under *Q.robur* and *T.cordata*, 27.07.2016, leg. & det. T. Svetasheva, LE F-343931; • Udmurt Republic: Votkinskji District, 2 km SW of Novyi, 56°48'13"N, 54°00'45"E, 95 m, on sandy ground on the roadside, in *P.tremula* forest with *T.cordata* and *U.glabra*, 13.07.2014, leg. V.I. Kapitonov, det. G. Simonini, GS10506 (GP); • Izhevsk City, 56°52'32"N, 53°13'26"E, 159 m, two mature basidiomes on needle litter, in a city park close to P.×fennica forest with *T.cordata*, *Acernegundo* and *Populus* sp., 21.09.2013, leg. V.I. Kapitonov, det. G. Simonini, GS10514 (GP); SERBIA • Central Serbia: Mačva District, Banja Koviljača, 44°30'40"N, 19°9'33"E, 167 m, two young to middle-aged basidiomes growing on the ground among grass and moss along a pathway, under *T.cordata*, in a mixed broadleaf forest dominated by *C.betulus*, *F.sylvatica*, and *Q.cerris*, 08.08.2020, leg. A. Biketova & B. Dobrić, det. A. Biketova & B. Dima, K-M001435682 (AB B20-368; GP); SPAIN • Cantabria: Valle de Cabuérniga, Los Tojos, 43°09'34"N, 4°14'56"W, 399 m, three specimens growing on acidic soil, in a short slope close to *C.avellana* and *Populusnigra*, 06.09.2005, leg. J. Muñoz, det. J. Muñoz & A. Taylor, JAM0593 (GP); SWEDEN • Södermanland Co.: Stjärnhov, Solbacka Golf Course, under a single *Betula* sp., 14.07.2002, leg. & det. A. Taylor, AT2002036 (GP); SWITZERLAND • Zurich: Schwamendingen, 47°23'48"N, 8°34'14"E, 550 m, in the grass in an urban wood, close to *P.abies* and *F.sylvatica*, 20.08.1985, leg. C. Lavorato, det. G. Simonini, GS1541; UNITED KINGDOM • England: Berkshire, Silwood Park near Ascot, SU 946686, under *Carpinus*, *Tilia*, and *Cornus*, 27.09.2006, leg. A. Hills, det. A. Taylor, K-M000172000 (AH2006071; GP); • Greater London, Richmond, RBG Kew, along the way from Elizabeth Gate to Jodrell Lab, 51°29'01.3"N, 0°17'28.5"W, two mature basidiomes, among grass on the open lawn, in vicinity of *Magnolia* spp., *Q.ilex*, and Pinusnigravar.maritima, 19.10.2021, leg. A. Biketova, det. A. Biketova & G. Kibby, K-M001435675 (AB B21-381; GP).

#### Notes.

This bolete does not pose any problem from both the nomenclatural and taxonomic viewpoint. Originally described as a member of *Boletus*, it was then recombined in *Xerocomus* (Redeuilh 1993), in *Xerocomellus* (Mikšík 2014), and, finally, in *Hortiboletus* (Dima 2015).

This species was introduced by G. T. Oolbekkink and W. W. van Duin in a publication by Oolbekkink (1991) dealing with the ornamentation of *Xerocomus* basidiospores observed at SEM. The colour picture of *X.bubalinus* in Ladurner and Simonini (2003) refers to a topotypical collection; however, the picture was published with overexposed red tones, whereas the original image is much more brownish. A collection with features consistent with the original concept by Oolbekkink and Duin was described in detail from France (Alsace), in association with *Betula* on sandy soil (Musumeci 2008).

Based on the extended original description (Oolbekkink 1991) and our data, the basidiospore size of *H.bubalinus* is unusual for *Hortiboletus*, with a very high Q value = 2.46 ± 0.10 in average (up to 2.7) when compared with those of *H.rubellus* – 2.31 ± 0.12. Robinson et al. (2011) indicated frequent bifurcating or even trifurcating terminal elements of pileipellis. This feature was also highlighted in the line drawings of the pileipellis in Gelardi (2010). In addition, pileipellis subterminal elements are often wider than terminal ones, with typically inflated, branched and sometimes broadly rounded cells up to 30 μm broad (Gelardi 2009, 2010).

Based on the original diagnosis of *X.erubescens*Cadiñanos and Muñoz (1992), it shares with *H.bubalinus* some morphological features: the pinkish pileal context, the pileipellis structure, lack of reddish tints on both pileus (which is sometimes present in *H.bubalinus*) and stipe surfaces, and lack of orange-red dots in the stipe base. However, spores of *X.erubescens* are significantly larger [15.4–19.0(–20.3) × 5.7–7.2(–7.7) μm] according to the original diagnosis (Cadiñanos and Muñoz 1992), but they were re-analysed (isotype collection IB19920820 (BAR 1656/92) and IB19980600) by Ladurner and Simonini (2003), who obtained significantly smaller sizes [(11.0–)13.4 ± 1.04(–15.5) × (4.4)5.0 ± 0.31(–5.9) μm], when compared with spore sizes of some *H.bubalinus* collections (e.g. holotype L 0053449). Moreover, abnormally large spores (up to 19.0 × 7.5 μm) occur in some *H.bubalinus* collections, including its holotype (L 0053449).

In our multilocus analysis (Fig. 1), *H.bubalinus* forms a highly-supported terminal clade (BS = 94, PP = 1.00). In both ML and BI trees, it clusters in a highly-supported subgeneric clade (BS = 99, P P = 1.00), together with *H.arduinus* and three to four undescribed species: one from North America (USA) and two to three from Asia (China, Japan, and South Korea). However, there are minor differences in topology of this subgeneric clade in multilocus ML and BI trees. In multilocus ML phylogeny, *H.bubalinus* clusters as a sister clade to *Hortiboletus* sp. 7 (represented by LSU, *tef1-α*, and *rpb2* sequences of a single collection HKAS 51292) with weak statistical support (BS = 61). In multilocus BI phylogeny, *H.bubalinus* clusters together with a common branch of *Hortiboletus* sp. 4, *Hortiboletus* sp. 5, and *Hortiboletus* sp. 7 (all represented by only ITS sequences) with weak support (PP = 0.89).

Based on multilocus and four single-locus sequence data, two other European *Hortiboletus* species are significantly distant from *H.bubalinus* (Figs 1, 2). The closest species are *H.engelii* and *H.hershenzoniae* with a consistent, ca. 13% dissimilarity (> 100 nucleotide and indel differences). The ITS of *H.bubalinus* has been difficult to sequence with the classical Sanger method due to frequent polymorphisms, particularly in short insertion of AC repeats in ITS1 region, which vary in length within the species from 14 to 26 bp and even within the same genome with maximum observed difference of 12 bp (in JAM0593). Therefore, in some cases, cloning has been performed to obtain full ITS sequences (e.g. the holotype). Furthermore, *H.bubalinus* has one of the longest tandem repeats in the ITS2 region among other *Hortiboletus* species (see Table 1). Based on different alignment parameters of TRF analysis, the length of this long tandem repeat is estimated to be from 225–228 bp (2-7-7) to 231–253 bp (2-3-5). All species in *H.arduinus*/*H.bubalinus* subgeneric clade are characterised by such long minisatellite-like insertion in the ITS 2 region.

### ﻿ Hortiboletus engelii

Taxon classificationFungiBoletalesBoletaceae

(Hlaváček) Biketova & Wasser, Index Fungorum 257: 1 (2015)

8F08E27E-D1E9-53CD-9F87-36F20C56DD9E

MB551542

**Figure 11.**
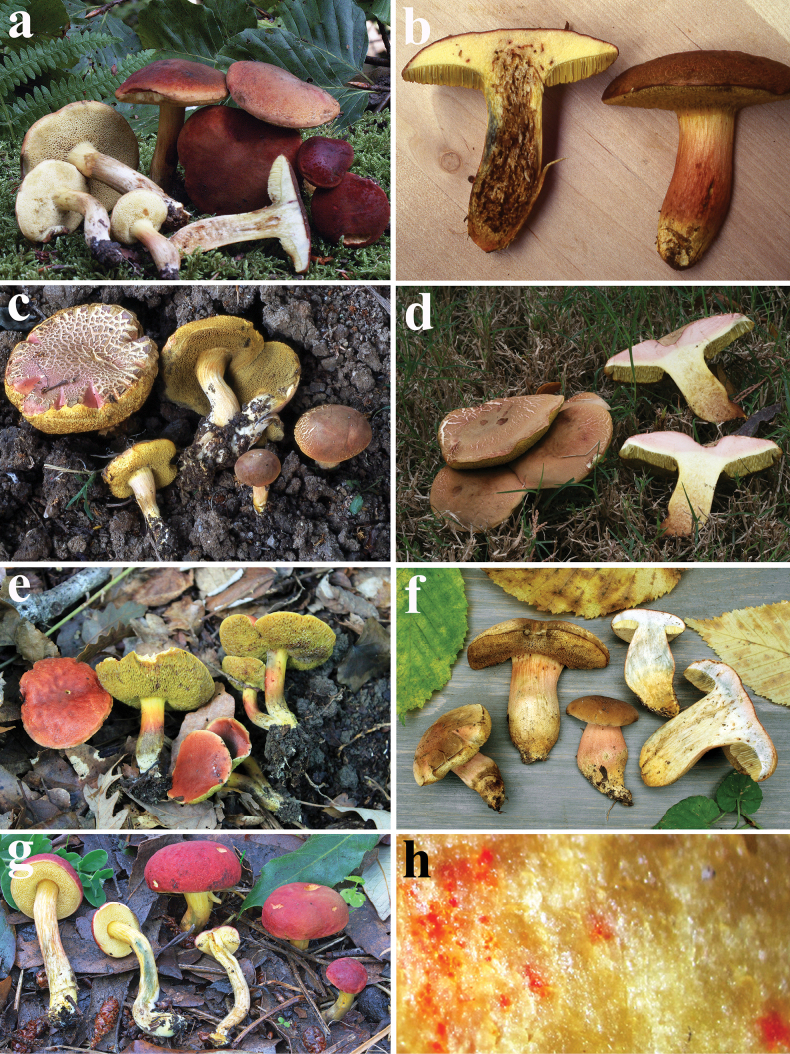
*Hortiboletusengelii* basidiomes: **a** JAM0547; **b** epitype collection MCVE18563; **c** MCVE18267; **d** GS10273; **e** GS10848; **f** AL21-73; **g** TUR-A209577; **h** orange-red dots in the stipe base context of GK6124. Photos: (**a**) J. A. Muñoz; (**b**) A. F. S. Taylor; (**c**) G. Simonini; (**d**) A. Mua; (**e**) M. Raumi; (**f**) L. Albert; (**g**) S. Saitta; (**h**) G. Konstantinidis.

**Figure 12.**
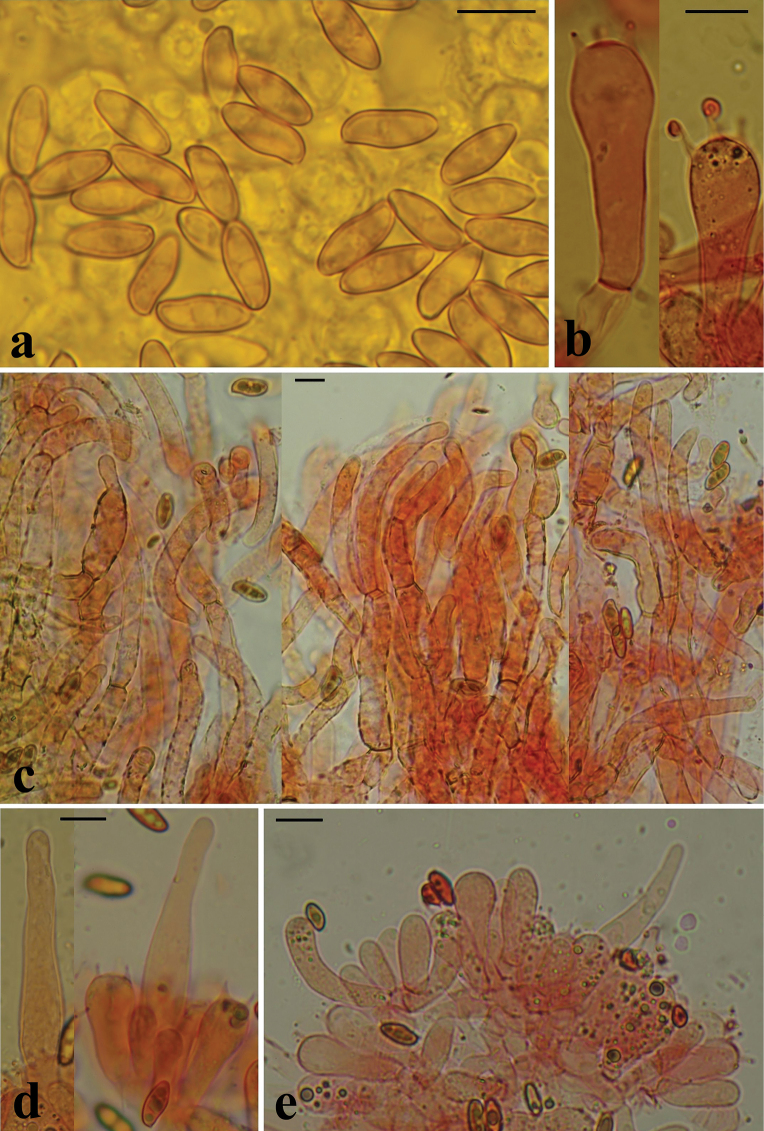
Microscopical features of *H.engelii*: **a** basidiospores in L4; **b** basidia in Congo red; **c** pileipellis in Congo red; **d** pleurocystidia in Congo red; **e** tuft of cheilocystidia in Congo red. All photos from collection GS10409. Scale bar: 10 μm. Photos: G. Simonini.

Figs 3b , 8 , 11 , 12

≡ Boletusengelii Hlaváček, Mykologický Sborník **78**: 67 (2001)
≡ Xerocomusengelii (Hlaváček) Gelardi, Boll. Assoc. Micol. Ecol. Romana **24–25**(75–76): 18 (2009) [2008]
≡ Xerocomellusengelii (Hlaváček) Šutara, Czech Mycol. **60**(1): 49 (2008)
= Xerocomusquercinus H. Engel & T. Brückn., in Engel, Dermek, Klofac, Ludwig & Brückner, Schmier- und Filzröhrlinge s.l. in Europa: 205 (1996), *nom. inval.*, *nom. prov.*, Arts 36.1(a), 39.1, 40.1 (Shenzhen)
? = Boletuscommunis Bull., Herb. Fr.**9**: tab. 393 (1789), s. auct.
= Xerocomuscommunis (Bull.) Bon, Docums Mycol. **14**(56): 16 (1985) [1984], s. auct.
= Ceriomycescommunis (Bull.) Murrill, Mycologia **1**(4): 155 (1909), s. auct.
= Boletuscommunis Sowerby, Coloured Figures of English Fungi**2**: 94, pl. 225 (1799), *nom. illeg.*, s. auct.
? = Boletussubtomentosussubsp.declivitatum C. Martín, Matériaux pour la Flore Cryptogamique de la Suisse **2**(1): 18 (1904), s. auct.
= Boletusdeclivitatum (C. Martín) Watling, Edinb. J. Bot. **61**(1): 43 (2004), s. auct.
= Xerocomusdeclivitatum (C. Martín) Klofac, Öst. Z. Pilzk. **16**: 258 (2007), s. auct.

#### Holotype.

GERMANY • Thuringia: Gera, MTB 5138/1, Weinberg (vineyard), on the ground, 250 m, 1989, leg. T. Brückner.

#### Description.

***Basidiomes*** pileate-stipitate, xerocomoid, epigeal, small to medium-small sized. ***Ontogenetic development*** gymnocarpic.

***Pileus*** (4–)5–13(–15) cm broad, nearly hemispherical at first, then convex, pulvinate to applanate, also slightly depressed at centre, sometimes irregularly shaped; surface matt, dry, ± finely tomentose to sometimes rugulose, finely areolate towards the peripheral zone or with minute to coarse cracks over the whole surface especially with dry weather and showing a yellowish or somewhat pinkish subpellis layer resembling some areolate *Xerocomellus* species (Simonini 1998b); margin initially involute then extended or even uplifted, regular to wavy-lobed, acute and slightly exceeding the pileal context, whitish at the very beginning for a thin strip, but soon concolourous; of extremely variable colours, but generally dark brown up to black-brown at the beginning, then fading to ochraceous, ochraceous yellow, buff, yellowish-brown, olivaceous brown, greyish-brown, olive grey, grey or also pinkish-red, orange-red, ochraceous red, ochraceous pink, reddish-brown, typically fading in age, although some basidiomes maintain a carmine red tint over the entire life-cycle; unchanging or becoming slightly darker on handling or when injured.

***Tubes*** up to 15 mm long, at first adnate, later emarginate or slightly decurrent with a tooth, initially lemon or chrome yellow, later greenish-yellow to olive brown, unchangeable to weakly bluing when injured.

***Pores*** large, wider than 1 mm (up to 1.5–3 mm) in old basidiomes, roundish to angular, concolourous with the tubes, in mature basidiomes with orange-rusty spots, unchangeable to weakly bluing when bruised and later becoming dirty brownish.

***Stipe*** (2–)2.5–7(12) × 0.6–3.5(–4.5) cm, usually as long as the pileus diameter or slightly longer, central or slightly off-centre, solid, firstly versiform, clavate or somewhat bulbose, later more or less cylindrical or sometime inflated in the lower part, not infrequently stout, bulbose to ventricose-subfusiform, always tapering at the very base and slightly rooting; surface with very fine fibrils or scales or, occasionally, very finely ribbed, which may be reddish on an ochraceous-yellow background or concolourous with the background, later often with rather coarse longitudinal striations; yellow at apex; sometimes the stipe can be entirely yellow, entirely red, or a mix of both colours; some basidiomes exhibit a thin, lemon-yellow cortex shaded of carmine red at base; unchangeable or very slowly bruising pale brown on handling; basal mycelium whitish-yellow, rarely bright yellow.

***Context*** rather firm when young, later soft in the pileus (up to 2.4 cm thick in the central zone, up to 1.0 cm thick halfway to margin and gradually becoming thinner towards the edge), becoming fibrous in the stipe; generally pale yellow, but dirty ochraceous yellow in the lower half of the stipe and eventually (especially in collections recorded late in the season and with low temperatures) reddish for a few mm underneath the cuticle, in the stipe base with a typical carrot-orange or orange-red pigment in the form of small dots (“*ponctuation de Redeuilh*”), which are sometimes scarce and hardly noticeable or conversely abundant (especially in dry conditions) and then visible as a large flame-orange area at the stipe base; light blue on exposure, especially in the pileus-stipe connection zone, above the tubes and in the peripheral layers of the stipe, later fading to dirty yellow; discoloration less pronounced in dry and old basidiomes.

***Spore print*** olive brown.

***Odour*** pleasantly fungoid to fruity or inconspicuous. ***Taste*** mild.

***Macrochemical spot-test reactions***: Melzer’s reagent: a weak fleeting-amyloid reaction in the stipe context and exceptionally also in the hymenophoral trama of dried basidiomes sometimes occurs, but most collections show a strong dextrinoid reaction in all tissues; 20% KOH: reddish-brown on pileus and hymenophore, pinkish on pileus context, bright orange on stipe surface and context and progressively darker downwards; 25% NH_4_OH: none to pale yellowish everywhere; 10% FeSO_4_: sordid green everywhere, particularly on the hymenophore.

***Basidiospores*** [1691/62/61] (10.0–)12.1 ± 0.8(–14.2) × (4.7–)5.1 ± 0.3(–5.9) μm, Q = (2.04–) 2.35 ± 0.13(–2.57), Vm = 171 ± 30 μm^3^, inequilateral, broadly ellipsoid or fusiform to ellipsoid-fusiform in side view, narrowly ellipsoid to amygdaliform or subfusiform in face view, smooth under light microscope, apex rounded, with a short apiculus, usually with a weak to pronounced suprahilar depression and with a slightly pronounced adaxial swelling, moderately thick-walled (up to 1 μm), straw yellow to honey yellow in water and 5% KOH, having one or two large oil droplets when mature, rarely pluri-guttulate, inamyloid and orthochromatic.

***Basidia*** [153/7/7] (22.9–)36.6 ± 5.4(–54.8) × (8.6–)11.3 ± 1.3(–15.2) μm, subclavate to clavate, predominantly 4-spored, sterigmata (1.5–)3.2 ± 1.1(–5.0) μm long, hyaline to yellowish in 5% KOH and containing concolourous oil guttules, without basal clamps; basidioles subcylindrical to faintly clavate, smaller than basidia.

***Cheilocystidia*** [18/2/2] (49.0–)69.8 ± 9.7(–85.8) × (7.7–)10.0 ± 2.2(–16.0) μm, scattered, slender, ventricose-fusiform, often with elongated straight or flexuous neck, with rounded tip, smooth, mostly hyaline to pale yellowish in water and 5% KOH. ***Pleurocystidia*** [87/8/8] (39.1–)65.5 ± 13.6(–99.3) × (7.5–)10.3 ± 1.9(–15.9) μm, infrequent, slender, ventricose-fusiform, often with an elongated straight neck, with rounded tip, smooth, mostly hyaline to pale yellowish in water and 5% KOH. ***Pseudocystidia*** not observed.

***Hymenophoral trama*** bilaterally divergent, intermediate between the “*Phylloporus*-type” and the “*Boletus*-type”: lateral strata consisting of poorly divergent, tightly arranged, non-gelatinous hyphae almost touching each other, hyaline to very pale yellowish in water and 5% KOH; mediostratum consisting of tightly adpressed, non-gelatinous, parallel running hyphae; in Congo red, the mediostratum is slightly darker than the lateral strata.

***Pileipellis*** a physalo-palisadoderm consisting of moderately long, more or less cylindrical, septate hyphae; ***terminal elements*** [660/22/22] (24.7–)36.5 ± 11.0(–63.3) × (8.4–)11.3 ± 2.2(–17.3) μm, Q = (1.93–)3.47 ± 1.58(–7.46), versiform, mainly broadly cylindrical with rounded apex to cystidioid, sometimes also with elongated neck, but also slender, narrowly cylindrical, moderately clavate, slightly tapering towards the tip, utriform, acorn-shaped, apex mainly rounded-obtuse, but sometimes pointed, finely encrusted, more intensely and coarsely downwards (with fine granular or rarely zebra-like epiparietal encrustations), hyaline to yellowish in water and 5% KOH; ***subterminal elements*** subcylindrical, clearly restricted at septa, equal or broader than terminal ones, more frequently and coarsely encrusted, yellowish-brown in 5% KOH. ***Subpileipellis elements*** usually distinctly encrusted by a fine, yellowish, granular pigment (5% KOH).

***Stipitipellis*** consisting of slender, subparallel, smooth walled, hyaline to pale-yellow hyphae in 5% KOH, 4.5–6(–9) μm broad, ***caulohymenium*** hardly observable and not well developed; in the upper third of the stipe, infrequent bundles of clavate ***caulobasidia*** (25–35 × 8–13 μm), hyaline to yellow in 5% KOH and broadly ventricose to spherical ***caulobasidioles*** with a thin neck, (8–)9–12(–15) μm broad are usually present; furthermore, finely encrusted, hyaline, cylindrical, broad ***terminal cells*** with a round tip, (8–)10–12(–14) μm, similar to those of the pileipellis appearing here and there. ***Stipe texture*** apparently not differentiated from the stipe trama. ***Lateral stipe stratum*** not observed.

***Stipe trama*** consisting of parallel running hyphae, 5.0–8.5 μm broad, ± hyaline in water and 5% KOH, inamyloid; infrequent thromboplerous hyphae 6.0–8.5 μm broad are observed.

***Clamp connections*** absent in all tissues.

#### Ecology and phenology.

solitary, gregarious in small groups or even subcaespitose, growing on bare soil, on litter or in the grass, often in anthropogenic environments, shrublands, less frequently in undisturbed forests, under a large array of ectomycorrhizal broadleaved trees and shrubs, including preferably *Quercus* spp. (*Q.cerris*, *Q.coccifera*, *Q.frainetto*, *Q.ilex*, *Q.incana*, *Q.robur*, *Q.petraea*, *Q.pubescens*, *Q.suber*), *Fagussylvatica*, *Tilia* spp. (*T.cordata*, *T.platyphyllos*), *Carpinus* spp. (*C.betulus*, *C.orientalis*), sometimes also *Betula* spp., *Castaneasativa*, *Corylusavellana*, *Halimiumhalimifolium*, *Pinus* spp. (*P.halepensis*, *P.pinea*, *P.sylvestris*, *P.wallichiana*), and *Populus* spp. (*P.alba*, *P.tremula*) (Engel et al. (1996); Ladurner and Simonini (2003); Peintner et al. (2003), GP; Krpata et al. (2008), GP; Sarwar et al. (2016), GP; Leonardi et al. (2020), GP; pers. obs., GP). Some collections of *H.engelii* have been found in mixed forests with the presence of *Piceaabies* and *Ostryacarpinifolia*, but there are no findings in monodominant forest communities formed by these tree species alone. Moreover, there are some indications of a possible association with *Cedrusdeodara* (Kjøller in litt.; Szmidla et al. in litt.; pers. obs.), but these data have not yet been confirmed by molecular studies nor by our examined material. The main fruiting period is spring (late April) to autumn and early winter in the Mediterranean area (November to early December).

#### Known distribution (see Fig. 5a).

Asia: Pakistan (Sarwar et al. (2016), as *H.rubellus*, GP); Europe: Austria (Engel et al. (1996), as *X.quercinus*; Krpata et al. (2008), as *X.rubellus*, GP; Gelardi (2009)), Bulgaria (Assyov in litt.), Croatia (Peintner et al. (2003), as *X.rubellus*, GP; this study, GP), Czechia (Hagara et al. 1999; Šutara et al. 2009; Mikšík 2017; this study, GP), Denmark (Kjöller unpubl., as *X.communis*, GP), France (Engel et al. (1996); Lannoy and Estadès (2001), as *X.quercinus*; Suz et al. (2014), as *Boletus* sp., GP; Van Vooren et al. (2023), as *X.communis*; this study); Germany (Engel and Brückner (1989); Engel et al. (1996), both as *X.quercinus*; Engel (1999), as *X.communis*; this study, GP), Greece (Konstantinidis 2014; this study, GP), Hungary (Albert (2003); Dima et al. (2013), both as *X.communis*; this study, GP), Italy (Simonini (1998a); Consiglio and Papetti (2001); Taylor et al. (2001); Cazzoli (2002); Galli (2013); Vasquez (2014), all as *X.communis*; Ladurner and Simonini (2003), included in *X.rubellus*; Leonardi et al. (2020), as *H.rubellus*, GP; this study, GP), Netherlands (Noordeloos 2007), Malta (Mifsud and Mifsud unpubl., GP), Montenegro (Lazarevic et al. unpubl., as *X.rubellus*, GP), Norway (Knudsen and Taylor 2008), Poland (Szmidla et al. unpubl., as *B.rubellus*, GP), Romania (Suz et al. (2014), GP); Russia (this study, GP), Serbia (Uzelac (2009), as *X.communis*; this study, GP), Spain (Calzada Domínguez (2007); Muñoz et al. (2008), both as *X.communis*; this study, GP), Sweden (Taylor and Eberhardt (2006); Knudsen and Taylor (2008), all as *X.communis*; this study, GP), Ukraine (this study, GP), United Kingdom (Taylor et al. (2002); Hills (2008), both as *X.communis*; Watling and Hills (2005); Hills (2007), both as *Boletusdeclivitatum*; Ainsworth et al. (2013), as *B.declivitatum*; Kibby (2017); Ainsworth et al. (2024), as *Hortiboletus*, GP; this study, GP); North America: USA (Murrill 1909; D’Elia et al. unpubl., GP). Records from eastern Asia (Zang 2006), usually referred to as *Xerocomuscommunis* or *B.communis* should be carefully re-evaluated.

#### Examined material.

CROATIA • Lošinj: Veli Lošinj, under *Q.ilex* and *Oleaeuropaea*, 08.10.1999, leg. H. Ladurner, det. E. Polemis & A. Biketova, IB19990917 (GP); CZECHIA • South Moravian Reg.: Brno, hills above Dubnany, on soil under *Quercus* sp., 12.08.2008, leg. A. Hills, det. A. Biketova, K-M000170238 (AH2008092; GP); FRANCE • Auvergne-Rhône-Alpes: Allier, Montluçon, the city park, 46°20'11"N, 02°37'01"E, 240 m, three young to mature basidiomes close to *Quercus* sp. trees, 10.10.1986, leg. G. Redeuilh, det. G. Simonini, MCVE17457 (GS1121); • Bretagne: Côtes-d’Armor, Plévenon, Cap Fréhel, 48°39'30"N, 02°19'17"E, 58 m, five mature basidiomes among the grass under *Q.robur*, 02.08.1987, leg. G. Reduilh, det. G. Simonini, GS1117; • Île-de-France: Paris, Pré Catelan, 48°51'53"N, 02°15'09"E, 44 m, a group of young and mature basidiomes close to *Quercus* sp., 22.08.1984, leg. G. Redeuilh, det. G. Simonini, MCVE17456 (GS1118); • Yvelines, Achères, forest east of the city, 48°57'42"N, 02°04'33"E, 27 m, three mature basidiomes in the grass at the edge of the *Quercus* sp. forest, 25.07.88, leg. G. Redeuilh, det. G. Simonini, GS1122; • Yvelines, Versailles, Château de Versailles, 48°48'10"N, 02°07'57"E, 133 m, a couple of one young and one mature basidiome, in the grass, under *Quercus* sp., 18.07.1993, leg. G. Redeuilh, det. G. Simonini, MCVE17679 (GS1123); • Essonne, Massy, Parc de la Tuilerie, 48°43'36"N, 02°16'28"E, 70 m, one basidiome in the grass close to *Quercus* sp. trees, 03.06.1994, leg. G. Redeuilh, det. G. Simonini, MCVE 17836 (GS1338); • Nouvelle Aquitaine: Creuse, Guéret, the city park, 46°09'46"N, 01°51'50"E, 544 m, in the grass, close to *Quercus* sp. trees, 11.10.1986, leg. G. Reduilh, det. G. Simonini, MCVE17458 (GS1120); GERMANY • Hesse: Giessen, old graveyard, under *Quercus* sp., 30.05.1998, leg. & det. A. Taylor, AT1998122 (GP); • Wiesbaden, Park “Unter den Eichen”, 50°05'42.2"N, 08°13'05.9"E, 212 m, one mature basidiome under old *Quercus* sp. tree, 18.06.1998, leg. & det. A. Taylor, MCVE18563 (AT1998001, GS2230; GP); • Rhineland-Palatinate: Mainz, Ober Olmer Wald, mixed woodland, but mainly *Quercus*, 26.06.1998, leg. & det. A. Taylor, AT1998032 (GP); • Mainz, Kloster Ebersbach, under *Quercus*, 28.06.1998, leg. & det. A. Taylor, AT1998037 (GP); GREECE • Attica: Tatoi, 38°09'39"N, 23°47'47"E, 500 m, under *Q.ilex* and *Q.coccifera*, on calcareous soil, 10.11.2012, leg. E. Polemis, det. E. Polemis & A. Biketova, ACAM2012-105 (GP); • Ionian Islands: Corfu, Sinies, 39°46'16"N, 19°56'38"E, under *Quercus*, 17.11.2018, leg. G. Konstantinidis, det. G. Konstantinidis, E. Polemis, B. Dima & A. Biketova, GK11644 (GP); • South Aegean: Cyclades, Amorgos Island, 36°50'57"N, 25°53'30"E, 300 m, under *Q.coccifera*, on acidic soil with goat manure, 06.12.2005, leg. E. Polemis, det. E. Polemis & A. Biketova, EP05-M224 (GP); • West Macedonia: Grevena Town, 40°4'25"N, 21°25'42"E, under *C.orientalis* and *Robiniapseudoacacia*, 28.09.2009 and 06.10.2015, leg. G. Konstantinidis, det. G. Konstantinidis, E. Polemis, B. Dima & A. Biketova, GK4141 (GP) and GK8378 (GP); • Grevena, Gorgiani, 39°56'41"N, 21°18'17"E, under *Q.frainetto*, 02.07.2012 and 24.09.2015, leg. G. Konstantinidis, det. G. Konstantinidis, E. Polemis & A. Biketova, GK6124 (GP) and GK8287 (GP); • Grevena, 1 km from Grevena to Elato, 40°5'45"N, 21°26'50"E, under *C.orientalis*, *R.pseudoacacia* and *C.avellana*, 04.10.2016, leg. G. Konstantinidis, det. G. Konstantinidis, E. Polemis, B. Dima & A. Biketova, GK9736 (GP); HUNGARY • Bács-Kiskun: Tompa, 46°10'10"N, 19°32'28"E, under *Q.robur*, growing together with *Russula* sp., 10 basidiomes, 22.06.2018, leg. A. Biketova & Z. Merényi, det. A. Biketova, K-M001435691 (AB B18-288); • Csongrád-Csanád: Sándorfalva, under *P.alba*, 08.06.2013, leg. & det. B. Dima, DB5077 (GP); • ibid., 46°23'34"N 20°07'36"E, under *Q.robur* and *R.pseudoacacia*, 06.06.2018, leg. & det. A. Biketova & B. Dima, K-M001435680 (AB B18-284); • Fejér: Velence, in park under *C.avellana*, 06.06.2016, leg. P. Finy., det. B. Dima, FP-2016-06-06 (GP); • Heves: Tarnalelesz, under *C.betulus*, 06.09.2015, leg. & det. L. Albert, AL 15-56 (GP); • Komárom-Esztergom: Vértes Mts, Tatabánya, under *Q.cerris* and *Q.petraea*, 12.06.2022, leg. & det. A. Nagy, B. Dima & Gy. Vrba, DB-2022-06-12-3 (GP); • Pest: Visegrád Mts, Pilisszentlászló, under *C.betulus*, 15.08.2021, leg. & det. L. Albert, AL 21-73 (GP); ITALY • Emilia-Romagna: Reggio nell’Emilia, Parco San Lazzaro, 44°41'20"N, 10°39'36"E, 48 m, more than 20 basidiomes from young to mature, under the monumental *Q.robur*, 13.09.1993, leg. & det. G. Simonini, MCVE17470 (GS986); • Reggio nell’Emilia, city centre, Viale Umberto I, 44°41'25"N, 9°49'58"E, 80 m, seven young to mature specimens under *T.platyphyllos*, 05.10.1990, leg. unknown, det. G. Simonini, GS760; • Reggio nell’Emilia, Viale Isonzo, 44°42'13"N, 10°37'43"E, 51 m, two mature basidiomes in a flower bed close to *T.platyphyllos* trees, 27.08.1995, leg. A. Nuccio, det G. Simonini, MCVE17905 (GS1417); • ibid., 44°42'14"N, 10°37'52"E, 69 m, five young to mature basidiomes growing in an urban flower bed, under *T.platyphyllos*, 21.06.1998, leg. & det. G. Simonini, MCVE18267 (GS1896; GP); • Reggio nell’Emilia, Parco del Popolo, 44°42'09"N, 10°37'52"E, 52 m, one large mature basidiome under *Q.robur*, 11.09.1993, leg. R. Meschieri, det. G. Simonini, MCVE17468 (GS973); • ibid., 44°42'13"N, 10°37'49"E, 53 m, in the grass close to *T.platyphyllos* and *P.alba*, 22.08.2015, leg. & det. G. Simonini, GS10138 (GP); • Reggio nell’Emilia, via Emilia San Maurizio, close to pharmacy “Caravita”, 44°41'37"N, 10°38'42"E, 54 m, six large mature basidiomes in a flower bed, under *P.abies* and *T.platyphyllos*, 28.06.1992, leg. & det. G. Simonini, GS885; • ibid., one mature basidiome in a flower bed, under *P.abies* and *T.platyphyllos*, 17.05.1993, leg. G. Fontana, det. G. Simonini, GS938; • ibid., 11 young to mature basidiomes in a flower bed, under *P.abies* and *T.platyphyllos*, 16.06.1995, leg. G. Fontana, det. G. Simonini, MCVE17838 (GS1370); • Reggio nell’Emilia, residential district “Pappagnocca”, 44°40'54"N, 10°38'38"E, 64 m, a single huge basidiome among the grass, under *Q.robur* trees, 15.10.1991, leg. unknown, det. G. Simonini, MCVE21790 (GS857); • Reggio nell’Emilia, Viale Ramazzini, 44°42'09"N, 10°38'14"E, 51 m, one young and one mature large basidiomes, among the grass in a garden in urban area near a young *Q.cerris* tree, 13.06.1992, leg. M. Comuzzi, det. G. Simonini, GS869; • ibid., four young to mature basidiomes among the grass in a garden in urban area, near a young *Q.cerris* tree, 24.05.1993, leg. M. Comuzzi, det. G. Simonini, GS939; • ibid., four mature basidiomes with cracked cuticle, among the grass in a garden in an urban area near a young *Q.cerris* tree, 04.09.1993, leg. M. Comuzzi, det. G. Simonini, GS958; • ibid., three young to mature basidiomes, among the grass in a garden in an urban area near a young *Q.cerris* tree, 05.09.1993, leg. M. Comuzzi, det. G. Simonini, GS961 (GP); • ibid., three mature basidiomes, among the grass in a garden in an urban area near a young *Q.cerris* tree, 07.09.1993, leg. & det. G. Simonini, MCVE17463 (GS964); • Reggio nell’Emilia, Cadelbosco di Sopra, Cadelbosco di Sotto, 44°48'19"N, 10°37'06"E, 25 m, urban green areas, in the grass close to *Quercus* sp. trees, 10.11.1996, leg. G. Candiani, det. G. Simonini, MCVE18151 (GS1759); • Albinea, Ca’ de Caroli, via Mattaiano, 44°35'14"N, 10°38'03"E, 350 m, five mature basidiomes at the edge of the cart track, close to *Quercus* trees, 17.10.1993, leg. & det. G. Simonini, GS1030; • Bergogno, via Bergogno, 44°33'02"N, 10°29'14"E, 590 m, two mature basidiomes at the road edge, close to *C.sativa* wood, 19.09.03, leg. & det. G. Simonini, GS1006; • Case Bigi, Parco del Rio Coviola, 44°40'55"N, 10°32'59"E, 70 m, in the mixed broadleaved wood along a small river, eight basidiomes from mid-age to mature, close to *Q.cerris*, 18.10.1998, leg. & det. G. Simonini, MCVE18353 (GS2028); • Codemondo, Parco del Rio Coviola, 44°40'42"N, 10°32'56"E, 94 m, in the mixed broadleaved wood along a small river, two mature basidiomes, close to *Quercus* sp., 24.08.95, leg. L. Vescovi, det. G. Simonini, MCVE17904 (GS1410); • Gattatico, Praticello di Gattatico, 44°48'25"N, 10°28'21"E, 37 m, five mature basidiomes under *Q.robur* trees, 7.10.1983, leg. G. Bramini, det. G. Simonini, MCVE17231 (GS71); • ibid., 44°48'17"N, 10°28'18"E, 35 m, green urban area, three mature basidiomes in the grass, close to *Q.robur* trees, 12.10.1998, leg. G. Donelli, det. G. Simonini, GS2009; • ibid., 44°48'21"N, 10°27'58"E, 25 m, six mature big basidiomes in the grass, green urban area close to *Acercampestre* and *Q.robur*, 12.10.1998, leg. G. Bramini, det. G. Simonini, MCVE18346 (GS2022); • ibid., 44°48'21"N, 10°27'55"E, 36 m, three mature basidiomes grown in an orchard (*Rosaceae*), apparently far from *Tilia* or *Fagales*, 01.11.2001, leg. G. Donelli, det. G. Simonini, GS2148; • Quattro Castella, Parco di Roncolo, 44°37'16"N, 10°29'04"E, 360 m, eleven partially dehydrated mature basidiomes, in a *Q.pubescens* wood, on calcareous soil, 20.06.1992, leg. & det. G. Simonini, GS874; • ibid., 44°37'24"N, 10°22'23"E, 350 m, two mature basidiome, in a *Q.pubescens* wood on calcareous soil, 21.06.1992, leg. & det. G. Simonini, GS878; • ibid., 44°37'17"N, 10°29'11"E, 380 m, one mature basidiome, in a *Q.pubescens* wood, on calcareous soil, 21.06.1992, leg. & det. G. Simonini, GS879; • ibid., 44°37'27"N, 10°29'24"E, 320 m, five mature basidiomes in the *Q.pubescens* wood, calcareous soil, 19.09.1993, leg. & det. G. Simonini, GS1009; • ibid., 44°37'28"N, 10°29'24"E, 315 m, three young to mature basidiomes in mixed broadleaved wood, close to *Q.pubescens* trees, 12.10.1996, leg. & det. G. Simonini, MCVE18129 (GS1714); • ibid., 44°37'19"N, 10°29'15"E, 399 m, six basidiomes mid-age to mature, in two co-grown tufts, in a wood of *Q.pubescens* and *O.carpinifolia* on calcareous soil, 20.10.2010, leg. & det. G. Simonini, GS11010; • ibid., 44°37'22"N, 10°29'14"E, 359 m, a single mature basidiome grown in the grass at the edge of a wood of *Q.pubescens* and *O.carpinifolia* on calcareous soil, 09.10.2010, leg. & det. G. Simonini, GS11011; • Reggio nell’Emilia, Quattro Castella, Rocca di Monte Lucio, 44°37'36"N, 10°27'56"E, 250 m, six mature basidiomes in the grass, close to *Q.pubescens* trees, 26.09.1994, leg. L. Vescovi, det. G. Simonini, MCVE17775 (GS1278); • ibid., 44°37'35"N, 10°27'49"E, 280 m, seven mature basidiomes in the grass, close to *Q.pubescens* trees, 26.09.1994, leg. L. Vescovi, det. G. Simonini, MCVE17769 (GS1279); • Parma, Sala Baganza, Carrega Woods Regional Park, near Lago della Svizzera, 44°43'49"N, 10°12'16"E, 153 m, a single isolated basidiome on the shore of the lake, with *C.betulus* and *Q.petraea*, 09.10.2015, leg. T. Svetasheva, det. G. Simonini, GS10409 (GP); • ibid., 44°43'47"N, 10°12'16"E, 154 m, a single isolated decayed basidiome on the shore of the lake, with *C.betulus*, *Q.petraea*, and *Taxodiumdistichum*, 09.10.2015, leg. T. Svetasheva, det. G. Simonini, GS10410 (GP); • Lazio: Rome, Via Appia Antica, 41°50'14"N, 12°32'20"E, 56 m, a single mature basidiome and a primordium collected on loamy, very moist grassy soil under *Q.ilex* and *Ligustrumlucidum* with the presence of bamboo sticks in the garden of a private property, 14.07.2009, leg. & det. M. Gelardi, MG205; • Liguria: La Spezia, Deiva Marina, 44°13'16"N, 09°31'22"E, 12 m, two mature basidiomes in the grass in an urban flowerbed artificially irrigated, close to *T.cordata* trees, soil pH = 6.9, 20.06.1994, leg. & det. G. Simonini, MCVE17713 (GS1144); • La Spezia, Portovenere, Muzzerone, 44°03'25"N, 9°49'58"E, 250 m, three large basidiomes, from mid-age to mature, in a sparse forest of *Q.ilex*, *Q.cerris*, *P.halepensis*, and *Ericaarborea*, 18.10.1987, leg. C. Fantuzzi, det. G. Simonini, MCVE21796 (GS530); • Lombardia: Milan, Paderno Dugnano, the city park “Emilio de Marchi”, 45°34'28"N, 09°09'47"E, 165 m, three young to mature basidiomes close to *Q.ilex* and *Q.robur*, together with many other broadleaved and conifer trees, 19.09.1993, leg. G. Pina, det. G. Simonini, GS1166; • ibid., ten and seven young to mature basidiomes, 06.09.1987 and 15.07.1989, leg. G. Pina, det. G. Simonini, GS1154 and GS1159; • ibid., one mature basidiome, 17.10.1990 and 28.07.1993, leg. G. Pina, det. G. Simonini, GS1163 and GS1165; • three basidiomes, 04.07.1993. leg. G. Pina, det. G. Simonini, GS1164; • Sardinia: Sassari, Calangianus, “Le Grazie”, under *Q.suber*, 05.09.1997, leg. M. Contu, det. A. Biketova, IB1997735 (GP); • Cagliari, Sinnai, Casula Mua Sanna, city park, 39°18'08"N, 9°12'11"E, 141 m, five young to mature basidiomes growing among the grass close to *Q.ilex* and *Cupressushorizontalis*, 01.12.2014, leg. A. Mua, det. G. Simonini, GS10273 (GP); • Sicily: Messina, Monti Peloritani, Mt. Dinnammare, Colle S. Rizzo, 38°13'03.9"N, 15°30'30.2"E, 450 m, seven young basidiomes growing with *Q.ilex*, *P.pinea*, and *R.pseudoacacia*, 14.10.2020, leg. & det. S. Saitta, TUR-A 209576 (GP); • Messina, Monti Peloritani, Mt. Dinnammare, Portella Armacera, 38°11'54.4"N, 15°29'28.9"E, 700 m, six young basidiomes growing with *Q.ilex* and *P.pinea*, 14.10.2020, leg. & det. S. Saitta, TUR-A 209577 (GP); • Ragusa, Monterosso Almo, Bosco Canalazzo, 37°05'12"N, 14°44'14"E, 690 m, four young to mature small basidiomes among grass in clearings, close to *Q.ilex*, *Q.pubescens* trees, 09.11.2016, leg. G. Vasquez, det. G. Simonini, GS10924; • Palermo Prov., Cinisi, Piano Margi, under *Q.ilex*, 06.11.2003, leg. G. Venturella, det. A. Biketova, SAF 1994 (GP); • Tuscany: Grosseto, Monte Argentario, Poggio Le Piane, Residence “il Mascherino”, 42°25'49"N, 11°10'28"E, 43 m, ten young to mature basidiomes, in sparse *Q.suber* forest, 30.10.1993, leg. & det. G. Simonini, GS1044; • Grosseto, Monte Argentario, Poggio Le Piane, road towards the Residence “il Mascherino”, 42°25'19"N, 11°09'55"E, 161 m, four large mature basidiomes, in the *Q.ilex* wood, 31.10.1993, leg. G. Nuccio, det. G. Simonini, GS1048; • Livorno, Collesalvetti, near Agriturismo Tenuta Vallelunga, 43°34'36"N, 10°23'45"E, 68 m, in a wood of *Q.cerris*, *Acer* sp., *O.carpinifolia*, and *F.ornus*, 03.10.2016, leg. M. Raumi, det. G. Simonini, GS10848 (GP); • Veneto: Camping Village “Cavallino”, Venezia, 45°27'24"N, 12°30'11"E, 3 m, four mature basidiomes growing among the grass and the needles, under *Pinuspinea*, 20.08.2009, leg. F. Golzio, det. G. Simonini, GS2475; RUSSIA • Krasnodarski Krai: Sochi City District, vic. of Mamedova Shchel Village, gorge of Lel River, 43°56'47"N, 39°18'23"E, mixed *Fagus-Carpinus-Quercus* forest along the river, on soil and litter, 31.08.2022, leg. T. Svetasheva, det. T. Svetasheva & E. Malysheva, LE F-332232 (GP); SERBIA • Central Serbia: Mačva District, Krupanj Municipality, Mala Gornja Village, near the peak of Mt. Kostajnik, 44°25'43"N, 19°17'16"E, 600 m, under *F.sylvatica* and *Q.petrea*, one basidiome, 02.08.2020, leg. A. Biketova & B. Dobrić, det. A. Biketova, K-M001435694 (AB B20-362, GP); SPAIN • Bizkaia: Laukiz, Talleri, 43°21'28"N, 2°55'10"W, 29 m, under *Q.robur*, in a roadside, 02.09.2005, leg. E. Fidalgo, det. J. Muñoz & A. Taylor, JAM0605 (GP); • Cantabria: Saja, Cambilla, 43°06'33"N, 4°16'14"W, 759 m, under *F.sylvatica*, in a roadside, 31.08.2005, leg. J. Muñoz, det. J. Muñoz & A. Taylor, JAM0547 (GP); • Ucieda, Río Bayones, 43°14'04"N, 4°13'01"W, 268 m, under *Q.robur*, growing among the grass in a cleared area, 06.09.2005, leg. J. Muñoz, det. J. Muñoz & A. Taylor, JAM0591 (GP); • Navarra: Lekunberri, Puerto de Uitzi, 43°03'01"N, 1°55'01"W, 850 m, under *F.sylvatica*, in a roadside, 09.09.2005, leg. M. Babace, J. Cadiñanos, E. Benguría, E. Fidalgo. A. Meléndez, M. Maguregui & J. Muñoz, det. J. Muñoz & A. Taylor, JAM0611 (GP); SWEDEN • Scania Co.: Lomma, Alnarpsparken, 55°39'04"N, 13°03'53"E, under *Quercus* sp., on clayey and base rich soil, 03.09.2000, leg. I. Goa, det. A. Taylor, LD 1213132 (GP); • Stockholm Co.: Stockholm, Ulriksdahl, growing in greenhouse, probably associated with *Tilia*, 16.04.2003, leg. & det. A. Taylor, AT2003001 (GP); • Västra Götaland Co.: Bohuslän, Hjälteby, Sundsbyvägen, Sundsby, 58°04'13.4"N, 11°41'22.5"E, in deciduous forest on shell gravel ground, 12.09.1994, leg. S. Jacobsson, det. A. Taylor, GB-0017989 (AT1994026, S. Jacobsson 94026; GP); UKRAINE • Vinnytsia Region: Vinnytsia Forest Park, one young basidiome growing among moss, under *F.sylvatica*, with presence of *P.sylvestris* and *C.betulus*, 07.09.2014, leg. & det. A. Biketova, K-M001435697 (AB B14-246; GP); UNITED KINGDOM • England: Berkshire, near Ascot, Silwood Park, 07.11.1996 and 29.08.2006, leg. A. Hills, det. A. Taylor, K-M000167946 (AH1996033; GP) and K-M000168799 (AH2006031; GP); • Oxfordshire, Bagley West Wood, sawmill area, 25.08.2004, leg. A. Hills, det. A. Biketova, K-M000167271 (AH2004100, GP); • Oxfordshire, Oxford, St Thomas the Martyr Churchyard, SP507603, with *Q.ilex*, 17.08.2004, leg. A.E. Hills, det. A. Hills & A. Taylor, K-M000167206 (AH2004079; GP); • Oxfordshire, near Wallingford, Oakley Wood, under *Q.robur*, 14.10.1998, leg. A.L. Warland, det. A. Hills & A. Taylor, K-M000170839 (AH1998100; GP); • Oxon, near Henley, Crowsley Park, 31.11.1996, leg. A. Hills, det. A. Hills & A. Taylor, AH1996031 (GP); • South Yorkshire, Sheffield, Norfolk Park, under *Q.cerris*, 08.08.1994, leg. C. Hobart, det. A. Taylor, AT1994006 (GP).

#### Notes.

*Hortiboletusengelii* is based on *Boletusengelii*Hlaváček 2001, which is, in turn, to be based on the earlier description of the invalid *Xerocomusquercinus* H. Engel & T. Brückn. nom. prov. The validating paper of J. Hlaváček (2001) is not free from mistakes; the new species is cited as a “nom. nov.”, whilst it is a “sp. nov.” and the earlier *X.quercinus* H. Engel & T. Brückn. nom. inval. (Engel and Brückner 1989; Engel et al. 1996) is cited as a basionym, on the type of which the new species had to be based. However, *X.quercinus* does not have any designated holotype, but only a list of findings. One of them is from the fungarium of Thomas Brückner, collected on an unknown date in 1989 in Thüringen, Gera, Weinberg, MTB 5138/1 and was chosen as the holotype by Hlaváček (2001). In this case, “MTB” is an abbreviation of “Messtischblatt”, which means “survey table sheet” or “topographical code” that was often used in Germany. Therefore, there is no voucher specimen indicated in the typification, but that is not compulsory, based on Art 40.6. and 40.7 of the ICN (Turland et al. 2018). Additionally, there is no evidence that Hlaváček ever studied the original material of *X.quercinus* himself. Despite all inconsistencies, the publication of *B.engelii*Hlaváček 2001 basically complies with the rules of the ICN (typification, diagnosis (Turland et al. 2018)) and, therefore, this name is valid and legitimate.

We attempted to find the type of *B.engelii* and locate the private collection of T. Brückner, but have not succeeded yet. We found out that the private collection of Heinz Engel was relocated to the Bavarian Natural History Collections (SNSB); however, none of the *X.quercinus* specimens listed in Engel and Brückner (1989) and Engel et al. (1996) is stored there. In Engel and Brückner (1989), there is a citation that the listed specimens were preserved not only in their private collections, but also in Edinburgh, likely referring to the herbarium of the Royal Botanic Garden Edinburgh (RBGE), because it was mentioned that they sent dry material to Roy Watling for verification. However, R. Watling and current curators of RBGE were unable to locate these specimens.

The use of the old name *Boletuscommunis* Bull. (Bulliard 1789) for the taxon, which we here describe under the name *H.engelii*, would be potentially possible (Redeuilh and Simonini 1999b) through the designation of a lectotype chosen among the basidiomes depicted in the original plate (pl. 393). However, there are several nomenclatural and taxonomic reasons which should be taken into consideration to discourage this alternative. As far as the nomenclatural reasons are concerned, we have to consider that J.B.F.P. Bulliard (1789), in a page of Herbier de la France (p. 328) belonging to a volume different from the one in which the original diagnosis of *B.communis* was firstly published, included the description and plates of *B.communis* under the newly-described species *Boletuschrysenteron*: “*Il faut aussi rapporter à cette espèce les individus dont nous avons donné la figure, pl. 393, sous le nom vulg. de BOLET COMMUN...*”. Furthermore, in the description of *B.chrysenteron*, the author explicitly cites again the plate 393 earlier referred to *B.communis*. Accordingly, part of the pl. 490 (including its caption) is referred to as *B.chrysenteron*, while in the description of *B.chrysenteron*, the French author cites both pl. 393 and pl. 490. In conclusion:

1. *Boletuscommunis* Bull. 1789 is validly and legitimately published, having both a potential lectotype (to be chosen among pictures in pl. 393) and description (caption of pl. 393).
2. Pl. 393 contains the potential lectotype of *B.communis*, but it might also contain a possible lectotype of *B.chrysenteron*.

Since both *B.communis* and *B.chrysenteron* were published before 1 Jan 1953, the uniqueness of the type does not invalidate either *B.communis* or *B.chrysenteron* (Art. 36.3 of the ICN (Turland et al. 2018)). Moreover, since *B.chrysenteron* is a valid, but illegitimate name (cf. also Hansen and Knudsen (1992) and Redeuilh and Simonini (1995)), choosing a shared lectotype (i.e. the basidiome in the lower right of pl. 393) for *B.communis* and *B.chrysenteron* could lead to their homotypic and indisputable synonymy and, consequently, to the use of the name *B.communis* as a substitute for the illegitimate name *B.chrysenteron*. Summing up, this is a nomenclatural case hard to unravel and potentially provoking nomenclatural instability in case of a proposal for a putative recombination of *Hortiboletuscommunis* in substitution of the later *H.engelii*, since the same epithet could be used for two different taxa.

From the taxonomic standpoint, we have to outline that the original plate of *B.communis* by Bulliard (pl. 393) could represent two different species, if not three; the five basidiomes labelled “B” (on the top) could correspond to *X.subtomentosus* (coarse interconnecting ribs are clearly visible on the upper half of the stipe of all individuals); three out of the four basidiomes labelled “A” (below, lower left and centre) look much like *H.bubalinus*, whereas the single basidiome on the lower right might well be an additional *H.bubalinus* (although with an unusual areolate pileus) or more probably a member of the *Xerocomelluschrysenteron*/*X.cisalpinus* complex. The pileus of a half basidiome labelled “C” (on the right side of the plate) could be any of the European xerocomoid fungi and it appears to be nearly impossible to identify. Accordingly, not one of the specimens reproduced in this plate can be considered an indisputable *H.engelii* as it is currently delimited and, thus, *B.communis* can only be doubtfully considered contaxic with the former species. Therefore, *B.communis* should be regarded as a *nomen confusum*.

The taxonomic status of the heterotypic synonym Boletussubtomentosussubsp.declivitatum (Martín 1904) is also in need of further clarification. The original plate of this subspecies, traditionally considered as conspecific with *H.engelii*, could well depict a mixture of different species. Elements 1 and 2 could be probably considered as *H.bubalinus*, while elements 6 and 7 are likely *H.rubellus*. The remaining sketches are unclear and very difficult to recognise. However, as it is the case for *B.communis*, it appears that there are no specimens in the original plate of B.subtomentosussubsp.declivitatum that can be linked with certainty to the current concept of *H.engelii* and, therefore, it should also be considered a *nomen confusum*. Anyway, being the epithet “*declivitatum*” proposed at the rank of variety, it does not compete with “*engelii*” at the rank of species.

*Hortiboletusengelii* belongs to the crown clade of *Hortiboletus* in our multilocus phylogenetic analysis (Fig. 1) as a well-supported (BS = 70, PP = 1.00) sister species of *H.hershenzoniae* (see below). They differ by 15 nucleotides and indel positions (3% dissimilarity). They both form a weakly-supported clade (multilocus: PP = 0.80) sister to the undescribed *Hortiboletus* sp. 1 from Pakistan (common clade in the multilocus tree: BS = 98, PP = 1.00).

### ﻿ Hortiboletus hershenzoniae

Taxon classificationFungiBoletalesBoletaceae

Biketova & Wasser sp. nov.

8E46EBF2-46B0-577C-92DE-839B7E71B820

MB847434

**Figure 13.**
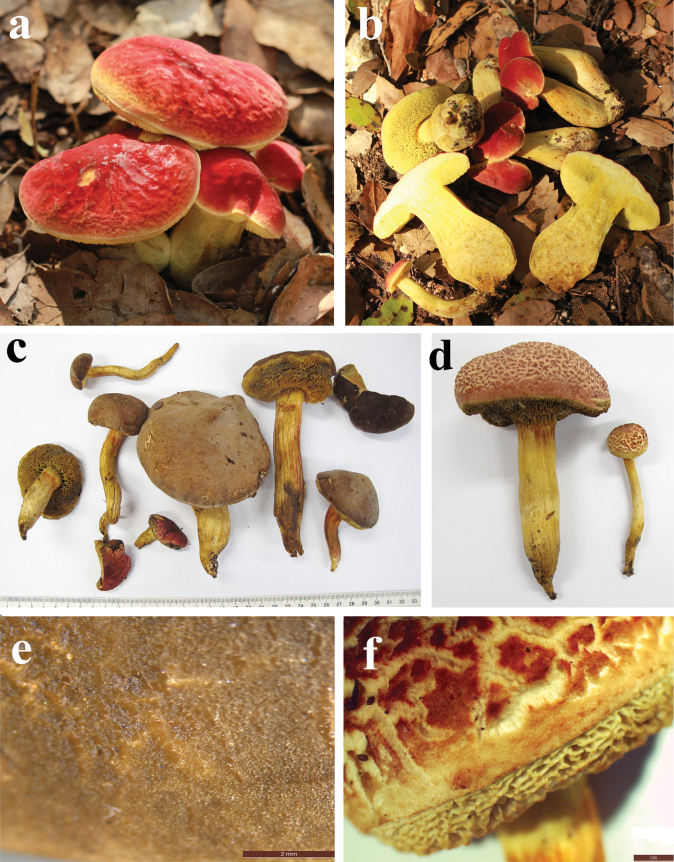
*Hortiboletushershenzoniae* basidiomes: **a, b** holotype collection K-M001435594; **c** K-M001435695; **d** K-M001435706; **e, f** pileus surface of collections: **e** K-M001435695 and **f** K-M001435706. Scale bars: 2 mm. Photos: (**a, b**) R. Kuznetsov; (**c–f**) A. Yu. Biketova.

**Figure 14.**
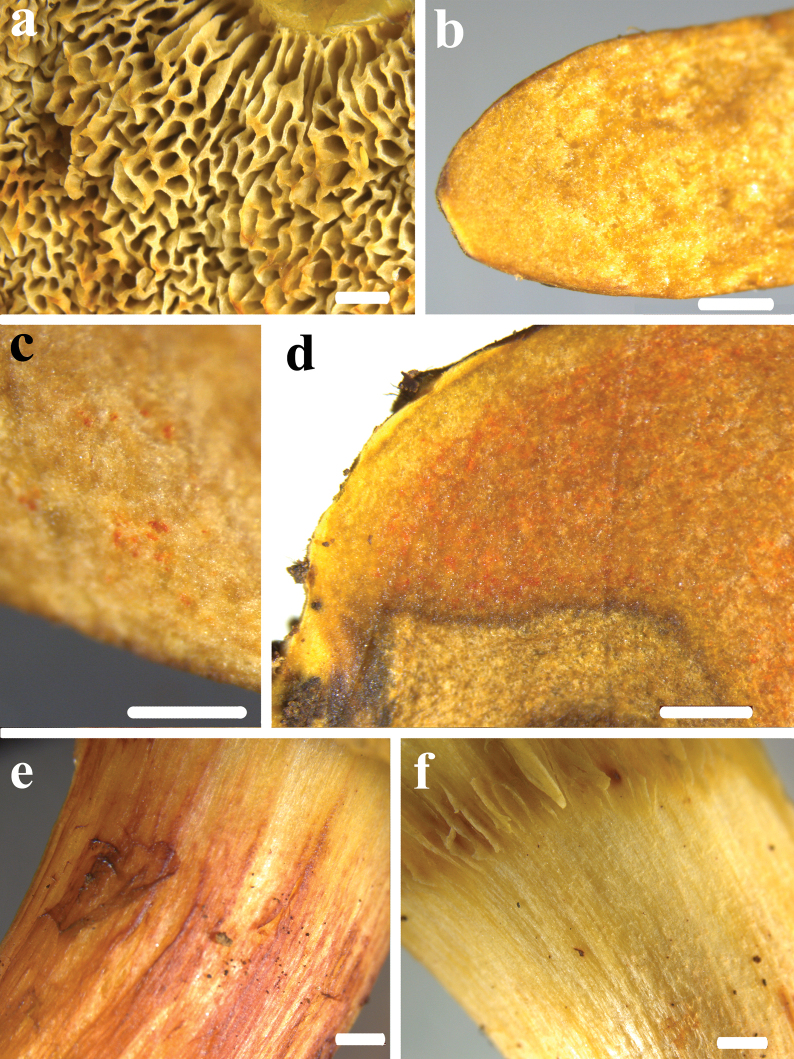
Details of macroscopical features of *H.hershenzoniae*: **a** uneven, denticulate hymenophoral surface of collection K-M001436710; **b–d** context of the stipe base: **b** without orange-red dots in a young basidiome (K-M001436710), **c** with sporadic orange-red dots in young basidiome (K-M001435695), and **d** collapsed and dispersed orange-red dots throughout the whole stipe base context of a mature basidiome (K-M001435695); **e** stipe surface with reddish fibrils on light-yellow ground in a brown-pileus basidiome of K-M001436710; **f** stipe surface with light-yellow ground in a red-pileus basidiome of K-M001435695. Scale bars: 2 mm (**a**, **e, f**); 1 mm (**b–d**). Photos: A. Yu. Biketova.

**Figure 15.**
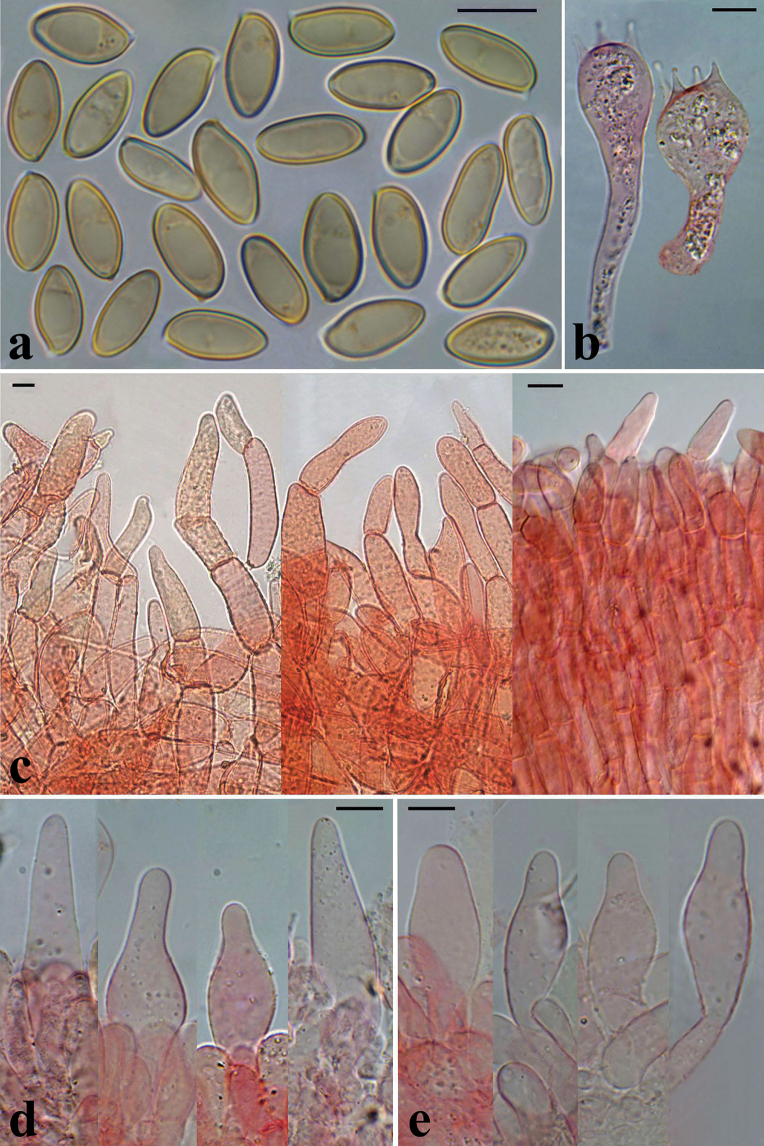
Microscopical features of *H.hershenzoniae*: **a** basidiospores of collection K-M001435693 in L4; **b** basidia in Congo red, left collection K-M001435710, right – K-M001435695; **c** pileipellis in Congo red, left collection K-M001435693, centre – K-M001435686, right – K-M001435594 (holotype); **d** pleurocystidia in Congo red, from left to right collections K-M001435710, K-M001435695 – twice, and K-M001435710; **e** cheilocystidia in Congo red of K-M001435710. Scale bar: 10 μm. Photos: G. Simonini.

Figs 3c, d , 8 , 13 , 14 , 15

#### Diagnosis.

Basidiomes pileate-stipitate, xerocomoid, epigeal, small to medium-small-sized. Young basidiomes usually have a red pileus and yellow stipe, but with age, the colour of the pileus can fade to various tints of brown or greyish-brown and the stipe surface develops a dark-red tint (red scales or fibrils on a yellow background). Pileus up to 10 cm in diam., dry, finely tomentose, sometimes alveolate-rugose or with small cracks. Tubes adnate to slightly decurrent, initially pale yellow to lemon yellow and becoming olivaceous-brown with age, slightly bluing when cut. Pores angular, uneven and denticulate in mature basidiomes, concolourous with tubes, only weakly bluing when bruised. Stipe up to 12 cm long, central or slightly off-centre, usually slender especially in young basidiomes, cylindrical, curved, somewhat tapering towards the base and slightly rooting. Context firm to soft in the pileus, fibrous and brittle in the stipe; pale yellow to lemon yellow, sometimes from pale pink to vinaceous underneath the pileus surface in old basidiomes, usually with minute dots of orange-red or vermilion-red pigment in the stipe base (sometimes dots are absent in young basidiomes); only erratically and slightly bluing when exposed to air. Basidiospores 11.4–13.9 × 6.0–6.6 µm, Qm = 2.02 ± 0.17, broadly elliptical to broadly fusiform, smooth. Pileipellis a palisadoderm, of long, parallel, smooth or sometimes encrusted hyphae; terminal elements 20.0–48.2 × 9.0–13.8 μm, mostly cylindrical and somewhat tapering towards the apex.

#### Etymology.

Named in honour of the late Zehara Avizohar-Hershenzon, who was one of the pioneers of the mycological science and investigations of macrofungi in Israel.

#### Holotype (MTB 10011371).

ISRAEL • Sharon Plain: Zikhron Ya’akov, 32°34'28"N, 34°58'33"E, under *Quercuscalliprinos*, 01.12.2012, leg. R. Kuznetsov, K-M001435594 (AB B12-071 – collector’s number), GenBank: ITS+LSU – PV030528, *tef1-α* – PV088300, and *rpb2* – PV088311.

#### Description.

***Basidiomes*** pileate-stipitate, xerocomoid, epigeal, small to medium-small-sized. Sometimes basidiomes grow fused together, forming one common pileus originating from two different stipes. ***Ontogenetic development*** gymnocarpic.

***Pileus*** 1.9–10 cm diam., initially nearly hemispherical, then convex, applanate to almost flattened, slightly depressed at centre, sometimes irregularly shaped, dry, finely tomentose, sometimes alveolate-rugose or with fine or coarse cracks especially in dry weather, with cream or yellowish (basidiomes with red pileus) or also vinaceous-red (basidiomes with brownish pileus) context in the cracks; pileus surface very variable in colour, initially with different tints of red (blood red, cherry red, rose red, vinaceous-red: BFF 41; OAC 600–605, 607, 621–623) and then usually slowly fading to various tints of brown or greyish-brown and ochraceous (hazel, clay buff, sepia, milky coffee, cigar brown, greyish-ochraceous: BFF 16, 17, 26- 8, 32; OAC 770, 771, 778, 736, 743, 745), especially in dry weather; cap margin slightly inrolled in young basidiomes and then straight, acute, sometimes obtuse gibbous-corrugate, somewhat (sometimes significantly) paler than the central part of the pileus in young basidiomes.

***Tubes*** up to 15 mm long, at first adnate, later emarginate or slightly decurrent with a tooth, initially straw yellow, lemon yellow or greenish-yellow (BFF 50, 54, 57, 64; OAC 1, 3, 896, 897), later greenish-yellow, citrine olivaceous to olive brown (OAC 801, 817, 832, 838, 894); slightly bluing when injured.

***Pores*** angular, uneven and denticulate in mature basidiomes, pale yellow, lemon or greenish-yellow (BFF 54, 57, 64; OAC 3, 856, 894, 896, 897) in young basidiomes and citrine olivaceous to olive brown with rusty tint (OAC 804, 848, 825, 839, 838, 785) with age, only weakly bluing when bruised.

***Stipe*** 4–11.5 × 0.7–3.7 cm, versiform, central or slightly off-centre, usually slender especially in young basidiomes, cylindrical, curved, somewhat tapering towards the base and slightly rooting, dry, initially smooth or slightly fibrillose and straw or lemon yellow throughout (BFF 50; OAC 1, 3, 853, 855, 856, 898) in wet weather, then becoming ornamented with vinaceous-red and brownish-red scales or fibrils (BFF 17, 19, 20; OAC 657, 635, 601, 602, 622) in the upper or middle part of the stipe and sometimes towards the base especially in dry conditions. ***Basal mycelium*** yellow or dirty yellow (OAC 1, 3-5, 853, 856, 847).

***Context*** firm to soft in the pileus, fibrous and brittle in the stipe; pale yellow, sulphur yellow to lemon yellow (BFF 55; OAC 1, 858), slowly fading on drying especially in the pileus, sometimes from pale pink to vinaceous (BFF 76, OAC 578, 599, 611) underneath the cuticle in old basidiomes, usually with minute dots of red-orange or orange-red (OAC 630, 649, 677) pigment in the stipe base that is mostly visible only under a stereomicroscope and sometimes absent in young basidiomes; sometimes only slightly bluing in the pileus and/or in the upper and middle part of the stipe (in wet weather) when exposed to air.

***Spore print*** olive brown (OAC 831, 832).

***Odour*** inconspicuous. ***Taste*** mild.

***Macrochemical spot-test reactions***: Melzer’s reagent: a weak fleeting-amyloid reaction on the hymenophoral trama and on the stipe and pileus context of fresh and dry basidiomes; 25% NH_4_OH: fading of pinkish pigment of the pileus context under cuticle; 10% FeSO_4_: olivaceous (BFF 62; OAC 866, 868, 832, 882) to bluish-grey on the context in the stipe base and pale olivaceous (OAC 850) to olivaceous-black (OAC 36, 37) on the pileus context; 10% H_2_SO_4_: sienna to light yellow ochre and (OAC 706, 707, 810–813) on pileus surface, yellow ochre (OAC 810–812) on pores, and olive-ochraceus to yellow ochre (OAC 803, 811, 818, 825) on the stipe context.

***Basidiospores*** [336/10/10] (10.8–)12.4 ± 0.9(–13.9) × (5.5–)6.2 ± 0.3(–6.6) µm, Qm = (1.90–)2.01 ± 0.08(–2.22), Vm = 251 ± 40 µm^3^, broadly elliptical to broadly fusiform, smooth, with a weak suprahilar depression in profile, yellowish ochraceous, with 1–3 large guttules when mature. Basidia 28–42 × 9–13 μm, generally four-spored, clavate, hyaline to yellowish, guttulate, without basal clamp.

***Basidia*** [51/2/2] (32.5–)48.4 ± 8.2(–74.0) × (11.8–)14.2 ± 1.9(–19.5) μm, subclavate to clavate, predominantly 4-spored, sterigmata (2.5–)3.2 ± 1.7(–6.7) μm long, hyaline to yellowish in 5% KOH and containing concolourous oil guttules, without basal clamps; basidioles subcylindrical to faintly clavate, smaller than basidia.

***Cheilocystidia*** [11/2/2] (45.0–)52.7 ± 6.3(–65.0) × (8.2–)12.4 ± 2.9(–16.2) μm, slender, ventricose-fusiform, often with elongated neck, with rounded tip, smooth, mostly hyaline to pale yellowish, some with intracellular yellowish oil content, in water and 5% KOH. ***Pleurocystidia*** [30/2/2] (38.0–)56.5 ± 8.1(–70.0) × (10.0–)13.6 ± 2.2(–19.5) μm, slender, ventricose-fusiform, often with elongated neck, with rounded tip, smooth, mostly hyaline to pale yellowish, some with intracellular yellowish oil content, in water and 5% KOH. ***Pseudocystidia*** not observed.

***Hymenophoral trama*** bilaterally divergent, intermediate between the “*Phylloporus*-type” and the “*Boletus*-type”: lateral strata consisting of poorly divergent, tightly arranged, non-gelatinous hyphae almost touching each other, hyaline to very pale yellow in water and 5% KOH; mediostratum consisting of tightly adpressed, non-gelatinous, parallel running hyphae; in Congo red, the mediostratum is slightly darker than the lateral strata.

***Pileipellis*** a palisadoderm of long, parallel, septate, smooth or encrusted (by granular pigment) hyphae; terminal elements [322/10/10] (25.2–)32.6 ± 5.9(–44.2) × (7.3–)10.9 ± 2.1(–13.9) μm, Qm = (2.32–)3.03 ± 0.43(–3.61), encrusted, mostly cylindrical and somewhat tapering towards the apex, obtusely triangular, acorn-shaped.

***Stipitipellis*** consisting of slender, subparallel, smooth walled, round tip, pale-yellow in 5% KOH hyphae, (3.0–)3.5–4.5(–6.5) μm broad. Bundles of a ***caulohymenium***, constituted of clavate ***caulobasidioles*** (23.0–)31.0–35.2(–42.5) × (8.0–)9.3–12.1(–14.5) μm, hyaline to pale yellow in 5% KOH are observed in the upper part of the stipe; fertile ***caulobasidia*** not observed. Confused, intermixed, sometimes ramified 4.4–6.0 μm hyphae from which caulobasidioles arise were observed, not constituting a true ***lateral stratum***.

***Stipe trama*** consisting of parallel running, (5.5–)6.7–7.1(–8.0) μm broad, sometimes restricted to septa hyphae, hyaline in 5% KOH, inamyloid; infrequent, versiform thromboplerous hyphae are observed here and there.

***Clamp connections*** absent in all tissues.

#### Ecology and phenology.

Solitary, in small groups, or caespitose, in Mediterranean thermophilous forests, growing with *Quercuscalliprinos* (Sleiman et al. (2021), as *H.engelii*, GP; pers. observ., GP), possibly also with *Q.ithaburensis* on calcareous soil, common in its habitat. There are some molecularly unconfirmed data referring to a putative association with *Pinushalepensis*. The main fruiting period starts after the first prolonged and heavy rains and goes from late October to December, sometimes continuing until the last rains in April.

#### Known distribution (see Fig. 5b).

So far known from Israel (this study, GP) and Lebanon (Sleiman et al. (2021), as *H.engelii*, GP). It likely grows in other countries of the eastern Mediterranean region.

#### Additional examined material.

ISRAEL • Carmel Mount: Mt. Carmel National Park, Nahal Oren valley, Henyon Ha’Agam, 32°43'26"N, 35°00'55"E, 260 m, under *Q.calliprinos*, four from young to mature basidiomes, 13.11.2012, leg. Y. Cherniavsky & O. Godorova, det. A. Biketova, K-M001435678 (AB B12-024, GP); • the same locality, two aged basidiomes, 17.11.2012, leg. O. Godorova & A. Biketova, det. A. Biketova, K-M001435686 (AB B12-046); • Mt. Carmel National Park, near the crossroad Damon, 32°44'03"N, 35°02'22"E, 505 m, under *Q.calliprinos*, several from young to mature basidiomes, 30.10.2015, leg. S. Tam, Y. Gruntman, N. Bar, Y. Segal, T. Schlesinger, E. Baum, Y. Ingel & A. Biketova, det. A. Biketova, K-M001436710 (AB B15-261; GP) and K-M001435695 (AB B15-263); • Dereh Nof HaCarmel, under *Q.calliprinos*, two mature basidiomes, 20.11.2014, leg. Y. Cherniavsky, det. A. Biketova, K-M001435698 (AB B14-217, GP); • ibid., under *Q.calliprinos*, 09.12.2014, three mature basidiomes (two infected by Hypomycescf.chrysospermus), leg. R. Kuznetsov, det. A. Biketova, K-M001435700 (AB B14-235); • Nesher, not far from the University of Haifa, near the road 672, close to the Cave, 32°45'23.1"N, 35°01'39.6"E, under *Pistacialentiscus* with *P.halepensis* in vicinity, one mature basidiome, 28.10.2015, leg. A. Biketova & A. Berqi, det. A. Biketova, K-M001435702 (AB B15-252); • Lower Galilee: HaSolelim Forest Reserve, near the intersection of highway 77 and the road to Hoshaya, 32°46'07"N, 35°17'38"E, in *Q.calliprinos* and *Q.ithaburensis* forest, one to two mature basidiomes in each specimen, 14.12.2012, leg. Z. Shafranov & A. Rotenberg, det. A. Biketova, K-M001435681 (AB B12-112), K-M001435683 (AB B12-113), K-M001435685 (AB B12-114), K-M001435687 (AB B12-117), and K-M001435689 (AB B12-118); • Sharon Plain: Zikhron Ya’akov, near Israel National Trail, 32°34'29"N, 34°58'33"E, under *Q.calliprinos*, one mature basidiome (infected by Hypomycescf.chrysospermus), 20.11.2014, leg. R. Kuznetsov, det. A. Biketova, K-M001435704 (AB B14-218, GP); • ibid., 32°34'28.5"N, 34°58'33.2"E, under *Q.calliprinos* and *Q.ithaburensis*, two mature basidiomes (one infected by Hypomycescf.chrysospermus), 07.04.2014, leg. R. Kuznetsov, det. A. Biketova, K-M001435703 (AB B14-189); • ibid., 32°34'13.0"N, 34°58'26.9"E, under *Q.calliprinos*, one mature basidiome, 05.12.2014, leg. R. Kuznetsov, det. A. Biketova, K-M001435708 (AB B14-228, GP); • Zikhron Ya’akov, near Israel National Trail, near the ravine, 32°34'29.3"N, 34°58'32.4"E, under *Q.calliprinos*, 05.12.2014, leg. R. Kuznetsov, det. A. Biketova, K-M001435706 (AB B14-227, GP, one mature and one aged basidiome) and K-M001435707 (AB B14-229, two mature basidiomes), respectively; • Zikhron Ya’akov, Ramat Menashe, near Israel National Trail, one immature basidiome, under *Q.calliprinos* and *Q.ithaburensis*, one very young basidiome, 10.01.2014, leg. R. Kuznetsov, det. A. Biketova, K-M001435696 (AB B14-238); • the forest near Zikhron Ya’akov, under *Q.calliprinos*, four young to mature basidiomes, 22.04.2015, leg. R. Kuznetsov & Y. Cherniavsky, det. A. Biketova, K-M001435710 (AB B15-248; GP); • Upper Galilee: Goren Park, 33°03'22"N, 35°13'48"E, under *Q.calliprinos*, two mature basidiomes, 01.12.2012, leg. Z. Shafranov, det. A. Biketova, K-M001435693 (AB B12-067); • ibid., under *Quercus* sp., one mature basidiome, 11.12.2014, leg. B. Gal, det. A. Biketova, K-M001435692 (AB B14-237); • Hanita Forest, 33°04'44"N, 35°09'57"E, 160 m, under *Q.calliprinos*, five mature to old basidiomes,18.05.2013, leg. Z. Shafranov, det. A. Biketova, K-M001435701 (AB B13-153); • Shomera, 33°04'47.1"N, 35°16'45.3"E, under *Q.calliprinos*, near *P.lentiscus* and *Laurusnobilis*, one mature basidiome and two primordia, 26.10.2015, leg. Y. Segal, det. A. Biketova, K-M001435699 (AB B15-260, GP).

#### Notes.

In the present study, a new species, *H.hershenzoniae* is described. It is very similar to *H.engelii* and *H.rubellus*, but well distinguished from them based on morphology, phylogeny (Fig. 1), ecology, and distribution (Figs 4, 5). Basidiomes of these three species are very variable in colour. Young specimens of *H.hershenzoniae* usually have a red pileus and yellow stipe, but with age, especially in dry conditions, the colour of the pileus usually fades to various tints of brown or greyish-brown and the stipe surface develops a dark-red tint (red scales or fibrils). Such transition of red pigments causes a high chromatic variation, which is also a common feature in *H.engelii*. We can call this phenomenon “migration of red pigments from the pileus surface to the stipe surface”. In contrast, *H.rubellus* usually has a red-coloured stipe surface even in young basidiomes with red pileus, which is not observed in the specimens of *H.hershenzoniae*. *Hortiboletushershenzoniae* has orange-red or vermilion-red dots in the context of the stipe base, but they are mostly visible only under a stereomicroscope or are totally absent in young basidiomes (see Figs 13b, 14b–d), unlike the clearly visible vermilion-red dots of *H.engelii* and *H.rubellus* (see Figs 6h, 11h) (Engel et al. 1996; Taylor et al. 2002; Hills 2008; Muñoz et al. 2008).

The basidiospores of *H.hershenzoniae* are significantly broader (average width 6.2 μm, Qm = 2.01) than those of *H.engelii* (average width 5.1 μm, Qm = 2.35), *H.rubellus* (average width 5.0 μm, Qm = 2.31), and *H.bubalinus* (average width 4.8 μm, Qm = 2.46) (Fig. 8a). Terminal elements of the pileipellis are encrusted, mainly cylindrical, somewhat tapering towards the apex, and sometimes obtusely triangular. Their size and shape are more similar to those of *H.engelii* (Fig. 8b). The current phylogenetic analysis shows that *H.hershenzoniae* clusters in a strongly-supported clade in our multilocus analysis (BS = 91; PP = 1.00) and it is closely related to *H.engelii* (see above and Fig. 1). Sleiman et al. (2021) reported this species in Lebanon under the name *H.engelii* (T1-1). Based on a BLASTn analysis of the ITS sequence MZ088076 with those of our Israeli specimens (99.6–99.9% similarity), as well as on the current phylogenetic reconstruction, the Lebanese specimen unequivocally belongs to *H.hershenzoniae*.

##### ﻿Key to the European and Levantine species of *Hortiboletus*

**Table:** 

1	Pileus variable in colour, often showing a paler, faded marginal zone; context in the pileus and upper part of the stipe usually turning intensely blue when cut and often contrasting with an innate flesh pink hue underneath pileus surface; orange-red dots in stipe base context usually absent, if present sporadic, never abundant; basidiospores with an average length/width ratio Qm ≥ 2.5; pileipellis terminal (20.6–36.8 × 10.1–16.3 μm, Qm = 2.31 ± 0.66) and subterminal elements rather short and swollen, moderately to strongly encrusted	***H.bubalinus***
–(2)	Pileus variable in colour, but pigments usually distributed evenly on the surface; context in the pileus and upper part of the stipe turning blue or nearly unchanging when cut, without (or only occasionally) with an innate flesh-pink hue underneath pileus surface; orange-red dots in stipe base context evident and abundant; basidiospores with an average length/width ratio Qm < 2.4; pileipellis terminal elements mainly cylindrical or cystidioid	**2**
2	Pileus and at least the lower half of stipe surface bright red in young specimens, retaining this tint over time or fading when mature; pileipellis terminal elements in mature specimens generally smooth	***H.rubellus***
–(3)	Pileus and stipe surface never red simultaneously; stipe never red at the base, but sometimes becoming reddish in the upper part at maturity; pileipellis terminal elements in mature specimens weakly to prominently encrusted	**3**
3	Basidiospores with an average length/width ratio higher than 2.2; if lower or equal, then average width lower than 6.0 μm; widely distributed in Europe and also occurring in Pakistan and the USA	***H.engelii***
–	Basidiospores with an average length/width ratio lower than or equal to 2.2; growing in association with thermophilic oaks (*Quercuscalliprinos*, *Q.ithaburensis*) in the Mediterranean area; presently known only from Israel and Lebanon	***H.hershenzoniae***

##### ﻿Extralimital taxa

Species presented in this section are from North America and their study was not included in the main tasks of our current research.

### ﻿ Hortiboletus campestris

Taxon classificationFungiBoletalesBoletaceae

(A.H. Sm. & Thiers) Biketova & Wasser, Index Fungorum 257: 1 (2015)

ADA1667F-73B9-5CA3-BC67-2307DEF5D194

MB551543

≡ Boletuscampestris A.H. Sm. & Thiers, Boletes of Michigan: 266 (1971)
= Boletusharrisonii A.H. Sm. & Thiers, Boletes of Michigan: 267 (1971)

#### Examined material.

USA • Michigan: Washtenaw Co., Ann Arbor, 42°16'31"N, 83°43'51"W, gregarious on lawn, 16.07.1966, leg. & det. A.H. Smith, MICH4999 (A.H. Smith 72961 – collector’s number, holotype), GenBank: ITS – PV036230, LSU – PV036235, *tef1-α* – PV106168, and *rpb2* – PV106172; • Washtenaw Co., Ann Arbor, 42°16'31"N, 83°43'51"W, gregarious on lawn near *Picea* sp., 01.07.1967, leg. K.A. Harrison, det. A.H. Smith, MICH10011 (K.A. Harrison 9504 – collector’s number, holotype of *Boletusharrisonii*), GenBank: ITS – PV036229, LSU – PV036234, *tef1-α* – PV106169, and *rpb2* – PV106173).

#### Notes.

Conspecificity of *H.campestris* and *B.harrisonii* was proven by phylogenetic multilocus and four single-locus analyses of their holotypes MICH4999 and MICH10011, respectively (Figs 1, 2). In the phylogenomic tree of Tremble et al. (2024), they also cluster together in one species-level clade. In the current multigene phylogeny, *H.campestris* occupies a rather isolated position in the crown clade of *Hortiboletus* and forms a strongly-supported terminal clade (BS = 100; PP = 1.00).

There is a lot of confusion in field recognition and identification of this species. Based on the sequence data from INSDC and connected collection data from publications (Frank et al. 2020; Kuo and Ortiz-Santana 2020), as well as iNaturalist (2024), all specimens identified as *H.campestris* (MICH KUO-08240502 (based on the *tef1-α* sequence MK721094 and *rpb2* – MK766302), F:PRL5991MAN, F:PRL5879MAN, and iNaturalist 66900805), *Hortiboletus* sp. ‘*campestris*’ (DD614), or *B.harrisonii* (MICH KUO-09071204 and PBM4097) actually belong to *Hortiboletusflavorubellus* (see below). Moreover, the LSU sequence of the MICH KUO-08240502 collection identified as *H.campestris*, actually belongs to another collection and species – *Xerocomustenax* Nuhn & Halling. Additionally, there are no *H.campestris* collections represented by their ITS sequences in INSDC, which are correctly identified to the species level (see Fig. 1 and Suppl. materials 1, 3). Further studies on the morphology and distribution of *H.campestris* are really needed.

### ﻿ Hortiboletus flavorubellus

Taxon classificationFungiBoletalesBoletaceae

(Thiers & A.H. Sm.) Biketova & Tremble comb. nov.

4B36CC8C-44EF-51D3-AE6F-8623EFAFABC0

MB855845

Boletus flavorubellus Thiers & A.H. Sm., Michigan Bot. **5**(3): 117 (1966). Basionym.
= Boletusrubellusvar.flammeus A.H. Sm. & Thiers, Boletes of Michigan: 269 (1971)
? = Boletussubfraternus Coker & Beers ex Meyers & Van T. Cotter, Index Fungorum **412**: 1 (2019).

#### Material examined.

USA • Michigan: Washtenaw Co., Ann Arbor, 42°16'31"N, 83°43'51"W, on wet soil in woods, 11.08.1960, leg. & det. A.H. Smith, MICH10015 (A.H. Smith 62872 – collector’s number, holotype), GenBank: ITS – PV036233, LSU – PV036238, *tef1-α* – PV106170, and *rpb2* – PV106174; • Washtenaw Co., west of Ann Arbor, Saginaw Forest on Liberty Road, 42°16'12"N, 83°48'25"W, scattered on humus in mixed forest of *Quercus* sp. and *Pinus* sp., 02.08.1966, leg. & det. A.H. Smith, MICH10035 (A.H. Smith 73026, holotype of Boletusrubellusvar.flammeus), GenBank: ITS – PV036231 and PV036232, LSU – PV036236 and PV036237, *tef1-α* – PV106171, and *rpb2* – PV106175.

#### Notes.

Present phylogenetic analysis, which included sequences of holotypes of *B.flavorubellus* MICH10015 and B.rubellusvar.flammeus MICH10035, proved that these two taxa are conspecific and allowed us to propose a new taxonomic combination *Hortiboletusflavorubellus*. These two type specimens also cluster together in the same species-level clade in the phylogenomic tree of Tremble et al. (2024). In the current multigene phylogeny, *H.flavorubellus* forms a strongly-supported terminal clade (BS = 100, PP = 1.00), sister to the *H.engelii*/*H.hershenzoniae*/*Hortiboletus* sp. 1 subclade (BS = 85, PP = 1.00).

This species, described by Thiers and Smith in 1966, is not yet understood by both mycologists and citizen scientists. Based on the sequence data from INSDC, connected publications (Nuhn et al. 2013; Frank et al. 2020; Kuo and Ortiz-Santana 2020), as well as records in iNaturalist (2024) and Mushroom Observer (2024), there are no collections identified either as *B.flavorubellus* or B.rubellusvar.flammeus (see Fig. 1 and Suppl. materials 1, 3). A few of the collections are identified as *H.rubellus* (e.g. FLAS-F-60513) or Xerocomelluscf.rubellus (MB03-033), a synonym of which B.rubellusvar.flammeus is considered, based on Index Fungorum (2025). However, most of *H.flavorubellus* collections were either not identified to the species level or misidentified as *H.campestris* (e.g. iNaturalist 66900805), *B.harrisonii* (e.g. PBM4097), and *B.subfraternus* (MICH KUO-08101302) (see Fig. 1 and Suppl. materials 1, 3). However, there is a possibility that *B.subfraternus* is the actual synonym of this species; therefore, it is necessary to conduct its type study, including analysis of their DNA sequences.

## ﻿Discussion

### ﻿Tandem repeat insertions in ITS region

Minisatellite-like insertions within the ITS region are rare in fungi, but have previously been observed in the *Boletaceae* subfamily *Leccinoideae*, particularly in *Leccinum* – 240–749 bp (both ITS1 and ITS2 regions, den Bakker et al. (2004)), *Octaviania* (ITS2 region, Orihara et al. (2012)), *Rossbeevera* – 199–366 bp (ITS2 region), and *Turmalinea* – 272–603 bp (ITS2 region, Orihara et al. (2016)). Here, such insertions were first reported not only in the ITS region in *Hortiboletus*, but also in the entire subfamily *Boletoideae*. It is possible that minisatellite-like repeats are present in other *Boletaceae* genera as well, but they have not been detected and characterised yet.

Short microsatellite AC repeats (up to 26 bp) observed in ITS1 region have especially high variability in *H.rubellus* and *H.bubalinus*, with intraspecific and intrageneric difference in length up to 12 bp. Moreover, the ITS2 region of ten *Hortiboletus* species has long tandem repeat (minisatellite) insertion ranging from (103) 133 to 253 bp with 2.0–3.2(–5.3) copy number of repeats (see Table 1).

Similar to *Rossbeevera* and *Turmalinea* (Orihara et al. 2016), minisatellite-like insertions in the ITS2 region of *Hortiboletus* spp. are highly conserved within each species ((95–)97–100% similarity), but divergent between species, which is a result of concerted evolution. Using a relatively small fragment of DNA sequence with this insertion, for BLASTn search against INSDC and UNITE databases, helps significantly reduce the number of “sequences producing significant alignments” to those that belong only to the target species and closely-related species of *Hortiboletus*. Therefore, those minisatellite-like insertions are very informative and can, thus, be used efficiently for DNA barcoding.

Due to frequent intragenomic polymorphisms especially within these insertions, the ITS region of both *H.rubellus* and *H.bubalinus* has been difficult to sequence with the classical Sanger method and cloning has been performed to obtain full ITS sequences of some collections in the current study.

Den Bakker et al. (2004) conducted TRF analysis of the ITS region of *Leccinum* using default parameters with alignment settings 2-7-7 (match - mismatch - indels), while Orihara et al. (2016) used alignment settings 2-3-5 for analysis in *Rossbeevera* and *Turmalinea*. In the present study, we ran TRF analysis of each subset of sequences using various alignment settings and gave results only of 2-7-7 and 2-3-5 settings in Table 1 as the most distinct. In most cases, results (including the core sequence, period size of repeats, and the length of detected insert) differ depending on the alignment settings. These settings characterise weights for match, mismatch and indels – with lower weights allowing alignments with more mismatches and indels. In summary, it is important to test each set of sequences with a range of alignment parameters in TRF analysis for more comprehensive evaluation of tandem repeats.

### ﻿Phylogeny, taxonomy, morphology and geography

According to Nuhn et al. (2013), in their inclusive multilocus phylogenetic analysis of the family *Boletaceae*, the “rubellus” clade is nested in the “anaxoboletus group”, which is congruent to the major clade of the subfamily *Boletoideae* as proposed in the study by Wu et al. (2014). Our analyses also revealed the obvious separation of the genus *Hortiboletus* from the morphologically closely related *Xerocomellus* and *Imleria* (Figs 1, 2).

In the present study, we have performed the most comprehensive molecular phylogenetic reconstruction of the genus *Hortiboletus* with the inclusion of 168 new sequences by using four genetic markers. It forms a strongly-supported generic clade (multilocus: BS = 100, PP = 1.0) and includes 24 to 25 different species-level clades, among which are 17 previously described, one newly described and from six to seven undescribed species (Fig. 1).

We clarified the position of *H.rubroreticulatus* that has not been fully resolved in Pham et al. (2024). It forms the most basal clade of *Hortiboletus* in the multilocus (BS = 100, PP = 1.00), ITS and *tef1-α* phylogenies, but clusters between two distinct *Xerocomellus* lineages in the LSU phylogeny. The next basal clade represents *H.rupicapreus* in the multilocus and ITS trees (both: BS = 100, PP = 1.00), which is also rather distant to other *Hortiboletus* species. However, in LSU, *tef1-α*, and *rpb2* trees, *H.campestris* occupies this basal position (all: BS = 100, PP = 1.00). The further basal branch (multilocus: BS = 99, PP = 1.00) belongs to *H.bubalinus*, *H.arduinus*, and, potentially, three to four unknown species. In *tef1-α* phylogeny, *H.napaeus* clusters together with this branch with weak statistical support (BS = 57, PP = 0.89). The rest of species cluster in the main crown clade (multilocus: no statistical support; ITS: BS = 94, PP = 1.00) with minor differences in the topology of branches between multilocus and single-gene trees mainly due to the lack of some sequenced loci for several species and also due to variations in species delimitation resolution, as well as number and percentage of parsimony informative sites between loci.

The geographical distribution of the 18 *Hortiboletus* species, currently known worldwide, includes the following continents: Europe (3 species), Asia (14 species), Australia (1 species), and North America (5 species). Most of the taxa appear to be restricted to a single continent, except of the three predominantly European species: *H.rubellus* which is also known from Asia (Abkhazia*, GP; Armenia, Azerbaijan, and Turkey, not GP), *H.engelii* which is also known from Asia (Pakistan) and North America (USA, based on a single genetically proven collection that was likely introduced there: https://www.inaturalist.org/observations/187446512), as well as *H.bubalinus* which has been recorded in Asia (Turkey, not GP) and North America (USA), but also in New Zealand, where this species was introduced (Figs 4, 5). To better understand the diversity, distribution patterns, ecological requirements, host specificity as well as taxonomy and phylogeny of the genus *Hortiboletus*, more extensive research will be necessary on continental as well as on a global scale in the future.

The main distinguishing morphological, ecological, and biogeographic characters of the four target species from Europe and Levant are summarised in the Table 2.

**Table 2.** Main distinguishing characters of the studied *Hortiboletus* species.

Character	*H.bubalinus*	*H.engelii*	*H.hershenzoniae*	*H.rubellus*
Pileus colour	Very variable in colour; from bright-red or dark-red tones (garnet, cherry, beetroot) to beige, cappuccino colour, ocher, chamois olive; most often firstly reddish-brown, but typically paler towards margin, later usually fading to drab tinges, somewhat ochraceous buff with pinkish patches.	Extremely variable colours, generally dark brown when young, later fading to ochraceous, buff, olivaceous brown, but also various shades of red (pinkish-red, orange-red, reddish-brown etc); sometimes persistently carmine red in all developmental stages	Various shades of red when young, later fading brown, greyish-brown or ochraceous, but sometimes retaining reddish colours	Usually, with bright-red tints, blood red, wine red, cherry red, rose-red, orange red, pink, fading or not with age
Pileus surface	Markedly wrinkled-rugulose and finely pruinose when young, usually not cracked or occasionally areolate towards the peripheral zone	Finely tomentose to sometimes rugulose, later finely areolate towards the peripheral zone or with minute to coarse cracks with dry weather	Finely tomentose, sometimes alveolate-rugulose or with small cracks with dry weather	Finely tomentose to sometimes rugulose when young, but later smooth, sometimes areolate towards the peripheral zone
Stipe colour	Variable, mostly with pinkish-reddish-brown tones, but yellow or sometimes reddish at very apex; as a rule reddish tones tend to disappear with age	Usually reddish on an ochraceous yellow background or concolourous with it; sometimes entirely yellow or red, but never red at the base.	Straw or lemon yellow throughout when young, later dark red due to red scales or fibrils on a yellow background (similar to those in *H.engelii*)	Usually red over most of the surface or at least in the lower part, yellow at apex; sometimes entirely yellow or red.
Context colour and colour change on exposure	Pale yellow, but dirty ochraceous yellow in the lower half of the stipe; in young specimens, flesh pink for a few mm underneath pileus surface; turning light blue above the tubes and also bluing in the peripheral layers of the stipe	Pale yellow, but dirty ochraceous yellow in the lower half of the stipe; usually turning very light blue, mostly in the pileus-stipe connection zone, above the tubes and in the peripheral layers of the stipe	Pale yellow, sulphur yellow to lemon yellow; usually only slightly bluing in the pileus and/or in the upper and middle part of the stipe (wet weather)	Pale yellow, but dirty ochraceous-yellow in the lower half of the stipe; turning light blue (sometimes rather intensive), especially in the pileus-stipe connection zone, peripheral layers of the stipe, and above the tubes
Orange-red dots in the stipe base context	Usually absent or rarely present, but then scattered.	Present, scattered to abundant	Present in mature specimens, scattered and minute, usually visible under stereomicroscope	Present, usually clearly visible, often abundant.
Average spore size (μm), Qm, Vm (μm^3^)	11.7 ± 0.6 × 4.8 ± 0.2, Qm = 2.46 ± 0.10, Vm = 140 ± 20	12.1 ± 0.8 × 5.1 ± 0.3, Qm = 2.35 ± 0.13, Vm = 171 ± 30	12.4 ± 0.9 × 6.2 ± 0.3, Qm = 2.01 ± 0.08, Vm = 251 ± 40	11.6 ± 0.6 × 5.0 ± 0.2, Qm = 2.31 ± 0.12, Vm = 155 ± 17
Pileipellis terminal elements; shape, size (μm), Qm	Variable, usually more or less swollen, mainly bullet-shaped or acorn-shaped, sometimes subglobose; moderately to strongly encrusted; 28.7 ± 8.1 × 13.2 ± 3.1, Qm = 2.31 ± 0.66	Mainly broadly cylindrical with rounded apex to cystidioid, sometimes also with elongated neck; finely encrusted, more intensely downwards; 36.5 ± 11.0 × 11.3 ± 2.2, Qm = 3.47 ± 1.58	Mainly cylindrical and somewhat tapering towards the apex, encrusted; 32.6 ± 5.9 × 10.9 ± 2.1, Qm = 3.03 ± 0.43	Mainly broadly cylindrical with rounded apex to cystidioid, sometimes also with elongated neck; not encrusted (especially in young specimens) or poorly and finely encrusted; 25.6 ± 4.2 × 8.6 ± 1.7, Qm = 3.12 ± 0.55
Ectomycorrhizal association (based on GP specimens)	Preferably *Tilia*, *Populus*, *Quercus*, *Fagus*, sometimes, *Betula*, *Carpinus*, *Corylus*, *Castanea*, *Carya*, *Picea*, *Pinus*, also in mixed forests with *Cedrus*, *Larix*, *Salix*, and *Alnus*	Preferably with *Quercus*, *Fagus*, *Tilia*, *Carpinus*, sometimes *Betula*, *Castanea*, *Corylus*, *Halimium*, *Populus*, *Pinus*, and in mixed forests with *Picea*	Thermophilic evergreen *Quercus* species – *Q.calliprinos*	Preferably with *Quercus*, *Fagus* and *Tilia*, but also in mixed forests with *Castanea*, *Betula*, *Carpinus*, *Corylus*, and *Pinus*
Distribution (based on GP specimens)	Europe, USA, and New Zealand	Europe, Pakistan, and USA	Levant (Israel and Lebanon)	Europe and South Caucasus

## Supplementary Material

XML Treatment for Hortiboletus

XML Treatment for Hortiboletus rubellus

XML Treatment for Hortiboletus bubalinus

XML Treatment for Hortiboletus engelii

XML Treatment for Hortiboletus hershenzoniae

XML Treatment for Hortiboletus campestris

XML Treatment for Hortiboletus flavorubellus

## ﻿Additional information

### Adherence to national and international regulations

All the fungal strains used in this study have been legally obtained, respecting the Convention on Biological Diversity (Rio Convention).

### Author ORCIDs

Alona Yu. Biketova https://orcid.org/0000-0003-0801-0660

Tatyana Yu. Svetasheva https://orcid.org/0000-0001-8968-9518

Andy F. S. Taylor https://orcid.org/0000-0003-0727-8171

Giampaolo Simonini https://orcid.org/0009-0006-1695-1443

Matteo Gelardi https://orcid.org/0009-0008-0903-852X

Olga V. Morozova https://orcid.org/0000-0002-7329-528X

Elias Polemis https://orcid.org/0000-0002-2728-7350

José A. Muñoz https://orcid.org/0009-0009-8445-263X

László Albert https://orcid.org/0000-0003-4505-3010

Salvatore Saitta https://orcid.org/0000-0003-0185-8765

Solomon P. Wasser https://orcid.org/0009-0009-3521-2576

Eviatar Nevo https://orcid.org/0000-0002-4884-2499

Georgios I. Zervakis https://orcid.org/0000-0002-2892-098X

Alfredo Vizzini https://orcid.org/0000-0001-8390-6446

Bálint Dima https://orcid.org/0000-0003-2099-3903

### Data availability

All of the data that support the findings of this study are available in the main text or Supplementary Information.

## Supplementary materials

Supplementary material 1 Specimens used in molecular phylogenetic studies and their INSDC and UNITE accession numbers Data type docx Explanation note **table S1.** Specimens used in molecular phylogenetic studies and their INSDC and UNITE accession numbers. Newly-generated sequences are in bold. Sequences submitted in public repositories, but not used in phylogeny are marked with asterisk (*). https://binary.pensoft.net/file/1351700This dataset is made available under the Open Database License (http://opendatacommons.org/licenses/odbl/1.0/). The Open Database License (ODbL) is a license agreement intended to allow users to freely share, modify, and use this Dataset while maintaining this same freedom for others, provided that the original source and author(s) are credited.Alona Yu. Biketova, Tatyana Yu. Svetasheva, Andy F. S. Taylor, Giampaolo Simonini, Matteo Gelardi, Olga V. Morozova, Elias Polemis, José A. Muñoz, László Albert, Salvatore Saitta, Solomon P. Wasser, Eviatar Nevo, Georgios I. Zervakis, Alfredo Vizzini, Bálint Dima

Supplementary material 2 Aligned and concatenated dataset of *Hortiboletus* sequences (ITS, LSU, *tef1*-α, *rpb2*) and closely-related genera used for phylogenetic reconstruction Data type fas https://binary.pensoft.net/file/1351701This dataset is made available under the Open Database License (http://opendatacommons.org/licenses/odbl/1.0/). The Open Database License (ODbL) is a license agreement intended to allow users to freely share, modify, and use this Dataset while maintaining this same freedom for others, provided that the original source and author(s) are credited.Alona Yu. Biketova, Tatyana Yu. Svetasheva, Andy F. S. Taylor, Giampaolo Simonini, Matteo Gelardi, Olga V. Morozova, Elias Polemis, José A. Muñoz, László Albert, Salvatore Saitta, Solomon P. Wasser, Eviatar Nevo, Georgios I. Zervakis, Alfredo Vizzini, Bálint Dima

Supplementary material 3 Non-collapsed ML and BI phylogenetic trees: ITS, LSU, *tef1*-α, and *rpb2* Data type pdf https://binary.pensoft.net/file/1351702This dataset is made available under the Open Database License (http://opendatacommons.org/licenses/odbl/1.0/). The Open Database License (ODbL) is a license agreement intended to allow users to freely share, modify, and use this Dataset while maintaining this same freedom for others, provided that the original source and author(s) are credited.Alona Yu. Biketova, Tatyana Yu. Svetasheva, Andy F. S. Taylor, Giampaolo Simonini, Matteo Gelardi, Olga V. Morozova, Elias Polemis, José A. Muñoz, László Albert, Salvatore Saitta, Solomon P. Wasser, Eviatar Nevo, Georgios I. Zervakis, Alfredo Vizzini, Bálint Dima
