# Supplementary material for: ﻿Morphological and molecular re-assessment of European and Levantine species of the genus Hortiboletus (Boletaceae)
Source: IMA Fungus. 2025 Jun 12;16:e144731. doi: 10.3897/imafungus.16.144731 (PMC12179652; doi:10.3897/imafungus.16.144731)
Supplement: Supplementary material 1 — Specimens used in molecular phylogenetic studies and their INSDC and UNITE accession numbers [file imafungus-16-e144731-s001.docx]

**Supplementary Data**

**Table S**1. Specimens used in molecular phylogenetic studies and their GenBank and UNITE accession numbers. Newly generated sequences are in bold. Sequences submitted in public repositories, but not used in phylogeny are marked with asterisk (*).

| **Taxon** | **Isolate/voucher /strain** | **Country (locality & coordinates are for the map)** | **Host plants & habitat** | **Accession number** | | | | **Notes** | **References** |
| --- | --- | --- | --- | --- | --- | --- | --- | --- | --- |
|  |  |  |  | **ITS** | **LSU** | ***tef*1-α** | ***rpb*2** |  |  |
| *Hortiboletus amygdalinus* | HKAS 54166 (Z.L. Yang 5070) | China: Yunnan | Unknown | **-** | NG_059622 | KT990777 | KT990416 | holotype | Wu et al. 2016 |
| *Hortiboletus amygdalinus* | HKAS 54242 (Z.W. Ge 2046) | China: Yunnan | Unknown | **-** | KT990580 | KT990776 | KT990415 | paratype | Wu et al. 2016 |
| *Hortiboletus amygdalinus* (*Hortoboletus* sp.) | TN201911 | Japan: Tokyo | Unknown | LC832089 | **-** | **-** | **-** | **-** | Okabe et al, unpubl. |
| *Hortiboletus amygdalinus* | NIBRFG0000502792 | South Korea: Daecheongdo Island | Unknown | MW578955 | MW578962 | **-** | **-** | **-** | Kim et al. 2021 |
| *Hortiboletus amygdalinus* (as *Xerocomus* sp.) | SFC20120725-43 | South Korea: Mt. Oseo | Unknown | KJ609174 | **-** | **-** | **-** | **-** | Lee et al. 2014 |
| *Hortiboletus arduinus* | FHMU3323 | China: Zhejiang | Unknown | **-** | MT646432 | MT646447 | **-** | holotype | Xie et al. 2020 |
| *Hortiboletus arduinus* | FHMU3324 | China: Zhejiang | Unknown | **-** | MT646439 | MT646448 | **-** | paratype | Xie et al. 2020 |
| *Hortiboletus arduinus* (as *Xerocomellus* sp.) | HKAS 76673 | China | Unknown | **-** | KF112370 | KF112182 | KF112693 | **-** | Wu et al. 2014 |
| *Hortiboletus arduinus* | HMJAU68182 (W3104) | China | Unknown | **-** | OR704333 | OR663931 | OR573951 | **-** | Wang et al. 2024 |
| *Hortiboletus arduinus* | HMJAU68183 (W3105) | China | Unknown | **-** | OR704334 | OR663932 | OR573952 | **-** | Wang et al. 2024 |
| ***Hortiboletus bubalinus*** | **IB1997886** | **Austria: Tyrol** | ***Betula* and *Corylus*** | - | **PV061847** | - | - | - | **This study** |
| ***Hortiboletus bubalinus*** | **H6032689** | **Finland: Nylandia** | ***Quercus robur*** | **PV094861** | - | ~~-~~ | ~~-~~ | - | **This study** |
| ***Hortiboletus bubalinus*** | **H6036888** | **Finland: Nylandia** | ***Tilia ×* *europaea*** | **PV094758** | - | - | - | - | **This study** |
| ***Hortiboletus bubalinus*** | **H6045679** | **Finland: Nylandia** | ***Populus tremula*** | **PV094759** | - | - | - | - | **This study** |
| ***Hortiboletus bubalinus*** | **GS10414** | **France: Grand Est** | ***Betula*** | **PV094756** | - | - | - | - | **This study** |
| ***Hortiboletus bubalinus*** | **K-M000168095 (AT1998006)** | **Germany: Hesse** | **mixed woodland** | **PV094763** | - | - | - | - | **This study** |
| ***Hortiboletus bubalinus*** | **GK3993** | **Greece: Central Macedonia** | ***Fagus sylvatica*** | **PV094755** | - | - | - | - | **This study** |
| ***Hortiboletus bubalinus*** | **AL 22-82** | **Hungary: Bács-Kiskun** | ***Tilia cordata*** | **-** | - | **PV088293** | **PV088303** | - | **This study** |
| ***Hortiboletus bubalinus*** | **AL 22-91** | **Hungary: Budapest** | ***Populus alba*** | **PV094860** | - | **PV088294** | **PV088304** | - | **This study** |
| ***Hortiboletus bubalinus*** | **AL 22-75** | **Hungary: Nógrád** | ***Carpinus betulus* and *Salix alba*** | **PV094859** | - | **PV088292** | - | - | **This study** |
| ***Hortiboletus bubalinus*** | **AL 12-28** | **Hungary: Pest** | ***Populus* hyb.** | **PV094751** | - | **PV088291** | - | - | **This study** |
| ***Hortiboletus bubalinus*** | **AL 14-19** | **Hungary: Pest** | ***Populus* hyb.** | **PV094752** | - | - | - | - | **This study** |
| ***Hortiboletus bubalinus*** | **DB5078** | **Hungary: Csongrád-Csanád** | ***Tilia*** | **PV094754** | - | - | - | - | **This study** |
| ***Hortiboletus bubalinus*** | **MCVE25582 (MG163)** | **Italy: Lazio** | ***Tilia platyphyllos*, *Populus nigra* var. *italica*, and *Quercus ilex*** | **PV094862** | - | - | - | - | **This study** |
| ***Hortiboletus bubalinus*** | **TUR-A 209256** | **Italy: Sicily** | ***Cedrus* sp.*, Quercus ilex, Pinus* spp.*, Pseudotsuga menziesii*, and *Juglans regia*** | **PV094786** | - | - | - | - | **This study** |
| ***Hortiboletus bubalinus*** | **TUR-A 209574** | **Italy: Sicily** | ***Cedrus* sp.*, Quercus ilex, Pinus* spp.*, Pseudotsuga menziesii*, and *Juglans regia*** | **PV094787** | **PV061848** | **PV088295** | **PV088305** | - | **This study** |
| ***Hortiboletus bubalinus*** | **TUR-A 209575** | **Italy: Sicily** | ***Cedrus* sp.*, Quercus ilex, Pinus* spp.*, Pseudotsuga menziesii*, and *Juglans regia*** | **PV094788** | **PV061849** | **PV088296** | **PV088306** | - | **This study** |
| ***Hortiboletus bubalinus*** | **L0053449** | **Netherlands: North Holland** | ***Pinus* and *Populus*** | **PV094766**  **PV094767** | - | - | - | **holotype** | **This study** |
| ***Hortiboletus bubalinus*** | **L0069896** | **Netherlands: South Holland** | ***Tilia*** | **PV094768**  **PV094769** | - | - | - | - | **This study** |
| ***Hortiboletus bubalinus*** | **O-F-67109** | **Norway: Oslo Co.** | ***Tilia*** | **PV094780** | - | - | - | - | **This study** |
| ***Hortiboletus bubalinus*** | **O-F-248341** | **Norway: Oslo Co.** | ***Picea abies* dominated forest** | **PV094782** | - | - | - | - | **This study** |
| ***Hortiboletus bubalinus*** | **O-F-254512** | **Norway: Oslo Co.** | ***Tilia* and *Corylus*** | **PV094783** | - | - | - | - | **This study** |
| ***Hortiboletus bubalinus*** | **O-F-270491** | **Norway: Oslo Co.** | ***Tilia*** | **PV094784** | - | - | - | - | **This study** |
| ***Hortiboletus bubalinus*** | **O-F-73245** | **Norway: Oslo Co.** | **Unknown** | **PV094863*** | - | - | - | - | **This study** |
| ***Hortiboletus bubalinus*** | **O-F-307797** | **Norway: Ostfold Co.** | ***Fagus* and *Betula*** | **PV094785** | - | - | - | - | **This study** |
| ***Hortiboletus bubalinus*** | **O-F-204266** | **Norway: Telemark Co.** | ***Corylus, Salix, Quercus,* and *Pinus*** | **PV094781** | - | - | - | - | **This study** |
| ***Hortiboletus bubalinus*** | **LE F-315839** | **Russia: Saint-Petersburg** | ***Tilia cordata*** | **PV094773** | PP313106 | PP320315 | - | - | **This study**, Pham et al. 2024 |
| ***Hortiboletus bubalinus*** | **LE F-332077** | **Russia: Saint-Petersburg** | ***Picea* *abies* and *Tilia cordata*** | **PV094776** | - | - | - | - | **This study** |
| ***Hortiboletus bubalinus*** | **LE F-332078** | **Russia: Saint-Petersburg** | ***Tilia cordata*** | **PV094777** | - | - | - | - | **This study** |
| ***Hortiboletus bubalinus*** | **LE F-332079** | **Russia: Saint-Petersburg** | ***Picea abies*** | **PV094778** | - | - | - | - | **This study** |
| ***Hortiboletus bubalinus*** | **LE F-332080** | **Russia: Saint-Petersburg** | ***Picea abies*** | **PV094779** | - | - | - | - | **This study** |
| ***Hortiboletus bubalinus*** | **LE F-315838** | **Russia: Saint-Petersburg** | ***Picea abies* and *Tilia cordata*** | **PV094772** | - | - | - | - | **This study** |
| ***Hortiboletus bubalinus*** | **LE F-332075** | **Russia: Saint-Petersburg** | ***Tilia cordata*** | **PV094775** | - | - | - | - | **This study** |
| ***Hortiboletus bubalinus*** | **LE F-254151** | **Russia: Saint-Petersburg** | ***Tilia cordata* and *Corylus avellana*** | **PV094771** | - | - | - | - | **This study** |
| ***Hortiboletus bubalinus*** | **LE F-254144** | **Russia: Saint-Petersburg** | ***Tilia cordata*** | **PV094770** | - | - | - | - | **This study** |
| ***Hortiboletus bubalinus*** | **LE F-315962** | **Russia: Pskov Oblast** | **mixed coniferous-deciduos forest** | **PV094774** | - | - | - | - | **This study** |
| ***Hortiboletus bubalinus*** | **GS10506** | **Russia: Udmurt Republic** | ***Populus tremula, Tilia cordata, Ulmus glabra*** | **PV094757** | - | - | - | - | **This study** |
| ***Hortiboletus bubalinus*** | **K-M001435682 (AB B20-368)** | **Serbia: Central Serbia** | ***Tilia cordata*** | **PV094765** | - | - | - | - | **This study** |
| ***Hortiboletus bubalinus*** | **JAM0593** | **Spain: Cantabria** | ***Corylus avellana* and *Populus nigra*** | **PV094760**  **PV094761**  **PV094762** | - | - | - | - | **This study** |
| ***Hortiboletus bubalinus*** | **AT2002036** | **Sweden: Södermanland Co.** | ***Betula*** | **PV094753** | - | - | - | - | **This study** |
| ***Hortiboletus bubalinus*** | **K-M000172000 (AH2006071)** | **UK: England** | ***Carpinus, Tilia, Cornus*** | **PV094764** | - | - | - | - | **This study** |
| *Hortiboletus bubalinus* (as *Xerocomellus porosporus*) | BR5020031124846 (VDKO0311) | Belgium: Meise | *Platanus* sp. | **-** | **-** | MH614727 | MH614773 | **-** | Vadthanarat et al. 2019 |
| *Hortiboletus bubalinus* (as *Hortiboletus* sp.) | KR-M-0044351 | Germany | Unknown | MT005978 | **-** | **-** | **-** | **-** | Scholler unpubl. |
| *Hortiboletus bubalinus* (as Uncultured Boletaceae) | EM 16 | Germany: Hamburg | *Populus tremula* | AJ510272 | **-** | **-** | **-** | environmental sample | Kaldorf et al. 2004 |
| *Hortiboletus bubalinus* | PDD 103177 | New Zealand | *Populus tremula* | **-** | OP141595 | **-** | **-** | **-** | Cooper & Park unpubl. |
| *Hortiboletus bubalinus* | PDD 106723 (JAC14341) | New Zealand: Manawatū-Whanganui | *Pinus radiata* | OP141407 | OP141554 | **-** | **-** | **-** | Cooper & Park unpubl. |
| *Hortiboletus bubalinus* | PDD 106682 (JAC14342) | New Zealand: Manawatū-Whanganui | *Pinus radiata* | OP141408 | **-** | **-** | **-** | **-** | Cooper & Park unpubl. |
| *Hortiboletus bubalinus* (as *Hortiboletus* sp. *'bubalinus*-PNW02') | iNaturalist 185971387 | USA: Connecticut | Unknown | PQ256335 | **-** | **-** | **-** | **-** | Ostuni et al. unpubl. |
| *Hortiboletus bubalinus* (as *Xerocomellus* cf. *rubellus*) | MB05-008 | USA: Massachusetts | Unknown | **-** | KF030296 | **-** | **-** | **-** | Nuhn et al. 2013 |
| *Hortiboletus bubalinus* (as *Xerocomus* aff. *bubalinus*) | NY04106745 (ALV16747) | USA: New York | Mixed hardwood forest | MK408628 | MK513711 | **-** | **-** | **-** | Horman  unpubl. |
| *Hortiboletus bubalinus* | iNaturalist 174321101 | USA: New York | Unknown | OR800146 | **-** | **-** | **-** | **-** | Jakob unpubl. |
| *Hortiboletus bubalinus* (as *Hortiboletus* sp. *'bubalinus*-PNW02') | iNaturalist 182899477 (clone 4805787) | USA: Oregon | Unknown | PP574057 | **-** | **-** | **-** | **-** | Ostuni et al. unpubl. |
| *Hortiboletus bubalinus* (as *Xerocomellus* cf. *rubellus*) | DW101414 | USA: Washington | Unknown | **-** | KU144804 | **-** | **-** | **-** | Frank et al. 2020 |
| ***Hortiboletus campestris*** | **MICH4999** | **USA: Michigan** | **Unknown** | **PV036230** | **PV036235** | **PV106168** | **PV106172** | **holotype** | **This study** |
| ***Hortiboletus campestris*** | **MICH10011** | **USA: Michigan** | ***Picea*** | **PV036229** | **PV036234** | **PV106169** | **PV106173** | **holotype of *Boletus harrisonii*** | **This study** |
| *Hortiboletus campestris* (as *Hortiboletus* sp. 'IN01') | iNaturalist 130550051. | USA: Indiana | Unknown | OP642912 | **-** | **-** | **-** | **-** | Russell unpublished |
| *Hortiboletus campestris* (as *Hortiboletus* sp. 'IN01') | iNaturalist 128603275 | USA: Indiana | Unknown | OP470493 | **-** | **-** | **-** | **-** | Russell unpublished |
| *Hortiboletus campestris* (as *Hortiboletus* sp. 'IN01') | iNaturalist 91671059 | USA: Indiana | Unknown | OM972507 | **-** | **-** | **-** | **-** | Russell unpublished |
| *Hortiboletus campestris* (as *Hortiboletus* sp. 'IN01') | iNaturalist 91671838 | USA: Indiana | Unknown | OM972506 | **-** | **-** | **-** | **-** | Russell unpublished |
| *Hortiboletus campestris* (as *Hortiboletus* sp. 'IN01') | Mushroom Observer 106649 | USA: Indiana | Unknown | OM747646 | **-** | **-** | **-** | **-** | Russell unpublished |
| *Hortiboletus campestris* (as *Boletoideae* sp.) | Mushroom Observer 327553 | USA: New York | Unknown | MW882065 | **-** | **-** | **-** | **-** | Safonov unpublished |
| *Hortiboletus campestris* (as *Boletoideae* sp.) | Mushroom Observer 327973 | USA: New York | Unknown | MW882076 | **-** | **-** | **-** | **-** | Safonov unpublished |
| *Hortiboletus coccyginus* | CFS111711 (JLF_X01) | USA: California | Unknown | KU144818 | **-** | **-** | **-** | **-** | Frank et al. 2020 |
| *Hortiboletus coccyginus* | NS110511 (JLF_X02) | USA: Oregon | Unknown | KU144819 | **-** | **-** | **-** | **-** | Frank et al. 2020 |
| *Hortiboletus coccyginus* (also as *Xerocomellus* cf. *rubellus*) | JLF3093 (OSC162211) | USA: Washington | Unknown | KU144805 | **-** | **-** | MW737473 | **-** | Frank et al. 2020; Farid et al. 2021 |
| *Hortiboletus coccyginus* | iNaturalist 34423971 | USA: Washington | *Pseudotsuga menziesii* | OM522247 | **-** | **-** | **-** | **-** | Ness et al. unpubl. |
| *Hortiboletus coccyginus* | iNaturalist 14291078 | USA | Unknown | MN049498 | **-** | **-** | **-** | **-** | Schwarz unpubl. |
| ***Hortiboletus engelii* (as *Xerocomus rubellus*)** | **IB19990917** | **Croatia: Primorje-Gorski Kotar Co.** | ***Quercus ilex* and *Olea europaea*** | **PV094810** | AF514828 | - | - | - | **This study,** Peintner at al. 2003 |
| ***Hortiboletus engelii*** | **K-M000170238 (AH2008092)** | **Czechia: South Moravian reg.** | ***Quercus* sp.** | **PV094817** | - | - | - | - | **This study** |
| ***Hortiboletus engelii* (as *Xerocomus communis*)** | **MCVE18563 (AT1998001, GS2230)** | **Germany: Hesse** | ***Quercus* sp.** | **UDB000446** | - | - | - | - | **This study** |
| ***Hortiboletus engelii*** | **AT1998032** | **Germany: Rhineland-Palatinate** | **Mixed woodland, mainly *Quercus*** | **PV094793** | - | - | - | - | **This study** |
| ***Hortiboletus engelii*** | **AT1998037** | **Germany: Rhineland-Palatinate** | ***Quercus* sp.** | **PV094794** | - | - | - | - | **This study** |
| ***Hortiboletus engelii*** | **AT1998122** | **Germany: Hesse** | ***Quercus* sp.** | **PV094825*** | - | - | - | - | **This study** |
| ***Hortiboletus engelii*** | **ACAM2012-105** | **Greece: Attica** | ***Quercus ilex* and *Q. coccifera*** | **PV094789** | - | - | - | - | **This study** |
| ***Hortiboletus engelii*** | **EP05-M224** | **Greece: South Aegean** | ***Quercus coccifera*** | **PV094797** | - | - | - | - | **This study** |
| ***Hortiboletus engelii*** | **GK4141** | **Greece: West Macedonia** | ***Carpinus* sp. & *Robinia pseudoacacia*** | **PV094864*** | - | - | - | - | **This study** |
| ***Hortiboletus engelii*** | **GK6124** | **Greece: West Macedonia** | ***Quercus frainetto*** | **PV094799** | - | - | - | - | **This study** |
| ***Hortiboletus engelii*** | **GK8287** | **Greece: West Macedonia** | ***Quercus frainetto*** | **PV094800** | - | - | - | - | **This study** |
| ***Hortiboletus engelii*** | **GK8378** | **Greece: West Macedonia** | ***Carpinus* sp. and *Robinia pseudoacacia*** | **PV094801** | - | - | - | - | **This study** |
| ***Hortiboletus engelii*** | **GK9736** | **Greece: West Macedonia** | ***Carpinus orientalis*, *Robinia pseudacacia* and *Corylus avelana*** | **PV094802** | - | - | - | - | **This study** |
| ***Hortiboletus engelii*** | **GK11644** | **Greece: Ionian Islands** | ***Quercus*** | **PV094803** | - | - | - | - | **This study** |
| ***Hortiboletus engelii*** | **FP-2016-06-06** | **Hungary: Fejér** | ***Corylus avellana*** | **PV094798** | - | - | - | - | **This study** |
| ***Hortiboletus engelii*** | **AL 15-56** | **Hungary: Heves** | ***Carpinus betulus*** | **PV094790** | - | - | - | - | **This study** |
| ***Hortiboletus engelii*** | **AL 21-73** | **Hungary: Pest** | ***Carpinus betulus*** | **PV094791** | - | - | **PV088307** | - | **This study** |
| ***Hortiboletus engelii*** | **DB-2022-06-12-3** | **Hungary: Komárom-**  **Esztergom** | ***Quercus cerris* and *Q. petraea*** | **PV094796** | - | **PV088297** | **PV088308** | - | **This study** |
| ***Hortiboletus engelii*** | **MCVE18267 (GS1896)** | **Italy: Emilia-Romagna** | ***Tilia platyphyllos*** | **PV094804** | - | - | - | - | **This study** |
| ***Hortiboletus engelii*** | **GS10409** | **Italy: Emilia-Romagna** | ***Carpinus betulus* and *Quercus petraea*** | **PV094807** | - | - | - | - | **This study** |
| ***Hortiboletus engelii*** | **GS10410** | **Italy: Emilia-Romagna** | ***Carpinus betulus*, *Quercus petraea* and *Taxodium distichum*** | **PV094808** | - | - | - | - | **This study** |
| ***Hortiboletus engelii*** | **GS10138** | **Italy: Emilia-Romagna** | ***Tilia platyphyllos* and *Populus alba*** | **PV094805** | - | - | - | - | **This study** |
| ***Hortiboletus engelii*** | **IB1997735** | **Italy: Sardinia** | ***Quercus suber*** | **PV030525** | | - | - | - | **This study** |
| ***Hortiboletus engelii*** | **GS10273** | **Italy: Sardinia** | ***Quercus ilex* and *Cupressus horizontalis*** | **PV094806** | - | - | - | - | **This study** |
| ***Hortiboletus engelii*** | **TUR-A 209576** | **Italy: Sicily** | ***Quercus ilex* and *Pinus pinea*** | **PV094823** | **PV061850** | **PV088298** | **PV088309** | - | **This study** |
| ***Hortiboletus engelii*** | **TUR-A 209577** | **Italy: Sicily** | ***Quercus ilex* and *Pinus pinea*** | **PV094824** | **PV061851** | **PV088299** | **PV088310** | - | **This study** |
| ***Hortiboletus engelii*** | **SAF 1994** | **Italy: Sicily** | ***Quercus ilex*** | **PV030526** | | - | - | - | **This study** |
| ***Hortiboletus engelii*** | **GS10848** | **Italy: Tuscany** | ***Quercus cerris, Acer sp., Ostrya carpinifolia, Fraxinus ornus*** | **PV094809** | - | - | - | - | **This study** |
| ***Hortiboletus engelii*** | **LE F-332232** | **Russia: Krasnodarsky Krai** | ***Fagus, Carpinus, Quercus*** | **PV094822** | - | - | - | - | **This study** |
| ***Hortiboletus engelii*** | **K-M001435694 (AB B20-362)** | **Serbia: Central Serbia** | ***Fagus sylvatica* and *Quercus petraea*** | **PV094819** | - | - | - | - | **This study** |
| ***Hortiboletus engelii*** | **JAM0547** | **Spain: Cantabria** | ***Fagus sylvatica*** | **PV094811** | - | - | - | - | **This study** |
| ***Hortiboletus engelii*** | **JAM0591** | **Spain: Cantabria** | ***Quercus robur*** | **PV094812** | - | - | - | - | **This study** |
| ***Hortiboletus engelii*** | **JAM0605** | **Spain: Bizkaia** | ***Quercus robur*** | **PV094813** | - | - | - | - | **This study** |
| ***Hortiboletus engelii*** | **JAM0611** | **Spain: Navarra** | ***Fagus sylvatica*** | **PV094814** | - | - | - | - | **This study** |
| ***Hortiboletus engelii*** | **AT2003001** | **Sweden: Stockholm Co.** | **in greenhouse, probably associated with *Tilia*** | **PV094795** | - | - | - | - | **This study** |
| ***Hortiboletus engelii*** | **LD 1213132** | **Sweden: Scania Co.** | ***Quercus*** | **PV094821** | - | - | - | - | **This study** |
| ***Hortiboletus engelii*** | **AT1994006** | **UK: England** | ***Quercus cerris*** | **PV094792** | - | - | - | - | **This study** |
| ***Hortiboletus engelii*** | **K-M000167206 (AH2004079)** | **UK: England** | ***Quercus ilex*** | **PV094815** | - | - | - | - | **This study** |
| ***Hortiboletus engelii*** | **K-M000167946 (AH1996033)** | **UK: England** | **Unknown** | **UDB000444** | - | - | - | - | **This study** |
| ***Hortiboletus engelii*** | **K-M000167271 (AH2004100)** | **UK: England** | **Unknown** | **PV094816** | - | - | - | - | **This study** |
| ***Hortiboletus engelii*** | **K-M000170839 (AH1998100)** | **UK: England** | ***Quercus robur*** | **PV094818** | - | - | - | - | **This study** |
| ***Hortiboletus engelii*** | **K-M001435697 (AB B14-246)** | **Ukraine: Vinnytsia reg.** | ***Fagus sylvatica, with presence of Pinus sylvestris* and *Carpinus betulus*** | **PV094820** | - | - | - | - | **This study** |
| *Hortiboletus engelii* (as *Xerocomus rubellus*) | IB2004272 | Austria: Carinthia | *Populus tremula* | EF644119 | **-** | **-** | **-** | **-** | Krpata et al. 2008 |
| *Hortiboletus engelii* (as Uncultured ectomycorrhiza (*Xerocomus*)) | 3Me2 | Austria: Carinthia | *Populus tremula* | EF644171 | **-** | **-** | **-** | environmental sample | Krpata et al. 2008 |
| *Hortiboletus engelii* (as *Xerocomus communis*) | JV00-362 | Denmark: Southern Denmark | *Betula* | UDB001387 | **-** | **-** | **-** | **-** | Kjöller unpublished |
| *Hortiboletus engelii* (as *Xerocomus communis*) | JV92-887 | Denmark: Southern Denmark | Unknown | UDB001386 | **-** | **-** | **-** | **-** | Kjöller unpublished |
| *Hortiboletus engelii* (as *Boletus* sp.) | LM4728 | France: Île-de-France | *Quercus petraea* and *Q. robur* | KM576320 | **-** | **-** | **-** | environmental sample | Suz et al. 2014 |
| *Hortiboletus engelii* (as Uncultured fungus) | C.Napoli_clone6 | France: Occitania | *Tuber melanosporum* truffle-ground soil, burnt area | FN391302 | **-** | **-** | **-** | environmental sample | Napoli  et al. 2010 |
| *Hortiboletus engelii* (as *Xerocomus rubellus*) | GS961 | Italy: Emilia-Romagna | *Quercus cerris*, *Tilia* | **-** | AF514829 | **-** | **-** | **-** | Peintner et al. 2003 |
| *Hortiboletus engelii* (as *Xerocomus rubellus*) | Hal-BP-20 | Italy: Sardinia | *Halimium halimifolium* | MT594509 | **-** | **-** | **-** | **-** | Leonardi et al. 2020 |
| *Hortiboletus engelii* (as Uncultured *Xerocomus*) | s0m001 | Italy: Sardinia | *Quercus suber* | HF565082 | **-** | **-** | **-** | environmental sample | Lancellotti & Franceschini unpubl. |
| *Hortiboletus engelii* | SM333 (B210c) | Malta | *Quercus ilex* | OR567838 | **-** | **-** | **-** | **-** | Mifsud & Mifsud unpubl. |
| *Hortiboletus engelii* | SM333 (B210d) | Malta | *Quercus ilex* | OR567839 | **-** | **-** | **-** | **-** | Mifsud & Mifsud unpubl. |
| *Hortiboletus engelii* (as *Xerocomus rubellus*) | XRQ-VII-09 | Montenegro | *Quercus ilex* | JQ685725 | **-** | **-** | **-** | **-** | Lazarevic et al. unpubl. |
| *Hortiboletus engelii* (as *Boletus rubellus*) | LAH0810 | Pakistan: Khyber Pakhtunkhwa | *Pinus wallichiana* | KJ802929 | **-** | **-** | **-** | **-** | Sarwar et al. 2016 |
| *Hortiboletus engelii* (as *Boletus rubellus*) | LAH0710 | Pakistan: Khyber Pakhtunkhwa | *Quercus incana* | KJ802928 | **-** | **-** | **-** | **-** | Sarwar et al. 2016 |
| *Hortiboletus engelii* (as *Boletus rubellus*) | ChL22 | Poland: Lublin Voivodeship | *Corylus avellana* | KX438318 | **-** | **-** | **-** | **-** | Szmidla et al. unpubl. |
| *Hortiboletus engelii* | LM2599 | Romania: Ilfov Co. | *Quercus robur* | PP680226 | **-** | **-** | **-** | environmental sample | Suz et al. 2014 |
| *Hortiboletus engelii* (as *Hortiboletus rubellus*) | iNaturalist 187446512 (HAY-F-005479) | USA: California | Unknown | PP578058 | **-** | **-** | **-** | **-** | D'Elia et al. unpubl. |
| ***Hortiboletus flavorubellus*** | **MICH10015** | **USA: Michigan** | **Unknown** | **PV036233** | **PV036238** | **PV106170** | **PV106174** | **holotype** | **This study** |
| ***Hortiboletus flavorubellus*** | **MICH10035** | **USA: Michigan** | ***Quercus* sp. and *Pinus* sp.** | **PV036231**  **PV036232** | **PV036236**  **PV036237** | **PV106171** | **PV106175** | **holotype of *Boletus rubellus* var. *flammeus*** | **This study** |
| *Hortiboletus flavorubellus* (as *Hortiboletus* sp.) | TUE003274 | Canada: Ontario | Unknown | UDB03411742 | **-** | **-** | **-** | environmental sample | Tedersoo et al. unpubl. |
| *Hortiboletus flavorubellus* (as *Hortiboletus campestris*) | iNaturalist 66900805 | Canada: Quebec | Unknown | ON943310 | *-* | *-* | *-* | *-* | Lebeuf et al. unpubl. |
| *Hortiboletus flavorubellus* (as *Boletales* sp.) | B0072 | Canada | Unknown | KY825962 | **-** | **-** | **-** | **-** | Stefani & Archambault  unpubl. |
| *Hortiboletus flavorubellus* (as *Hortiboletus* sp. '*campestris*') | DD614 | USA: California | Unknown | MH168538 | MH203598 | **-** | **-** | **-** | Frank et al. 2020 |
| *Hortiboletus flavorubellus* (as *Hortiboletus* sp. 'CA01') | iNaturalist 184339160 (HAY-F-004236) | USA: California | Unknown | PP971225 | **-** | **-** | **-** | **-** | D'Elia et al. unpubl. |
| *Hortiboletus flavorubellus* (as *Hortiboletus* sp. 'IN04') | iNaturalist 180076059 | USA: Connecticut | Unknown | PP574113 | **-** | **-** | **-** | **-** | Ostuni et al. unpubl. |
| *Hortiboletus flavorubellus* (as *Hortiboletus rubellus*) | iNaturalist 134899881 | USA: Florida | Unknown | OQ363127 | **-** | **-** | **-** | **-** | Smith & Lemmond unpubl. |
| *Hortiboletus flavorubellus* (as *Hortiboletus rubellus*) | FLAS-F-60513 | USA: Florida | Unknown | MH211664 | **-** | **-** | **-** | **-** | Kaminsky et al. unpubl. |
| *Hortiboletus flavorubellus* (as *Hortiboletus* sp. 'IN02') | iNaturalist 165432073 | USA: Florida | Unknown | OR825553 | **-** | **-** | **-** | **-** | Ostuni et al. unpubl. |
| *Hortiboletus flavorubellus* (as *Hortiboletus* sp. 'IN02') | iNaturalist 69895981 | USA: Florida | Unknown | PP791150 | **-** | **-** | **-** | **-** | Ostuni et al. unpubl. |
| *Hortiboletus flavorubellus* (as *Hortiboletus rubellus*) | FLAS-F-61506 | USA: Florida | *Carya* and *Quercus* forest | MH211937 | **-** | **-** | **-** | **-** | Kaminsky et al. unpubl. |
| *Hortiboletus flavorubellus* (as *Hortiboletus* sp. 'IN02'*)* | iNaturalist 186222765 | USA: Florida | Unknown | PP850649 | **-** | **-** | **-** | **-** | Ostuni et al. unpubl. |
| *Hortiboletus flavorubellus* (as *Hortiboletus rubellus*) | FLAS-F-60503 | USA: Georgia | *Carya illinoinensis* | MH211661 | **-** | **-** | **-** | **-** | Kaminsky et al. unpubl. |
| *Hortiboletus flavorubellus* (as *Boletus subfraternus*) | MICH KUO-08101302 | USA: Illinois | *Tilia americana*, *Fraxinus* and *Acer pseudoplatanus* | **-** | MK601724 | MK721078 | MK766286 | **-** | Kuo & Ortiz-Santana 2020 |
| *Hortiboletus flavorubellus* (as *Boletus subfraternus*) | MICH KUO-06081002 | USA: Illinois | *Quercus alba*, *Acer* sp. | **-** | MK601741 | MK721095 | MK766303 | **-** | Kuo & Ortiz-Santana 2020 |
| *Hortiboletus flavorubellus* (as *Boletus harrisonii*) | MICH KUO-09071204 | USA: Illinois | *Acer* spp.*, Fraxinus, Quercus* | **-** | MK601718 | MK721072 | MK766280 | **-** | Kuo & Ortiz-Santana 2020 |
| *Hortiboletus flavorubellus* (as *Hortiboletus campestris*) | MICH KUO-08240502 | USA: Illinois | *Pinus strobus* | **-** | **-** | MK721094 | MK766302 | **-** | Kuo & Ortiz-Santana 2020 |
| *Hortiboletus flavorubellus* (as *Boletus campestris*) | F:PRL5991MAN | USA: Indiana | *Quercus* | GQ166879 | **-** | **-** | **-** | **-** | Avis et al. unpubl. |
| *Hortiboletus flavorubellus* (as *Boletus campestris*) | F:PRL5879MAN | USA: Indiana | *Quercus* | GQ166876 | **-** | **-** | **-** | **-** | Avis et al. unpubl. |
| *Hortiboletus flavorubellus* (as *Boletus rubellus*) | F:PRL5788MAN | USA: Indiana | *Quercus* dominated woodland on moraine soil | GQ166883 | **-** | **-** | **-** | **-** | Avis et al. unpubl. |
| *Hortiboletus flavorubellus* (as *Hortiboletus* sp. 'IN03') | iNaturalist 92226980 | USA: Indiana | Unknown | OP455714 | **-** | **-** | **-** | **-** | Russell  unpubl. |
| *Hortiboletus flavorubellus* (as *Hortiboletus* sp. 'IN03') | iNaturalist 91658700 | USA: Indiana | Unknown | OM972514 | **-** | **-** | **-** | **-** | Russell  unpubl. |
| *Hortiboletus flavorubellus* (as *Hortiboletus* sp. 'IN04') | iNaturalist 130825283 | USA: Indiana | Unknown | OP643031 | **-** | **-** | **-** | **-** | Russell  unpubl. |
| *Hortiboletus* *flavorubellus* (as *Xerocomellus* cf. *rubellus*) | MB03-033 | USA: Massuchusetts | Unknown | **-** | KF030294 | KF030419 | **-** | **-** | Nuhn et al. 2013 |
| *Hortiboletus flavorubellus* (as *Hortiboletus* sp. 'IN04') | iNaturalist 173995916 | USA: Massuchusetts | *Pinus*, *Quercus* | PP156414 | **-** | **-** | **-** | **-** | Ostuni et al. unpubl. |
| *Hortiboletus flavorubellus* (as *Hortiboletus* sp.) | Mushroom Observer 373444 | USA: New Jersey | *Pinus*, *Quercus* | ON705313  ON705314 | **-** | **-** | **-** | **-** | Safonov and Kudzma unpubl. |
| *Hortiboletus flavorubellus* (as *Boletus* aff*. rubellus*) | goldie_U2LsC | USA: New York | *Castanea dentata* | JX030209 | **-** | **-** | **-** | environmental sample | Tourtellot unpublished |
| *Hortiboletus flavorubellus* (as *Boletus* aff*. rubellus*) | U1Z_3 | USA: New York | *Castanea dentata* | JX030290 | **-** | **-** | **-** | environmental sample | Tourtellot unpublished |
| *Hortiboletus flavorubellus* (as *Boletus subfraternus*) | Mushroom Observer 299731 | USA: Ohio | *Betula* sp. *Quercus* sect. *Lobatae* | MK607543 | **-** | **-** | **-** | **-** | Russell and Grootmyers unpubl. |
| *Hortiboletus flavorubellus* (as *Hortiboletus* sp. 'IN02') | iNaturalist 173105912 | USA: Ohio | Unknown | OR882636 | **-** | **-** | **-** | **-** | Ostuni et al. unpubl. |
| *Hortiboletus flavorubellus* (as *Hortiboletus* sp. 'IN02') | iNaturalist 180935693 | USA: Ohio | Unknown | PP156339 | **-** | **-** | **-** | **-** | Ostuni et al. unpubl. |
| *Hortiboletus flavorubellus* (as *Hortiboletus* sp. 'IN03') | iNaturalist 180516778 | USA: Ohio | Unknown | PP436628 | **-** | **-** | **-** | **-** | Ostuni et al. unpubl. |
| *Hortiboletus flavorubellus* (as *Hortiboletus* sp. 'IN03') | iNaturalist 176803372 | USA: Ohio | *Quercus* | OR987157 | **-** | **-** | **-** | **-** | Ostuni et al. unpubl. |
| *Hortiboletus flavorubellus* (as *Hortiboletus* sp. 'IN03') | iNaturalist 171101119 | USA: Ohio | Unknown | OR824598 | **-** | **-** | **-** | **-** | Ostuni et al. unpubl. |
| *Hortiboletus flavorubellus* (as *Hortiboletus* sp. 'IN03') | iNaturalist 174585624 | USA: Ohio | Unknown | OR825652 | **-** | **-** | **-** | **-** | Ostuni et al. unpubl. |
| *Hortiboletus flavorubellus* (as *Hortiboletus* sp.) | iNaturalist 173087417  (JLF11622) | USA: Oregon | Unknown | OR342245 | **-** | **-** | **-** | **-** | Frank unpubl. |
| *Hortiboletus flavorubellus* (as *Hortiboletus* sp.) | MO458368 | USA: Pennsylvania | Unknown | ON134040 | **-** | **-** | **-** | **-** | Wasilewski et al. unpubl. |
| *Hortiboletus flavorubellus* (as *Hortiboletus* sp.) | MO460889 | USA: Pennsylvania | Unknown | ON134043 | **-** | **-** | **-** | **-** | Wasilewski et al. unpubl. |
| *Hortiboletus flavorubellus* (as *Hortiboletus* sp. 'IN04') | Mushroom Observer 496814 | USA: Pennsylvania | Unknown | OP749307 | **-** | **-** | **-** | **-** | Russell  unpubl. |
| *Hortiboletus flavorubellus* (as *Hortiboletus* sp. 'IN04') | Mushroom Observer 497200 | USA: Pennsylvania | Unknown | OP749312 | **-** | **-** | **-** | **-** | Russell  unpubl. |
| *Hortiboletus flavorubellus* (as *Hortiboletus* sp. 'IN04') | Mushroom Observer 498828 | USA: Pennsylvania | Unknown | OP749009 | **-** | **-** | **-** | **-** | Russell  unpubl. |
| *Hortiboletus flavorubellus* (as *Boletus harrisonii*) | PBM4097 | USA: Tennessee | *Quercus* | PQ586122 | MT237457 | **-** | **-** | **-** | Matheny unpubl.,  et al. unpubl. |
| *Hortiboletus flavorubellus* (as *Hortiboletus* sp.) | Mushroom Observer 418487 | USA: Wisconsin | Unknown | ON950120 | **-** | **-** | **-** | **-** | Safonov and Kudzma unpubl. |
| ***Hortiboletus hershenzoniae*** | **K-M001435678 (AB B12-024)** | **Israel: Carmel Mount** | ***Quercus calliprinos*** | **PV030527** | | - | - | **paratype** | **This study** |
| ***Hortiboletus hershenzoniae*** | **K-M001435594 (AB B12-071)** | **Israel: Sharon Plain** | ***Quercus calliprinos*** | **PV030528** | | **PV088300** | **PV088311** | **holotype** | **This study** |
| ***Hortiboletus hershenzoniae*** | **K-M001435698 (AB B14-217)** | **Israel: Carmel Mount** | ***Quercus calliprinos*** | **PV030529** | | **PV088301** | **PV088312** | **paratype** | **This study** |
| ***Hortiboletus hershenzoniae*** | **K-M001435704 (AB B14-218)** | **Israel: Sharon Plain** | ***Quercus calliprinos*** | **PV030530** | | - | - | **paratype** | **This study** |
| ***Hortiboletus hershenzoniae*** | **K-M001435706 (AB B14-227)** | **Israel: Sharon Plain** | ***Quercus calliprinos*** | **PV030532** | | - | - | **paratype** | **This study** |
| ***Hortiboletus hershenzoniae*** | **K-M001435708 (AB B14-228)** | **Israel: Sharon Plain** | ***Quercus calliprinos*** | **PV030531** | | - | - | **paratype** | **This study** |
| ***Hortiboletus hershenzoniae*** | **K-M001435699**  **(AB B15-260)** | **Israel: Upper Galilee** | **under *Quercus calliprinos,* near *Pistacia lentiscus* and *Laurus nobilis*** | **PV094749** | - | - | - | **paratype** | **This study** |
| ***Hortiboletus hershenzoniae*** | **K-M001436710 (AB B15-261)** | **Israel: Carmel Mount** | ***Quercus calliprinos*** | **PV094750** | - | - | - | **paratype** | **This study** |
| *Hortiboletus hershenzoniae* (as *Hortiboletus engelii*) | T1-1 | Lebanon: Akkar Governorate | *Quercus calliprinos* | MZ088076 | **-** | **-** | **-** | **-** | Sleiman et al. 2021 |
| *Hortiboletus indorubellus* | DC 14-002 | India: Sikkim | *Betula alnoides* | KT319647 | KU566807 | **-** | **-** | holotype | Das et al. 2016 |
| *Hortiboletus indorubellus* | LS15 | Pakistan | *Quercus incana* | MK002767 | MK002872 | **-** | **-** | **-** | Ullah et al. unpubl. |
| *Hortiboletus indorubellus* (as *Hortiboletus* sp.) | SS-13 | Pakistan | *Quercus floribunda* | - | OR687284 | **-** | **-** | **-** | Ullah et al. unpubl. |
| *Hortiboletus indorubellus* (as *Hortiboletus* sp.) | SS-23 | Pakistan | Unknown | OR612992 | **-** | **-** | **-** | **-** | Ullah et al. unpubl. |
| *Hortiboletus indorubellus* (as *Hortiboletus* sp.) | GS08 | Pakistan: Khyber Pakhtunkhwa | *Quercus* sp. | PQ166614 | **-** | **-** | **-** | **-** | Khan & Khan unpubl. |
| *Hortiboletus indorubellus* (as *Hortiboletus* sp.) | GS2407 | Pakistan: Khyber Pakhtunkhwa | *Quercus* sp. | PQ166615 | **-** | **-** | **-** | **-** | Khan & Khan unpubl. |
| *Hortiboletus kohistanensis* | LAH35327 (AST48) | Pakistan: Khyber Pakhtunkhwa | *Quercus incana* | MG988192 | MG988187 | **-** | **-** | holotype | Naseer et al. 2019 |
| *Hortiboletus kohistanensis* | LAH35285 (AST22) | Pakistan: Khyber Pakhtunkhwa | *Quercus incana* | MG988193 | MG988188 | **-** | **-** | paratype | Naseer et al. 2019 |
| *Hortiboletus kohistanensis* (as *Xerocomellus* aff. *rubellus*) | HKAS 51239 | China: Tibet | Unknown | **-** | KF112425 | KF112184 | KF112695 | - | Wu et al. 2014 |
| *Hortiboletus napaeus* | FHMU3326 (N.K. Zeng2272) | China: Hainan | Unknown | MT646440 | MT646433 | MT646450 | **-** | holotype | Xie et al. 2020 |
| *Hortiboletus napaeus* | FHMU3325 (N.K. Zeng1863) | China, Hainan | Unknown | MT646445 | MT646438 | MT646449 | **-** | paratype | Xie et al. 2020 |
| *Hortiboletus napaeus* | FHMU3327 (N.K. Zeng2520) | China: Hainan | Unknown | MT646441 | MT646434 | **-** | **-** | paratype | Xie et al. 2020 |
| *Hortiboletus napaeus* | FHMU3328 (N.K. Zeng2736) | China, Hainan | Unknown | MT646442 | MT646435 | MT646451 | **-** | paratype | Xie et al. 2020 |
| *Hortiboletus napaeus* | FHMU3329 (N.K. Zeng2887) | China: Hainan | Unknown | MT646443 | MT646436 | MT646452 | **-** | paratype | Xie et al. 2020 |
| ***Hortiboletus rubellus*** | **LE F-265201** | **Abkhazia: Pitsunda district** | ***Quercus-Fagus* forest** | **PV094850** | - | - | - | - | **This study** |
| ***Hortiboletus rubellus*** | **AT1998046** | **Germany: Rhineland-Palatinate** | **Mixed woodland, dominated by *Quercus* sp.** | **PV094827**  **PV094828**  **PV094829** | - | - | - | - | **This study** |
| ***Hortiboletus rubellus*** | **AT1998115** | **Germany: Hesse** | ***Quercus* sp.** | **PV094830**  **PV094831** | - | - | - | - | **This study** |
| ***Hortiboletus rubellus*** | **GK6074** | **Greece: Central Greece** | ***Fagus sylvatica*** | **PV094837** | - | - | - | - | **This study** |
| ***Hortiboletus rubellus*** | **GK6705** | **Greece: West Macedonia** | ***Quercus* sp.** | **PV094838** | - | - | - | - | **This study** |
| ***Hortiboletus rubellus*** | **K-M001435676 (AB B15-358)** | **Hungary: Csongrád-Csanád** | **Deciduous trees** | **PV094849** | - | - | - | - | **This study** |
| ***Hortiboletus rubellus*** | **AL 13-125** | **Hungary: Somogy** | ***Quercus robur*, *Carpinus betulus*, *Tilia*** | **PV094826** | - | - | - | - | **This study** |
| ***Hortiboletus rubellus*** | **DB-2022-06-29-3** | **Hungary: Vas** | ***Quercus petraea*, *Fagus sylvatica*, *Carpinus betulus*, *Pinus sylvestris*** | **PV094836** | **PV061852** | - | **PV088313** | - | **This study** |
| ***Hortiboletus rubellus*** | **VGy-2018-07-21-1** | **Hungary: Komárom-**  **Esztergom** | ***Quercus* sp., *Fagus sylvatica*, *Carpinus betulus*** | **PV094855** | - | - | - | - | **This study** |
| ***Hortiboletus rubellus*** | **MG781** | **Italy: Lazio** | ***Quercus cerris* with the presence of *Q. frainetto*, and *Crataegus* sp.** | **PV094852** | - | **PV088302** | - | - | **This study** |
| ***Hortiboletus rubellus*** | **MCVE31743 (GS10212)** | **Italy: Emilia-Romagna** | ***Quercus cerris* and *Q. pubescens*** | **PV094851** | - | - | - | **epitype** | **This study** |
| ***Hortiboletus rubellus*** | **GS1424** | **Italy: Emilia-Romagna** | ***Quercus cerris* and *Q. pubescens*** | **PV094839**  **PV094840** | - | - | - | - | **This study** |
| ***Hortiboletus rubellus*** | **MCVE18231 (GS1894)** | **Italy: Emilia-Romagna** | ***Quercus cerris*** | **PV094841** | - | - | - | - | **This study** |
| ***Hortiboletus rubellus*** | **O-F-76041** | **Norway: Ostfold Co.** | **Unknown** | **PV094853** | - | - | - | - | **This study** |
| ***Hortiboletus rubellus*** | **O-F-168828** | **Norway: Telemark Co.** | **Unknown** | **PV094854** | - | - | - | - | **This study** |
| ***Hortiboletus rubellus*** | **GS10512** | **Russia: Udmurt Republic** | ***Quercus robur*, *Salix* sp.** | **PV094842** | - | - | - | - | **This study** |
| ***Hortiboletus rubellus*** | **JAM0556** | **Spain: Bizkaia** | ***Quercus robur*** | **PV094844** | - | - | - | - | **This study** |
| ***Hortiboletus rubellus*** | **JAM0224** | **Spain: Burgos** | ***Quercus faginea*** | **PV094843** | - | - | - | - | **This study** |
| ***Hortiboletus rubellus*** | **IB19991021** | **Spain: Segovia** | ***Pinus sylvestris*, *Quercus pyrenaica*** | - | **PV061853** | - | - | - | **This study** |
| ***Hortiboletus rubellus*** | **AT2001124** | **Sweden: Stockholm Co.** | **Unknown** | **PV094832** | - | - | - | - | **This study** |
| ***Hortiboletus rubellus*** | **AT2003134** | **Sweden: Blekinge Co.** | ***Fagus* *sylvatica* and *Quercus* sp. forest** | **PV094833** | - | - | - | - | **This study** |
| ***Hortiboletus rubellus*** | **AT2004285** | **Sweden: Västra Götaland Co.** | **Mixed forest** | **PV094834** | - | - | - | - | **This study** |
| ***Hortiboletus rubellus*** | **AT2005022** | **Sweden: Uppsala Co.** | ***Quercus* sp.** | **PV094835** | - | - | - | - | **This study** |
| ***Hortiboletus rubellus*** | **K-M000168971 (AH2004055)** | **UK: England** | ***Quercus* sp.** | **PV094847**  **PV094848** | - | - | - | - | **This study** |
| ***Hortiboletus rubellus*** | **K-M000167799 (AH2005033)** | **UK: England** | ***Tilia* x *europaea*** | **PV094845 PV094846** | - | - | - | - | **This study** |
| ***Hortiboletus rubellus*** | **K-M001435677 (KW 60663F)** | **Ukraine: Vinnytsya reg.** | ***Fagus sylvatica*, *Quercus robur*** | **PV030533** | | - | - | - | **This study** |
| *Hortiboletus rubellus* | BR5020048420177 (VDKO0403) | Belgium: Meise | *Castanea sativa* | **-** | **-** | **-** | MH614774 | **-** | Vadthanarat et al. 2019 |
| *Hortiboletus rubellus* | JV00-357 | Denmark: Southern Denmark | *Betula* | UDB001406 | **-** | **-** | **-** | **-** | Kjöller unpublished |
| *Hortiboletus rubellus* | TL-4500 | Denmark: Zealand | *Fagus* | UDB001405 | **-** | **-** | **-** | **-** | Kjöller unpublished |
| *Hortiboletus rubellus* (as *Hortiboletus* sp.) | KR-M-0044796 | Germany | Unknown | MT006028 | **-** | **-** | **-** | **-** | Scholler unpubl. |
| *Hortiboletus rubellus* (as *Hortiboletus* sp.) | KR-M-0044799 | Germany | Unknown | MT006029 | **-** | **-** | **-** | **-** | Scholler unpubl. |
| *Hortiboletus rubroreticulatus* | LE F-344053 | Vietnam: Lam Dong | *Fagaceae* (*Lithocarpus* sp., *Quercus* sp.), *Theaceae (Schima wallichii), Magnoliaceae* (*Michelia* sp.) | PP317926 | PP313108 | PP320317 | **-** | holotype | Pham et al. 2024 |
| *Hortiboletus rubroreticulatus* | LE F-344054 | Vietnam: Gia Lai | Mixed forest: Podocarpaceae (*Dacrydium elatum, Dacrycarpus imbricatus*), Magnoliaceae, Burseraceae (Canarium), *Myrtaceae (Syzygium)* | PP317927 | PP313107 | PP320316 | **-** | paratype | Pham et al. 2024 |
| *Hortiboletus rufosquamosus* | BJTC FM2687 | China: Shanxi | *Quercus* | OR655155 | OR655191 | OR659992 | OR659943 | holotype | Mao et al. 2023 |
| *Hortiboletus rufosquamosus* | BJTC FM2649 | China: Shanxi | *Quercus* | OR655151 | OR655187 | OR659988 | OR659939 | paratype | Mao et al. 2023 |
| *Hortiboletus rufosquamosus* | BJTC FM2652 | China: Shanxi | *Quercus* | OR655152 | OR655188 | OR659989 | OR659940 | paratype | Mao et al. 2023 |
| *Hortiboletus rufosquamosus* | BJTC FM2660 | China: Shanxi | *Quercus* | OR655153 | OR655189 | OR659990 | OR659941 | paratype | Mao et al. 2023 |
| *Hortiboletus rufosquamosus* | BJTC FM2680 | China: Shanxi | *Quercus* | OR655154 | OR655190 | OR659991 | OR659942 | paratype | Mao et al. 2023 |
| *Hortiboletus rufosquamosus* | BJTC FM2692 | China: Shanxi | *Quercus* | OR655156 | OR655192 | OR659993 | OR659944 | paratype | Mao et al. 2023 |
| *Hortiboletus rupicapreus* | LE F-312677 | Vietnam: Gia Lai | *Myrtaceae*, *Meliaceae*, *Anacardiaceae*, *Fagaceae*, *Theaceae* | MW760391 | MW784161 | MZ424894 | **-** | holotype | Lebeuf et al. 2021 |
| *Hortiboletus rupicapreus* | LE F-312678 | Vietnam: Gia Lai | *Pinus* *kesiya* with some *Fagaceae* | MW760392 | MW784162 | MZ424893 | **-** | paratype | Lebeuf et al. 2021 |
| *Hortiboletus rupicapreus* | LE F-344055 (130VN22) | Vietnam | Unknown | PP317925 | **-** | **-** | **-** | **-** | Pham et al. 2024 |
| *Hortiboletus sinorubellus* | HMJAU68177 (W3044) | China: Henan | Unknown | **-** | OR704327 | OR663926 | OR573948 | holotype | Wang et al. 2024 |
| *Hortiboletus sinorubellus* | HMJAU68178 (W3075) | China: Henan | Unknown | **-** | OR704328 | OR663927 | **-** | paratype | Wang et al. 2024 |
| *Hortiboletus sinorubellus* | HMJAU68179 (W3076) | China: Henan | Unknown | **-** | OR704329 | OR663928 | OR573949 | paratype | Wang et al. 2024 |
| *Hortiboletus sinorubellus* | HMJAU68180 (W3100) | China: Henan | Unknown | **-** | OR704331 | OR663930 | OR573950 | paratype | Wang et al. 2024 |
| *Hortiboletus sinorubellus* | HMJAU68181 (W3369) | China: Jiangsu | Unknown | **-** | **-** | OR663938 | OR573956 | paratype | Wang et al. 2024 |
| *Hortiboletus subpaludosus* | HKAS 52659 | China: Yunnan | Unknown | **-** | KT990582 | KT990778 | KT990417 | epitype | Wu et al. 2016 |
| *Hortiboletus subpaludosus* | HKAS 68158 | China: Yunnan | Unknown | **-** | KT990583 | KT990779 | KT990418 | **-** | Wu et al. 2016 |
| *Hortiboletus subpaludosus* (as *Xerocomellus* sp.) | HKAS 59608 | China: Yunnan | Unknown | **-** | KF112371 | KF112185 | KF112551 | **-** | Wu et al. 2014 |
| *Hortiboletus tomentosus* | BJTC FM2289-A | China: Shanxi | *Quercus* | **-** | OR655193 | OR659994 | OR659945 | holotype | Mao et al. 2023 |
| *Hortiboletus tomentosus* | BJTC FM2289-B | China: Shanxi | *Quercus* | **-** | OR655227 | OR660026 | OR659977 | paratype | Mao et al. 2023 |
| *Hortiboletus tomentosus* | HMJAU68173 (W3110) | China: Henan | Unknown | **-** | OR704335 | OR663935 | **-** | **-** | Wang et al. 2024 |
| *Hortiboletus tomentosus* | HMJAU68174 (QB10274) | China: Shaanxi | Unknown | **-** | OR704336 | OR663923 | OR573957 | **-** | Wang et al. 2024 |
| *Hortiboletus tomentosus* | HMJAU68176 (QB10325) | China: Shaanxi | Unknown | **-** | OR704337 | OR663925 | **-** | **-** | Wang et al. 2024 |
| *Hortiboletus tomentosus* (as *Xerocomus* sp.) | TNS KH-JPN17-483 | Japan | Unknown | **-** | **-** | **-** | MG650106 | **-** | Hosaka and Takahashi unpubl. |
| *Hortiboletus* sp. 1 (as “*Hortiboletus swaticus*” ZU-2023a) | SWAT003365 | Pakistan: Khyber Pakhtunkhwa | Unknown | PQ870311 | PQ870339 | **-** | **-** | **-** | Khan & Khan unpubl |
| *Hortiboletus* sp. 1 (as “*Hortiboletus swaticus*” ZU-2023a) | MJ47 | Pakistan: Khyber Pakhtunkhwa | Unknown | **-** | PQ870340 | **-** | **-** | **-** | Khan & Khan unpubl |
| *Hortiboletus* sp. 1 (as “*Hortiboletus swaticus*” ZU-2023a) | MM26 | Pakistan: Khyber Pakhtunkhwa | Unknown | **-** | PQ870341 | **-** | **-** | **-** | Khan & Khan unpubl. |
| *Hortiboletus* sp. 2 (as *Hortiboletus napaeus*) | FHMU2113 (N.K. Zeng3152) | China: Hainan | Unknown | MT646444 | MT646437 | MT646446 | **-** | **-** | Xie et al. 2020 |
| *Hortiboletus* sp. 3 (as *Xerocomus* sp.) | HKAS 50466 | China | Unknown | **-** | KF112372 | KF112183 | KF112694 | **-** | Wu et al. 2014 |
| *Hortiboletus* sp. 4 (*Boletus* sp.) | YM1084 | Japan: Yamanashi | *Picea jezoensis* var. *hondoensis* | AB848414 | **-** | **-** | **-** | environmental sample | Miyamoto et al. 2014 |
| *Hortiboletus* sp. 4 (*Boletus* sp.) | KA17-0560 | South Korea: Gyeongsang | Unknown | MN294804 | **-** | **-** | **-** | **-** | Cho et al. 2020 |
| *Hortiboletus* sp. 5 (as *Boletus* cf. *rubellus)* | B1 | China? | Unknown | JX434687 | **-** | **-** | **-** | **-** | Chai unpubl. |
| *Hortiboletus* sp. 5 *(Boletus* cf. r*ubellus)* | Nara_BoR_red1 | Japan: Shizuoka | *Salix reinii* | AB211279 | **-** | **-** | **-** | **-** | Nara unpubl. |
| *Hortiboletus* sp. 6 | JLF6654 | USA: New Mexico | Unknown | MN306134 | **-** | **-** | **-** | **-** | Frank unpubl. |
| *Hortiboletus* sp. 6 | JLF6662 | USA: New Mexico | Unknown | MN306135 | **-** | **-** | **-** | **-** | Frank unpubl. |
| *Hortiboletus* sp. 6 (as *Hortiboletus campestris*) | iNaturalist 55519315 | USA: New York | Unknown | OK346512 | **-** | **-** | **-** | **-** | Desanto  et al. unpubl. |
| *Hortiboletus* sp. 6 (as *Boletus rubellus*) | JMP0013 | USA: Wisconsin | *Castanea dentata* | EU819460 | **-** | **-** | **-** | **-** | Palmer et al. 2008 |
| *Hortiboletus* sp. 6 (as Uncultured Basidiomycota) | man22_soil_E04 | USA: Michigan | *Acer saccharum*, *Tilia americana*, *Populus grandidentata*, *Fagus grandifolia*, *F. americana*, *Ostrya virginiana* | GU328505 | **-** | **-** | **-** | environmental sample | Edwards & Zak 2010 |
| *Hortiboletus* sp. 7 (*Xerocomus* sp.) | HKAS 51292 | China | Unknown | **-** | KF112369 | KF112181 | KF112692 | **-** | Wu et al. 2014 |
| *Imleria badia* | HKAS 53502 | Germany | Unknown | KC215204 | KC215213 | KC215247 | KC215235 | **-** | Zhu et al. 2014 |
| *Xerocomellus amylosporus* | JLF3498 | USA: Washington | Unknown | KU144743 | **-** | MW737492 | MW737474 | **-** | Frank et al. 2020; Farid et al. 2021 |
| *Xerocomellus atropurpureus* | OSC162192 (JLF3620) | USA: Oregon | *Pseudotsuga menziesii*, *Tsuga*  *heterophylla* | KU144749 | KU144750 | MW737495 | MW737477 | holotype | Frank et al. 2020; Farid et al. 2021 |
| *Xerocomellus atropurpureus* | NY1193858 | USA: California | Unknown | **-** | KF030271 | KF030416 | **-** | **-** | Nuhn et al. 2013, Frank et al. 2020 |
| *Xerocomellus bolinii* | JAB 95 | USA: Florida | Unknown | MW675735 | MW662589 | MW737491 | MW737472 | paratype | Farid et al. 2021 |
| *Xerocomellus chrysenteron* | La Chaneaz | Switzerland: Fribourg | *Picea abies, Fagus sylvatica* | AF402139 | | **-** | **-** | **-** | Fiore-Donno & Martin 2001 |
| *Xerocomellus chrysenteron* | HKAS 56495 | Germany | Unknown | KC215210 | KC215211 | KC215251 | **-** | **-** | Zhu et al. 2014 |
| *Xerocomellus cisalpinus* | ADK4864 | Belgium | Unknown | **-** | **-** | KT824026 | KT823993 | **-** | Raspe et al. 2016 |
| *Xerocomellus cisalpinus* | LE F-343575 | Russia: Pskov Oblast | Unknown | PP317929 | **-** | PP320318 | **-** | **-** | Pham et al. 2024 |
| *Xerocomellus cisalpinus* | PDD94421 | New Zealand | Unknown | JQ924296 | JQ924322 | KF112171 | KF112686 | **-** | Wu et al. 2014 |
| *Xerocomellus communis* | HKAS 50467 | China | Unknown | **-** | NG_059632 | KT990858 | KT990494 | holotype | Wu et al. 2014 |
| *Xerocomellus corneri* | HKAS 52503 | China | Unknown | **-** | KT990668 | KT990856 | KT990492 | - | Wu et al. 2014 |
| *Xerocomellus porosporus* | AH38964 | Spain | Unknown | KU355481 | KU355493 | **-** | **-** | **-** | Moreno et al. 2016 |
| *Xerocomellus pruinatus* | G.M. 2015-09-23.4 | Luxembourg | *Picea abies* and *Betula* | MW603181 | | **-** | **-** | **-** | Marson et al. unpubl. |
| *Xerocomellus zelleri* | OSC162212 (JLF2977) | USA: Oregon | Unknown | KM213666 | KU144799 | **-** | **-** | **-** | Frank et al. 2020 |
| *Xerocomellus* sp. | HKAS 56311 | China | Unknown | **-** | KF112340 | KF112170 | KF112684 | **-** | Wu et al. 2014 |
