# Supplementary figures and images for: ﻿Morphological and molecular re-assessment of European and Levantine species of the genus Hortiboletus (Boletaceae)
Source: IMA Fungus. 2025 Jun 12;16:e144731. doi: 10.3897/imafungus.16.144731 (PMC12179652; doi:10.3897/imafungus.16.144731)

# ITS ML

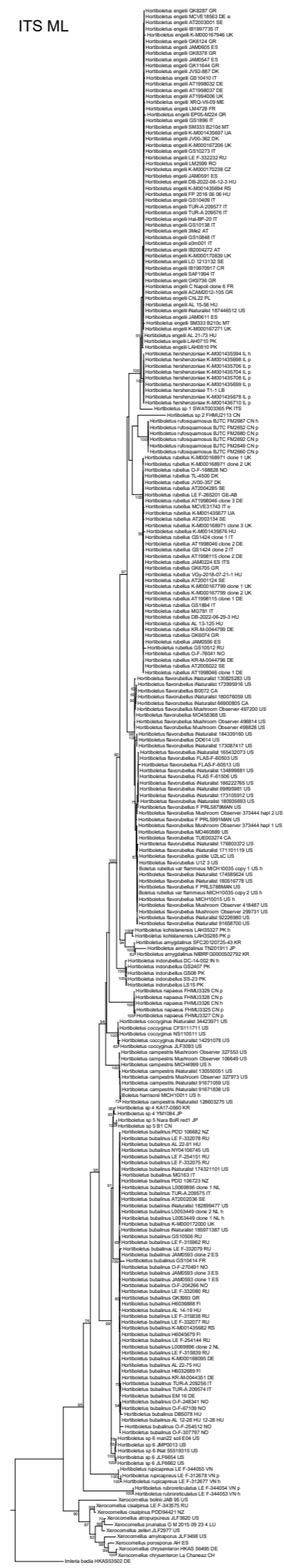

ITS BI

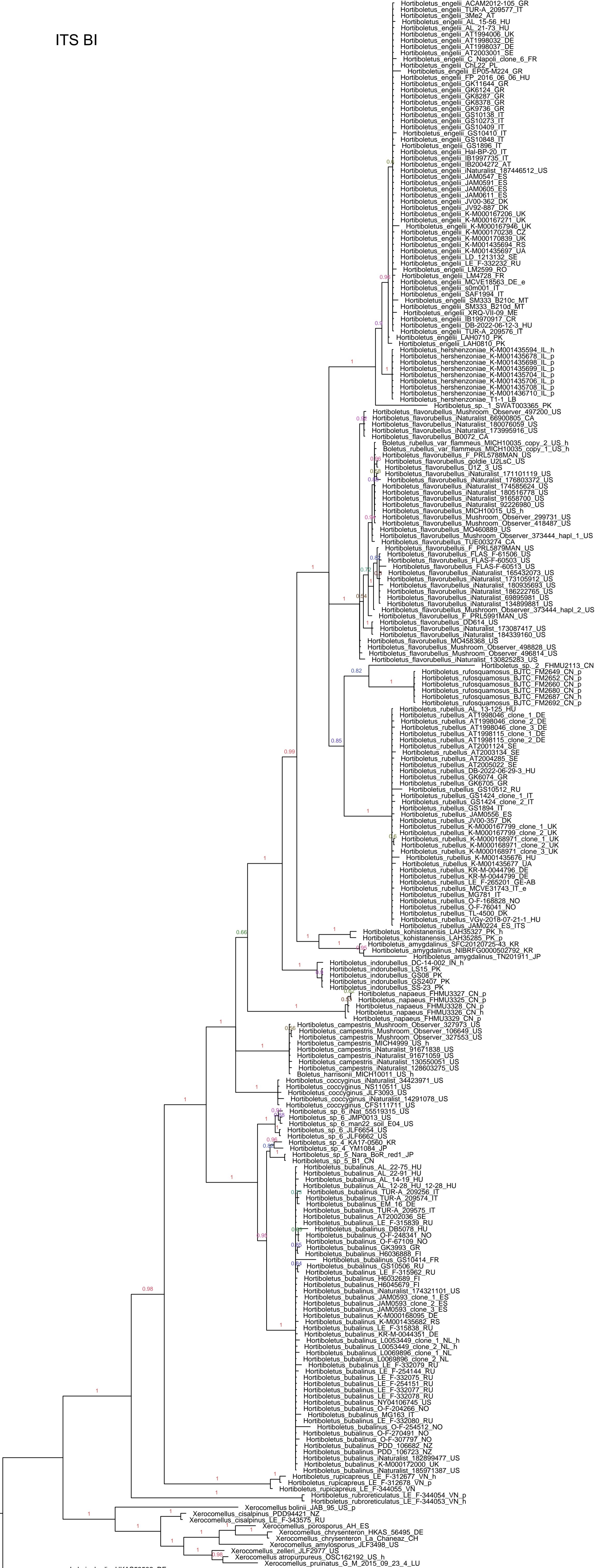

0.05

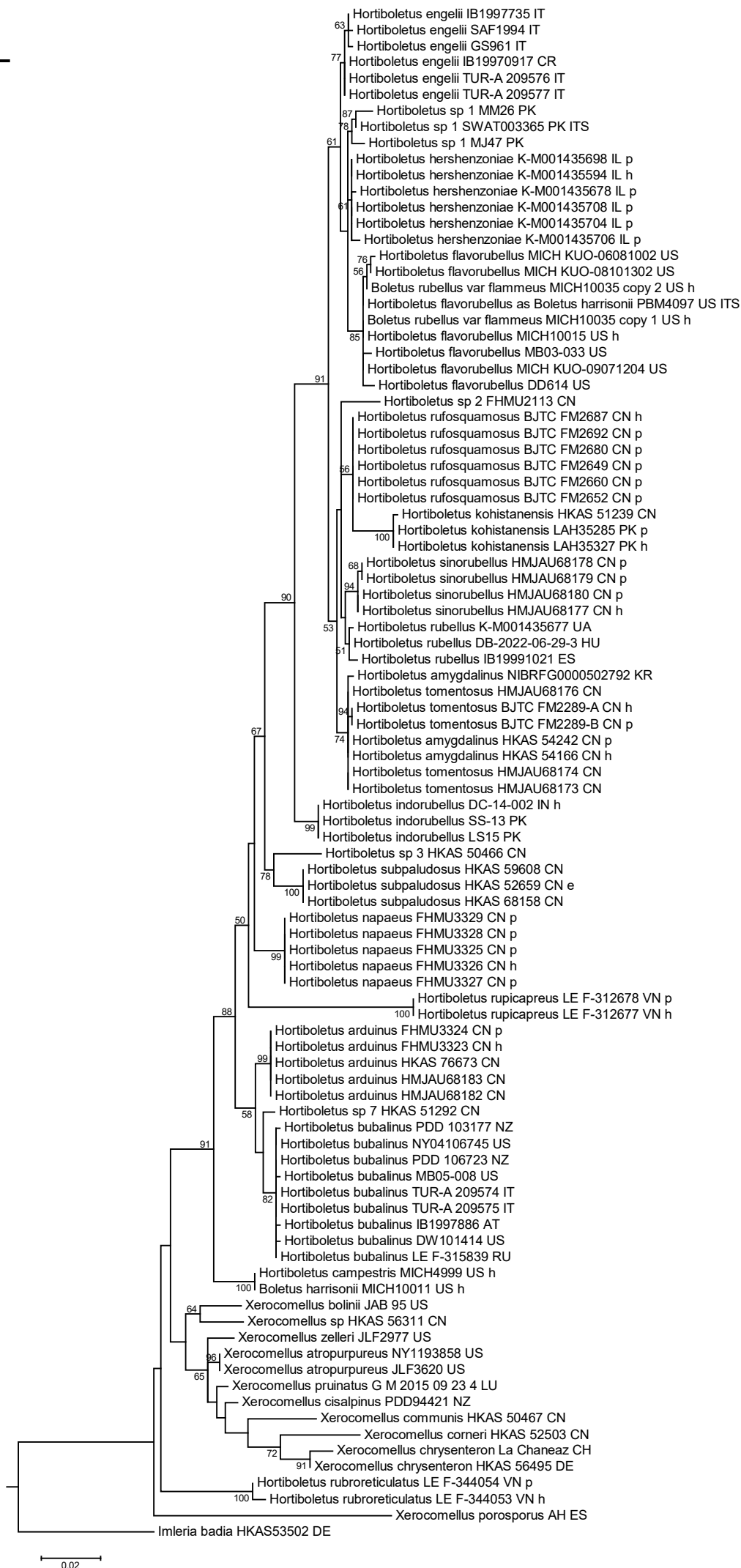

LSU BI

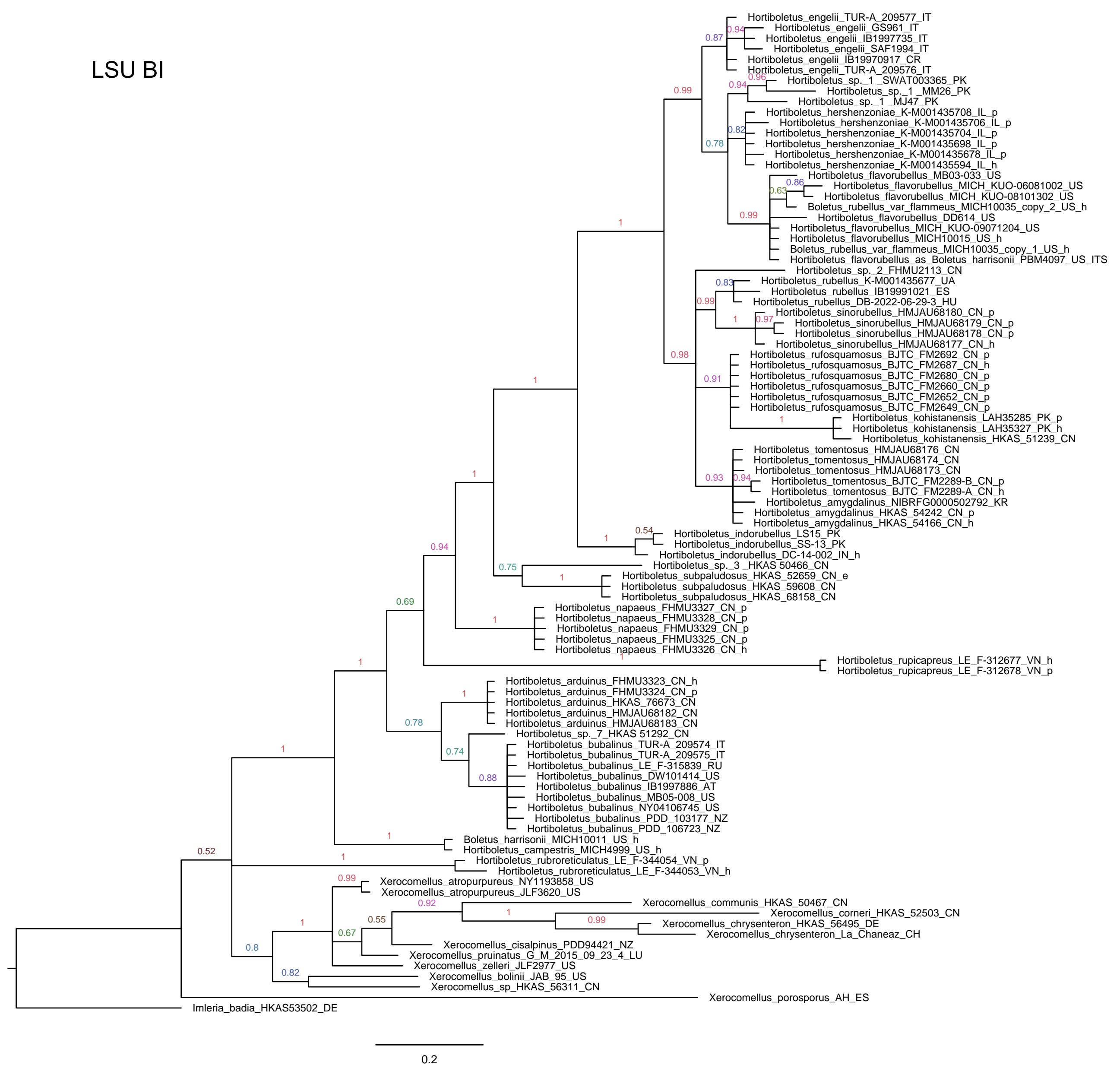

tef1-α ML

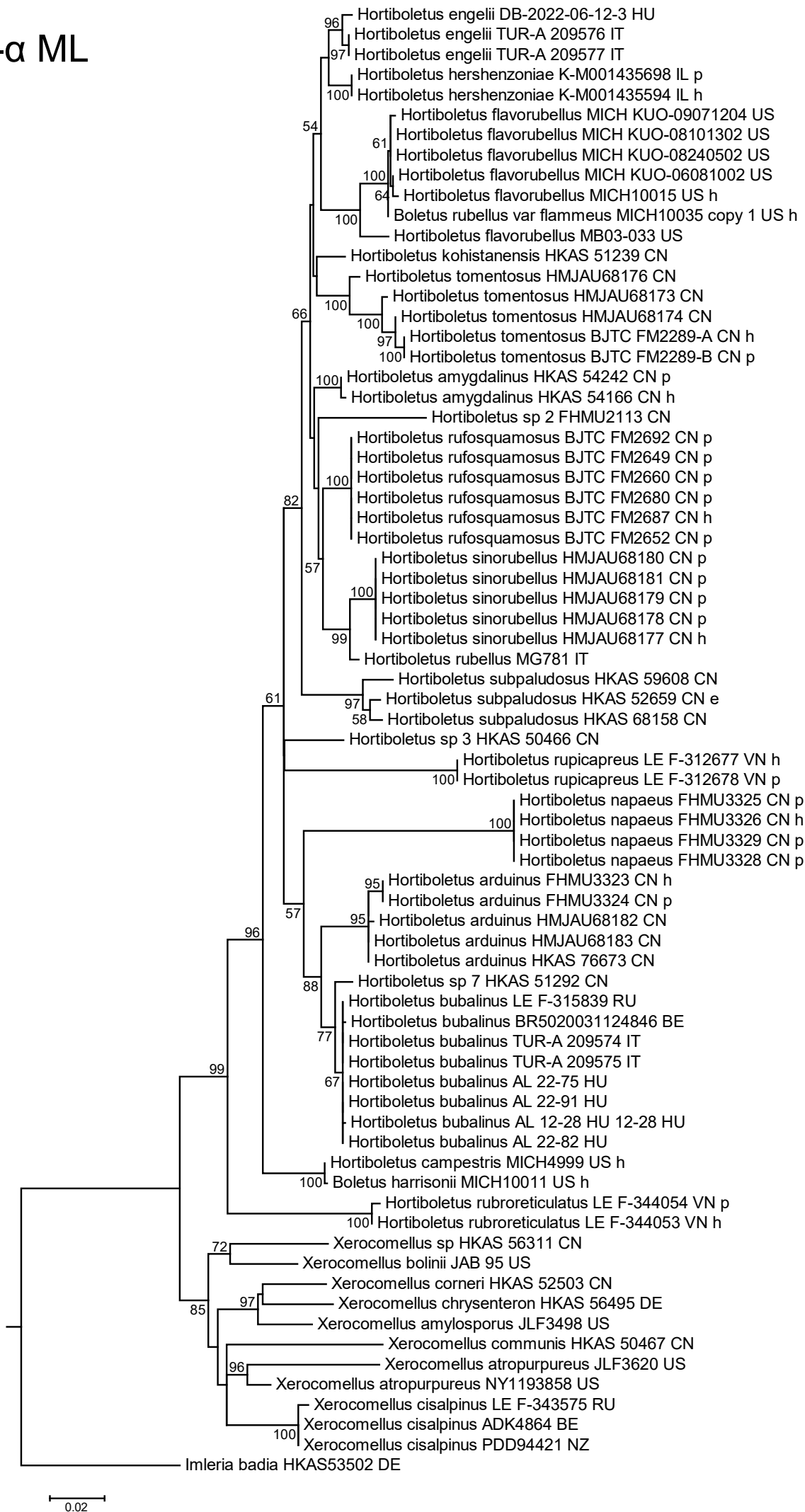

tef1- $\alpha$  BI

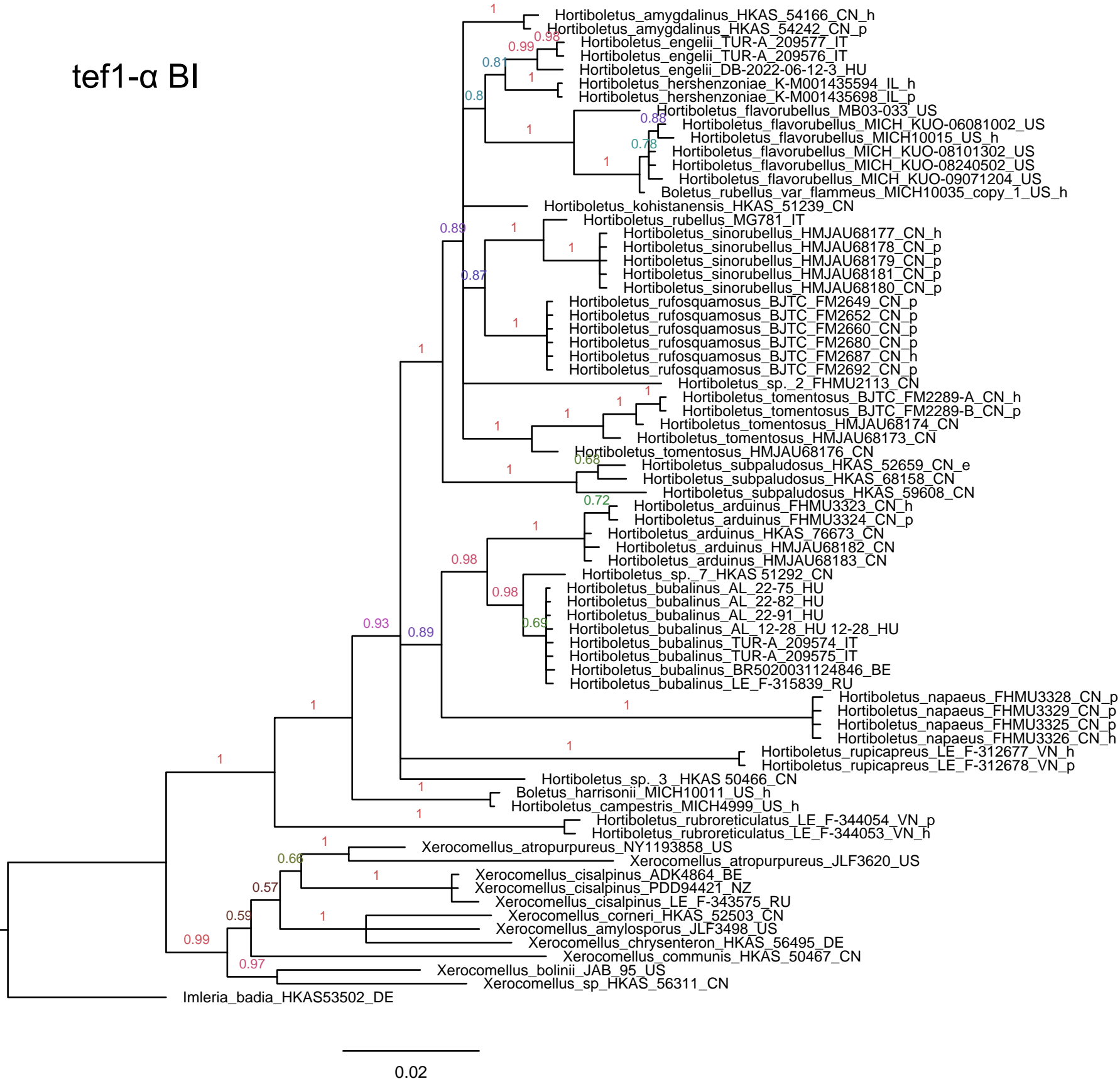

# rpb2 ML

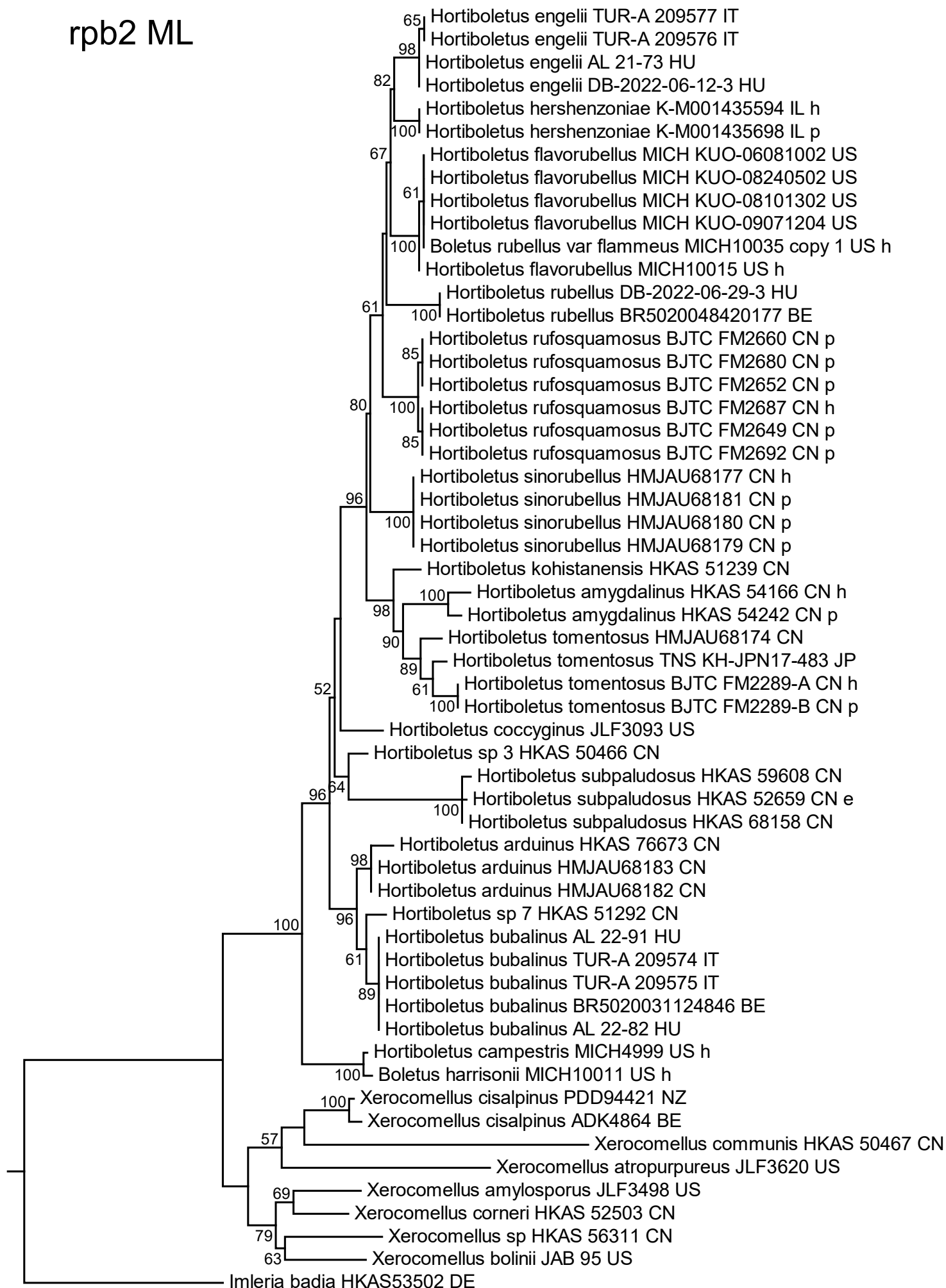

0.02

rpb2 BI

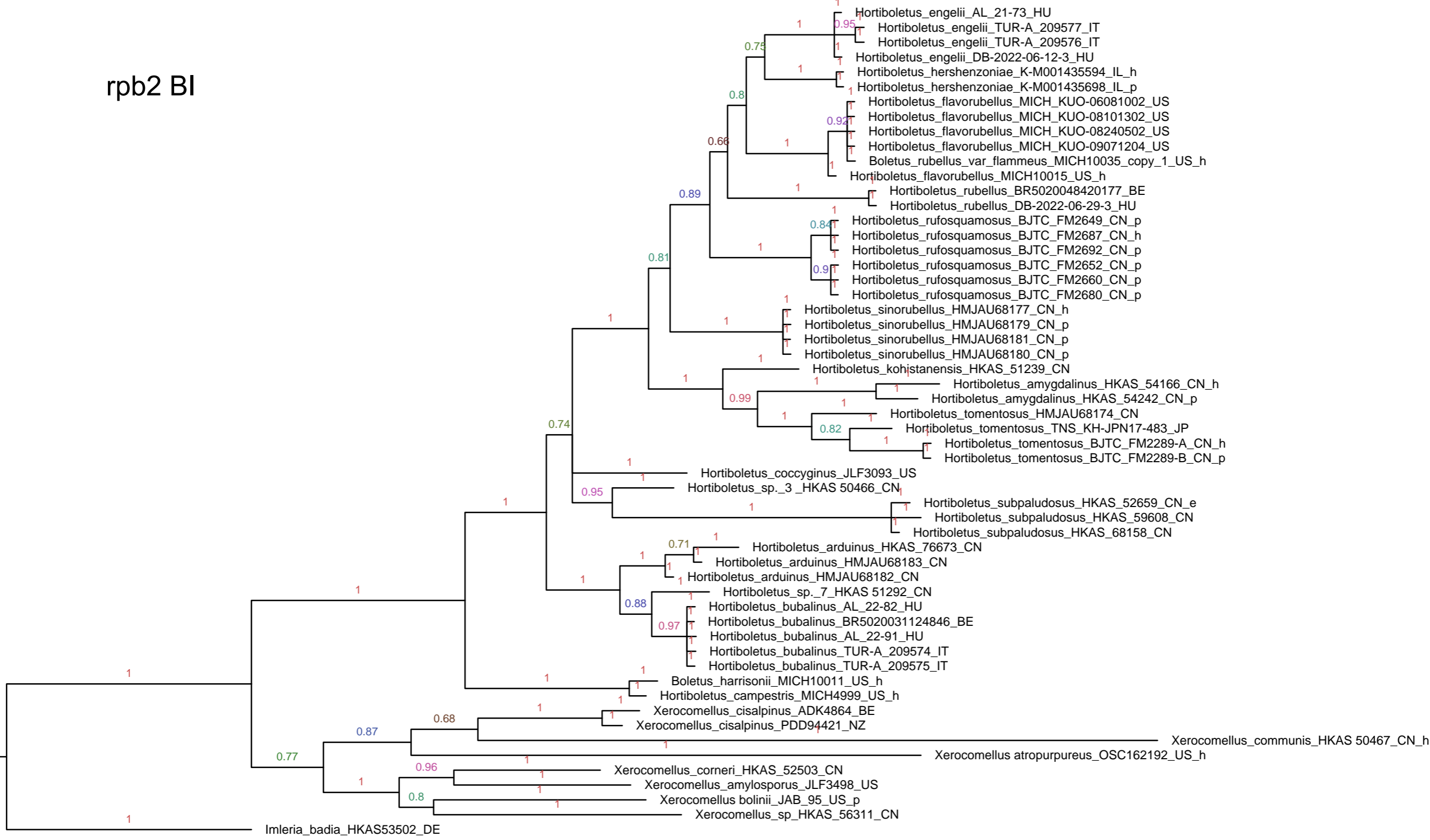

0.02

Supplement: Supplementary material 3 — Non-collapsed ML and BI phylogenetic trees: ITS, LSU, tef1-α, and rpb2 [file imafungus-16-e144731-s003.pdf]
